# Structure and evolution of the filaggrin gene repeated region in primates

**DOI:** 10.1186/s12862-016-0851-5

**Published:** 2017-01-11

**Authors:** Vanessa Romero, Kazuyoshi Hosomichi, Hirofumi Nakaoka, Hiroki Shibata, Ituro Inoue

**Affiliations:** 1 Department of Genetics, School of Life Sciences, Graduate University for Advanced Studies (SOKENDAI), Mishima, 411-8540 Japan; 2 Division of Human Genetics, National Institute of Genetics, Mishima, 411-8540 Japan; 3 Division of Genomics, Medical Institute of Bioregulation, Kyushu University, Fukuoka, 812-8582 Japan; 4 Present address: Department of Bioinformatics and Genomics, Graduate School of Medical Sciences, Kanazawa University, Kanazawa, 920-8640 Japan

**Keywords:** Filaggrin, Copy number variation, Birth-and-death evolution, Duplication/loss

## Abstract

**Background:**

The evolutionary dynamics of repeat sequences is quite complex, with some duplicates never having differentiated from each other. Two models can explain the complex evolutionary process for repeated genes—concerted and birth-and-death, of which the latter is driven by duplications maintained by selection. Copy number variations caused by random duplications and losses in repeat regions may modulate molecular pathways and therefore affect phenotypic characteristics in a population, resulting in individuals that are able to adapt to new environments. In this study, we investigated the filaggrin gene (*FLG*), which codes for filaggrin—an important component of the outer layers of mammalian skin—and contains tandem repeats that exhibit copy number variation between and within species. To examine which model best fits the evolutionary pathway for the complete tandem repeats within a single exon of *FLG*, we determined the repeat sequences in crab-eating macaque (*Macaca fascicularis*), orangutan (*Pongo abelii*), gorilla (*Gorilla gorilla*), and chimpanzee (*Pan troglodytes*) and compared these with the sequence in human (*Homo sapiens*).

**Results:**

In this study we compared concerted and birth-and-death evolution models, commonly used for gene copies. We found that there is high nucleotide diversity between filaggrin repeat regions, which fits the birth-and-death model. Phylogenetic analyses also suggested that independent duplication events created the repeat sequences in crab-eating macaques and orangutans, while different duplication and loss events created the repeats in gorillas, chimpanzees, and humans. Comparison of the repeat sequences detected purifying selection within species and lineage-specific duplications across species. We also found variation in the length of the repeated region within species such as chimpanzee and crab-eating macaque.

**Conclusions:**

We conclude that the copy number variation in the repeat sequences of *FLG* between primates may be a consequence of species-specific divergence and expansion.

**Electronic supplementary material:**

The online version of this article (doi:10.1186/s12862-016-0851-5) contains supplementary material, which is available to authorized users.

## Background

The evolution of gene families and tandem repeat regions has been a controversial issue in evolutionary genetics because most of the repeat regions do not follow the conventional divergent model. In this model, each duplicate acquires a new function and gradually separates. However, repeated genes often maintain their original functions and are more similar within species than among related species [1].

Two alternative models of evolution have been proposed to explain this nondivergent evolutionary pattern in repeated gene families: concerted and birth-and-death. In the concerted model, mutations that occur in one repeat spread to the other repeats by unequal crossover or gene conversion, maintaining homogeneity among the repeats. This results in a low level of diversity of repeats within a species, and the sequences of the repeats being more similar within a species than between species. In the birth-and-death model, new repeat genes diversify by silent nucleotide substitutions. Some repeats are maintained and amplified for a long time under purifying selection, whereas others are deleted or become nonfunctional as a result of deleterious mutations, which can lead to a change in the number of copies between species. Thus, there is a high level of diversity between repeats, and genes have a close interspecies pattern [1–4]. In this study, we applied these models to improve our understanding of the evolution of the filaggrin gene (*FLG*), which contains coding tandem repeats and copy number variations between and within species. We investigated whether the repeat sequences of *FLG* in primates have evolved according to the concerted or birth-and-death model.

*FLG* encodes profilaggrin protein which is dephosphorylated and degraded to monomeric filaggrin repeats and then further proteolyzed to aminoacids. Profilaggrin, filaggrin repeats and aminoacids are localized in the outer layers of the epidermis and have important functions in skin. Glutamine is converted into pyrrolidone-5-carboxylic acid (PCA) and histidine is deiminated to cis-urocanic acid (UCA). PCA and UCA serve important functions as protecting against UV irradiation, maintaining the acidic pH, being involved in the local immune response, and maintaining overall homeostasis [5, 6]. *FLG* consists of three exons, with the repeat region being found on the third. The filaggrin repeat region consists of complete repeats that are flanked by two partial repeats (Additional file 1: Figure S1). The complete repeats are responsible for the function of this protein in the skin. The number of complete repeats varies from 10 to 12 within human (*Homo sapiens*) individuals and populations. This copy number variation is associated with the quantity of filaggrin expressed in the epidermis [6–10]. Several nonsynonymous variants in the repeat region have also been reported to be associated with various dry skin disorders, particularly atopic dermatitis and ichthyosis vulgaris [7, 11].

Comparative genomic studies have revealed that the structure of *FLG* is similar in human, mouse (*Mus musculus*), and dog (*Canis lupus familiaris*). However, the number of complete repeats and the length of repeats differ between species [12, 13]. These differences can be recognized as an example of structural variations resulting from species-specific duplication or loss events. Structural variations are a major source of morphological differences, which may allow individuals to adapt to new environments and accelerate divergence between species [14–18]. Comparison of closely related species with short divergence times allows us to better understand the mechanisms leading to rapid divergence. In this study, we investigated variations in the repeat region of *FLG* among five species of primates: crab-eating macaque (*Macaca fascicularis*), orangutan (*Pongo abelii*), gorilla (*Gorilla gorilla*), chimpanzee (*Pan troglodytes*), and human and inferred whether the concerted model or the birth-and-death model best fits the evolutionary pathway for these complete tandem repeats.

## Results

### *FLG* sequences in primates

*FLG* is localized on chromosome 1 of crab-eating macaque, orangutan, gorilla, chimpanzee, and human. In human, it consists of three exons, with the repeat region being found on the third. Neither exons other than exon 3 nor introns have filaggrin repeated units. The DNA sequences of *FLG* were obtained from the National Center for Biotechnology Information (NCBI) gene database (http://www.ncbi.nlm.nih.gov/gene/) for the following primate species: *H. sapiens* (NC 000001.11), *P. troglodytes* (NC 006468.3), *G. gorilla* (NC 018424.1), *P. abelii* (NC 012591.1), and *M. fascicularis* (NC 022272.1). These were compared with the repeat region in human by carrying out a dot-matrix analysis using the Harrplot program, and the matched sequences were then compared with individual partial and complete repeats of human *FLG*. This allowed us to reconstruct the number and order of the repeats in these primate species.

**Fig. 1:**
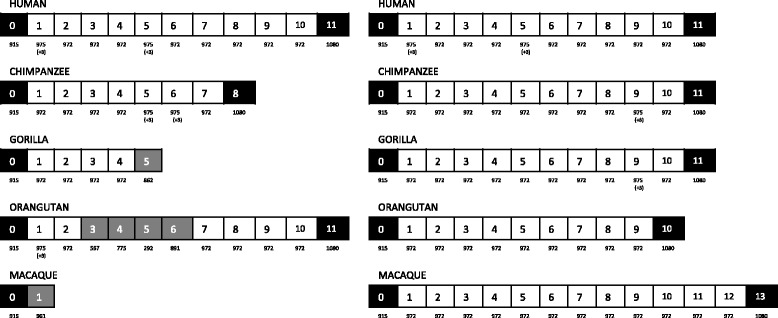
Partial and complete repeat sequences of *FLG* in human, chimpanzee, gorilla, orangutan, and crab-eating macaque. Left shows the repeat order of *FLG* in these primates based on the sequences from the National Center for Biotechnology Information database; the gorilla sequence had an unframed repeat at the end of the sequence, and the orangutan sequence had unframed repeats in the middle. Right shows the repeat order of *FLG* sequences acquired through the use of both PacBio RSII and MiSeq in this study. *Black* = partial repeats, *white* = complete repeats, and *gray* = regions with gaps. The nucleotide length of each repeat is shown beneath each repeat. The number of repeats varies between species

The estimated number of complete repeats differed between species, as shown in Fig. 1. It has previously been reported that the length of these complete repeats ranges from 972 to 975 bp [5]. Based on this, we noted that there were several gaps in some of the complete repeats in orangutan from the NCBI database, and one of the partial repeats was not identified in gorilla. Furthermore, the complete repeats with gaps resulted in unframed repeats, which may produce truncated proteins (Additional file 1: Figure S2A and B). This finding implies that the sequences of *FLG* for these primates that were obtained from the NCBI database are also likely to have been incomplete.

Sequencing of large and nearly identical repeated structures is a challenging task, and the presence of exonic copy number variation increases complexity of the sequence analysis [5]. Therefore, to overcome these difficulties and determine the complete nucleotide sequences of *FLG*, we combined two different sequencing platforms: PacBio RS II and Illumina MiSeq. The long reads (>8 kb) generated by PacBio RS II were used to determine the overall structure of the repeat regions as an initial reference. To compensate for the high error rate of PacBio RS II, reads generated by MiSeq were then mapped to the initial reference to determine error-corrected sequences of *FLG* for each of the primates [19, 20]. In the resulting sequences, there were no gaps in the complete repeats, and two partial repeats were retrieved for all species. The sequences we acquired altered the number of complete repeats to 10 repeats for human, chimpanzee, and gorilla, 9 repeats for orangutan, and 12 repeats for crab-eating macaque (Fig. 1). In the subsequent analyses, each of the repeats was considered as an independent unit.

### Phylogenetic analysis of complete repeats across species

**Fig. 2:**
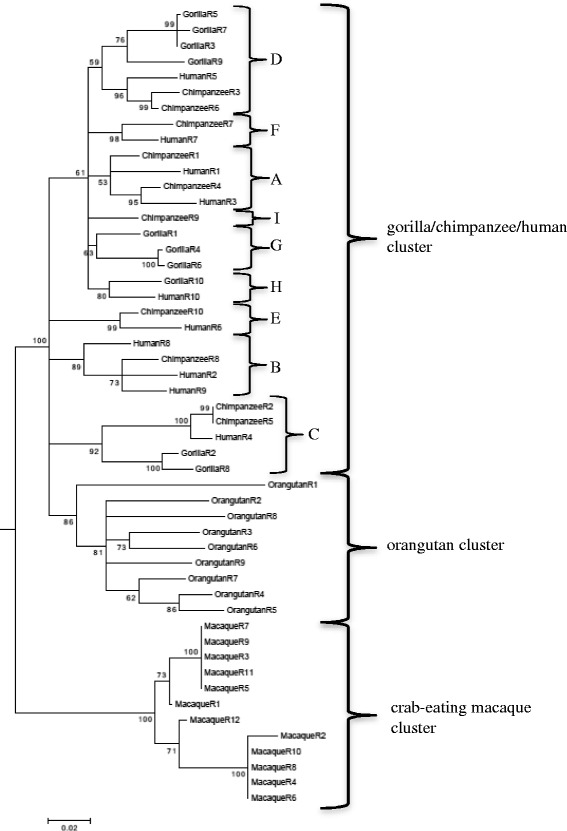
Maximum likelihood tree reconstruction using all complete repeats in these primates and the following parameters: partial deletion option, Tamura-Nei model with gamma distribution and invariable sites, nearest-neighbor-interchange heuristic method, 1000 bootstrap resampling, and a cutoff value of 50%. Bootstrap values are shown at the beginning of each branch. Crab-eating macaque was used as an outgroup. Crab-eating macaque repeats and orangutan repeats grouped into their own clusters (“crab-eating macaque cluster” and “orangutan cluster,” respectively). By contrast, the gorilla repeats, chimpanzee repeats, and human repeats are scattered across the “gorilla/chimpanzee/human cluster.” We divided the “gorilla/chimpanzee/human cluster” into subclusters in accordance with the reconciled tree reconstruction shown in Fig. 3

Based on the results of the multiple sequence alignment for all of the repeats (Additional file 1: Table S2A and B), we constructed phylogenetic trees using the maximum parsimony, neighbor-joining, and maximum likelihood methods, with a cutoff value of 50% due to the high similarity of the repeats, as described in the Methods section. Crab-eating macaque was used as an out-group (Fig. 2; Additional file 1: Figure S3A and B).

All three methods gave similar phylogenetic trees. All complete repeats in crab-eating macaque and orangutan grouped into their own clusters (“crab-eating macaque cluster” and “orangutan cluster,” respectively), whereas the complete repeats in gorilla, chimpanzee, and human scattered across species in a large cluster (“gorilla/chimpanzee/human cluster”) (Fig. 2). This large cluster contained nine subclusters of repeats among species. However, with the exception of subclusters “F” and “H”, their counterparts were not in order—for example, chimpanzee-repeat 4 gathered with human-repeat 2 (Fig. 2). To further confirm the intra and inter species clustering in primates, we added repeats of mouse and dog and then constructed maximum parsimony, neighbor-joining, and maximum likelihood methods, with a cutoff value of 50% and observed the same phylogeny (Additional file 1: Figure S4A, B and C).

The clusters of crab-eating macaque and orangutan repeats suggest that unique ancestral repeats were duplicated in these two primate species. The finding that human, chimpanzee, and gorilla repeats clustered together, but with limited conservation in the order of the repeat sequences, suggests that these three species share several ancestral repeats, and that random duplication and loss events occurred independently in each of the three species.

The main difference between the concerted and birth-and-death models of evolution is the high levels of intragenic nucleotide diversity that are only found in the latter [1–4]. We calculated the total number of sites showing variation and the nucleotide diversity (*π*) between complete repeats of *FLG* using the DNAsp5 program, as described in the Methods section. The estimated *π* for human, chimpanzee, gorilla, orangutan, and crab-eating macaque was 7.7 × 10^−2^, 7.5 × 10^−2^, 6.1 × 10^−2^, 7.7 × 10^−2^, and 3.2 × 10^−2^, respectively (Table 1). We then compared the *π* of *FLG* with that of polyubiquitin genes and rDNA genes, which are representative multigene families that have evolved under birth-and-death evolution and concerted evolution, respectively [2, 21]. The *π* of *FLG* was comparable with that of polyubiquitin genes (range = 88.6 × 10^−3^ to 197 × 10^−3^) and much larger than those of rDNA genes (range = 0.01 × 10^−3^ to 0.18 × 10^−3^) [2, 21]. This suggests that *FLG* repeats have evolved under the birth-and-death model.

**Table 1:** Comparison of all repeat sequences of the filaggrin gene within five primate species

Species	No. of repeats	Total no. variation sites	Average (1 - Ka/Ks) × 100^a^	Nucleotide variation
Human	10	195	31.49	0.077
Chimpanzee	10	189	38.91	0.075
Gorilla	10	140	29.90	0.061
Orangutan	9	198	33.19	0.077
Crab-eating macaque	12	69	70.96	0.032

^a^(1 - Ka/Ks) × 100 measures the percentage of purifying selection

### Selection within the repeats of each species

The driving force that maintains repeats in the birth-and-death model is selection. Under purifying selection, the number of nonsynonymous variations in a gene is expected to be smaller than the number of synonymous variations. Purifying selection can be measured by making pairwise comparisons ((1 − *K*a/*K*s) × 100), which indicate the percentage of synonymous mutations [22].

We found that the average percentage of synonymous substitutions for human, chimpanzee, gorilla, orangutan, and crab-eating macaque was 31.49, 38.91, 29.90, 33.19, and 70.96%, respectively (Table 1). Although nucleotide substitutions were generally synonymous, we found 12 pairs of complete repeats within species with higher nonsynonymous variations: two pairs in human repeats (R3–R7 and R3–R10), two pairs in chimpanzee repeats (R3–R6 and R3–R10), and eight pairs in gorilla repeats (R1–R8, R2–R7, R2–R8, R2–R9, R3–R8, R5–R8, R7–R8, and R8–R9). None of the pairs of repeats in crab-eating macaque and orangutan had *K*a/*K*s > 1 (Additional file 1: Table S4).

### Codons and branches under selection across species

In humans, it has been suggested that several amino acid substitutions of *FLG* may have evolved under positive selection [5, 8, 23]. While homozygous null-mutations of *FLG* are associated with a variety of skin disorders, individuals expressing heterozygous null-mutations show a less severe phenotype and could acquire immunity via the skin barrier. Population-specific mutations have also been reported, which rules out genetic drift [5, 23]. In this study, we detected high *K*a/*K*s ratios (>1) in our within-species comparisons. Therefore, we investigated whether similar variations in *FLG* were also present in other primates by searching for signatures of positive selection in the complete repeats across species using phylogenetic analysis by maximum likelihood (PAML) software [4, 24, 25], as described in the Methods section.

First, we carried out two site-based tests for each codon of the complete repeats, in which we compared models with positive selection and neutral/negative selection (M1a (nearly neutral) vs M2a (positive selection) models and M7 (beta) vs M8 (beta and *ω*)). In both of these tests, the results supported the presence of positive selection for 14.13 and 10.8% of codons, respectively; in *FLG* complete repeats (Table 2).

**Table 2:** Positively selected codons and branches of the filaggrin gene in five primate species

Site-based test		
Models compared	*P* value	Positively selected codons
M1a vs M2a^a^	1.40 E-42	21, 24, 26, 75, 99, 110, 135, 144, 150, 157, 178, 187, 190, 191, 224, 226, 228, 231, 252, 268, 269, 305, 323
M7 vs M8^a^	1.05 E-50	4, 21, 24, 26, 75, 99, 110, 127, 135, 144, 150, 152, 157, 178, 187, 190, 191, 205, 224, 226, 228, 231, 245, 252, 268, 269, 305, 309, 320, 323
Branch-based test		
Models compared	*P* value	
M0 vs free ratio^b^	0.13	–
Branch-site test		
M0N0 vs M2N2^c^	*P* value	Positively selected codons
Crab-eating macaque cluster	1.27 E-60	No sites
Orangutan cluster	2.54	26, 119, 160, 177, 179, 207, 250, 284
Gorilla/chimpanzee/human cluster	1.80 E-93	24, 26, 78, 99, 110, 114, 134, 135, 144, 150, 152, 157, 169, 178, 187, 191, 224, 228, 231, 245, 268, 320, 323

^a^The site-based test compared the M1a (nearly neutral) and M2a (positive selection) models and the M7 (Beta) and M8 (Beta and *ω*) models

^b^The branch-based test compared the free ratio (or independent *ω* per branch) model with the one-ratio null (or one *ω* for all branches) model (M0)

^c^The branch-site test was used to detect positively selected codons on a specific branch

Second, we investigated whether selective pressures differed between branches of the phylogenetic tree by a branch-based test. The result of this test was not significant, suggesting that each branch has not evolved independently, and that either all branches have evolved at the same rate or certain branches have evolved at similar rates (Table 2).

Third, we performed branch-site tests to detect signatures of positive selection at specific branches. Since the phylogenetic analyses outlined previously showed that the complete repeats were grouped into three clusters (crab-eating macaque cluster, orangutan cluster, and gorilla/chimpanzee/human cluster), we searched for the positively selected codons in the main branches of these clusters. We identified positively selected codons in the branches of the orangutan cluster and gorilla/chimpanzee/human cluster (Table 2), with the signature of positive selection identified at codon 26 in both of these branches. We used the repeat sequences aligned to human-repeat 1 when we performed the search for signatures of positive selection by maximum likelihood (PAML) software; therefore the positively selected codon refers to codon 26 in human-repeat 1. The known functional domains of filaggrin are located in a linker region at amino acids 11–20 and a cleavage region for caspase-14 at amino acids 162–165 or 171–174 [23, 26, 27]. The positively selected amino acids identified in this study were not located in these domains (Table 2). However, it is possible that the positively selected codons detected in this study are located in as-yet-unknown functional domains.

### Crab-eating macaque and orangutan have evolved under the birth-and-death model by recent gene duplication

Under the birth-and-death model of evolution, multigene families are not only expected to have high levels of nucleotide variation but also an interspecies gene-clustering pattern in the phylogenetic analysis. We only found high nucleotide variation, purifying selection, and an interspecies repeat cluster in the complete repeats in gorilla, chimpanzee, and human. The other two species (crab-eating macaque and orangutan) had high nucleotide variation and purifying selection but an intraspecies repeat clusters.

**Fig. 3:**
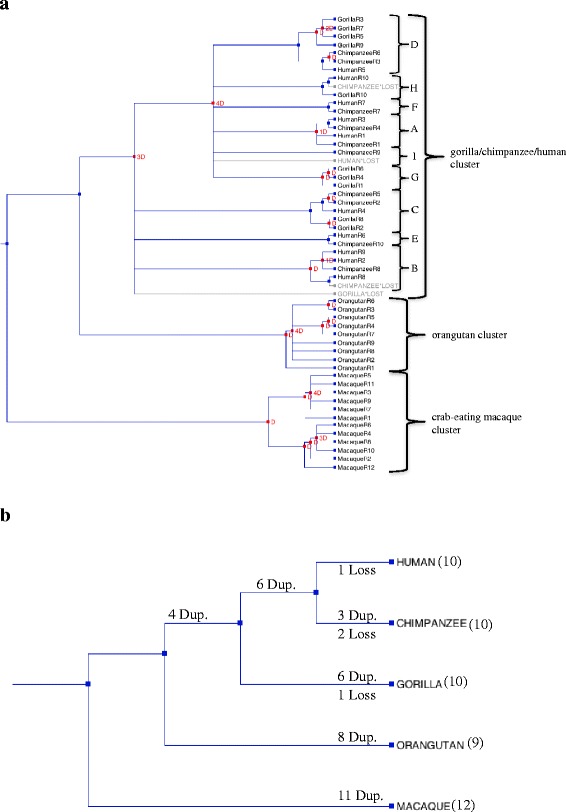
“Reconciled” trees using complete repeats of *FLG* in human, chimpanzee, gorilla, orangutan, and crab-eating macaque. **a** “Reconciled” gene tree indicating duplication (*red squares*) and loss events (*light gray italics*) from the most common ancestor of these primates: the crab-eating macaque repeats duplicated 11 times, orangutan repeats duplicated 8 times, and gorilla/chimpanzee/human repeats duplicated 18 times, and in human, the counterpart to chimpanzee-repeat 9 was lost, while in chimpanzee, the counterparts to human-repeats 10 and 8 were lost, and in gorilla, the counterpart to human-repeat 2, 9 and 8 and chimpanzee-repeat 8 was lost. **b** “Reconciled” species tree indicating duplication and loss events in each species from their most common ancestor: crab-eating macaque repeats duplicated 11 times, orangutan repeats duplicated 8 times, gorilla repeats duplicated 6 times, and chimpanzee repeats duplicated 3 times, while the most common ancestor between human and chimpanzee duplicated 6 times, and the most common ancestor between human, chimpanzee, and gorilla duplicated 4 times; and 1 repeat was lost in gorilla, 2 repeats were lost in chimpanzee, and 1 repeat was lost in human. The number of repeats found in each species is provided in parentheses

**Fig. 4:**
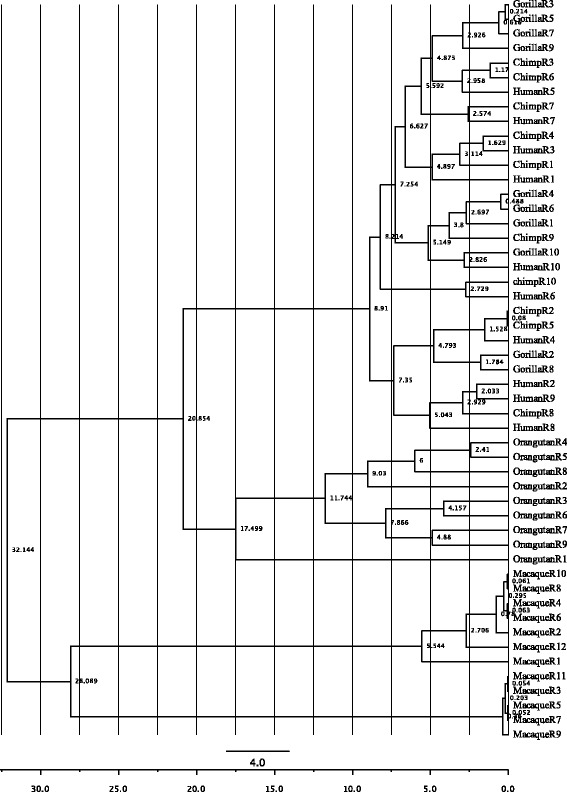
“Divergence” tree reconstruction using all complete repeats of *FLG* in five primate species. The following parameters were used: the site model TN93, a substitution rate with gamma distribution = 4, a log-normal relaxed clock, and the birth-and-death model for all three nucleotide positions. The *x*-axis scale is time in Mya. The crab-eating macaque ancestor repeat diverged around 28 Mya, while the orangutan ancestor repeat diverged around 21 Mya. The gorilla/chimpanzee/human repeats duplicated during the last 9 Mya, while the crab-eating macaque repeats duplicated in the last 5.5 Mya

Intraspecies repeat clusters can occur if the repeats are under recent duplication. Therefore, to further characterize the evolutionary process behind the intraspecies repeat clusters detected in crab-eating macaque and orangutan, we constructed reconciled and divergence trees [28–30], the parameters for which are described in the Methods section. These two trees suggest that the original crab-eating macaque repeat duplicated 28 Mya, and that each branch has undergone a series of five duplications in the past 5.5 Mya. By contrast, the original orangutan repeat separated 21 Mya, and the current nine repeats were created by one divergence event and seven subsequent duplications during the last 18 Mya (Figs. 3a, b and 4).

**Fig. 5:**
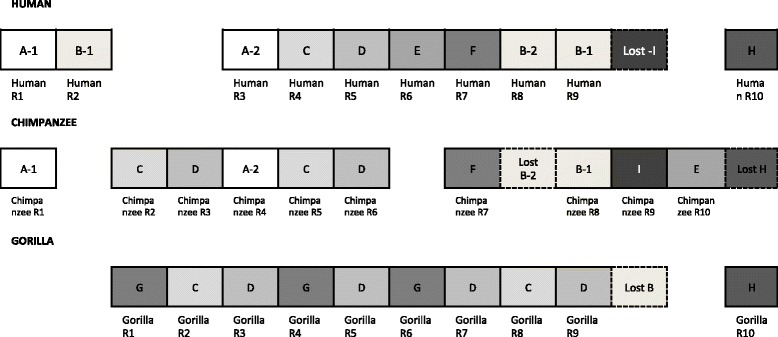
Repeat order of gorilla, chimpanzee, and human *FLG* repeats, as inferred by phylogenetic and “reconciled” tree. Gorilla duplicated repeats “C,” “D,” and “G,” chimpanzee and human duplicated repeat “A,” and human duplicated repeat “B” are shown. In chimpanzee, the counterpart to human-repeats B-2 has been lost, while in human, the counterpart to chimpanzee-repeat “I” has been lost, and in chimpanzee, the counterparts to human and gorilla-repeat H have been lost. In gorilla, the counterpart to human and chimpanzee repeats B has been lost

We also found that gorilla, chimpanzee, and human have their own duplication events. For example, the four repeats in gorilla (R3, R5, R7, and R9) gathered within the gorilla/chimpanzee/human cluster in the phylogenetic tree (Fig. 3a and b). By combining the information about the subclusters from the phylogenetic tree with the duplication and loss events from the reconciled tree, we were able to infer the evolution of the complete repeats in each species. We named each of the subclusters from “A” to “I,” according to the order of the repeat sequences in human, for example, the subcluster containing the first repeat in human was defined as subcluster “A.” We found that the order of these subclusters was largely conserved between human and chimpanzee (Fig. 5). In contrast, the order of the subclusters in gorilla differed from those of human and chimpanzee, suggesting specific duplications in this species (Fig. 5). Here we named chimpanzee-repeat 9 as “I”, this repeat did not cluster with any other repeats but the reconciled analysis showed that the counterpart of human was lost. These results suggest that during a period of divergence, each species has gone through independent duplication and loss events, which has led to the creation of a unique set of repeats and their own clusters.

### Repeat variation found in crab-eating macaque and chimpanzee

In human, the number of *FLG* complete repeats varies from 10 to 12 within individuals and populations [6, 7]. Taking the variation described in human into consideration, we performed PCR of the repeated region for additional primate samples. Gel electrophoresis result showed that the size in chimpanzee and macaque samples varied suggesting the possibility of repeat variation in these species as seen in human (Additional file 1: Figure S6).

## Discussion

The repeat region of *FLG* consists of nearly identical complete repeats. However, the number of complete repeats differs between species, as previously reported in mouse and dog [12, 13]. In this study, we demonstrated that the DNA sequences obtained from the NCBI database were not complete and contained several gaps. Therefore, we performed re-sequencing at *FLG* in four primate species using PacBio RSII and MiSeq and were able to resolve the gaps and determine the full length of the repeat sequences of *FLG* in each species. This led to the detection of 10 repeats for human, chimpanzee, and gorilla, 9 repeats for orangutan, and 12 repeats for crab-eating macaque (Fig. 1).

It has previously been reported that gene-associated tandem repeats act as an accelerator of evolution by generating variation in structure and functionality [15]. Birth-and-death model has been used to explain multigene families evolution. Under the birth-and-death model, duplicates vary by silent nucleotide variations, which, with enough divergence time, can lead to lineage-specific expansions. We detected this pattern of evolution in the repeats of crab-eating macaque and orangutan using both reconciled and divergence trees (Figs. 2, 3a and b, and 4) and was corroborated by including the repeats of mouse and dog in the reconciled tree (Additional file 1: Figure S4D) [12, 13]. The clusters of species-specific repeats may reflect the high nucleotide diversity together with duplication and loss events in each species (Table 1), which seems to fit the birth-and-death model [4, 21, 22]. The main driving force of the birth-and-death model are duplications maintain by selection, which we found evidence for in *FLG*. Macaque repeats had the lowest nucleotide variation, the highest purifying rate and interspecies gene-clustering pattern in phylogeny analyses, most likely due to the recent repeat duplication that we reported (Table 1; Figs. 3 a, b, 4 and 5; Additional file 1: Table S4). High synonymous variation has previously been found in most genes under the birth-and-death model of evolution, including actin in flagellate protists (dinoflagellates) and the ubiquitin gene family [21, 31]. Under the birth-and-death model of evolution, duplication and loss events are not caused by gene conversion. Gene conversion is an unlikely event for *FLG* repeats because they are located in one exon, and there is no gene family. We examined several algorithms for detection of recombination events and could not identify any major recombination event (Additional file 1: Table S5) [32, 33]. In addition, we removed the putative recombinant regions and reconstructing the phylogenetic tree resulted in only a few changes to the topology, confirming the low possibility of gene conversion (Additional file 1: Figure S5) [34]. A similar finding has previously been reported for the NAT gene family, in which there was no evidence of gene conversion following the combined use of phylogenetic analysis and fine-scale synteny mapping [4]. The birth-and-death model suggests but does not require the presence of pseudogenes. In the NCBI gene database we did not find any filaggrin pseudogenes that include repeats but there are two types of genes that are similar to the complete repeats of filaggrin; filaggrin-2 and filaggrin-like. Filaggrin-2 repeats are shorter than complete filaggrin repeats (225 bp) and we did not consider filaggrin-2 for further analysis. We found filaggrin and filaggrin-like repeats in crab-eating macaque evolved under a similar birth-and-death model with strong selective constrains and positively selected codons were not detected, that is further described in Additional file 1: Supplementary information (Additional file 1: Figure S2C, D and E, Table S6, S7 and S8).

Several studies have demonstrated that copy number variations due to the duplication or loss of repeats are important in modulating molecular pathways. This includes morphological modifications in dog breeds and craniofacial or digit defects in humans, which have been shown to be due to variation in the length of developmental genes [15, 35]; an increased in the risk for schizophrenia; and several complex diseases in human caused by heterozygosity [14, 36] or dosage compensation [37]. Additionally, in the case of new gene copies, duplicates under strong purifying selection at the protein level lead to functional divergence of new copies through a process of neofunctionalization or subfunctionalization [3]. *FLG* encodes profilaggrin, a long polyprotein. Profilaggrin is dephosphorylated and cleaved into filaggrin repeat units. In the outer layers of skin, the repeat units of filaggrin are degraded into free amino acids that are a major determinant of the natural moisturizing factor and maintain the skin overall homeostasis [6]. The amount of filaggrin-degraded aminoacids can be altered by *FLG* loss-of function mutations and variation in the number of complete repeats. Loss-of-function mutations strongly associate with atopic dermatitis. Atopic dermatitis patients with null-mutations were reported to have decreased levels of PCA and UCA [6–8]. The number of *FLG* complete repeats varies from 10 to 12 within human. We found variation in the length of the repeated region within species such as chimpanzee and crab-eating macaque and suggest the possibility of repeat variation in these species as seen in human (Additional file 1: Figure S6). In human, it was previously reported that the variation in repeats are due to duplicates of repeat 8 and 10. We designed primers for a product from human-repeat 7 to human-repeat 11 and gel electrophoresis showing more clearly this variation (Additional file 1: Figure S6). The variation in the number of repeats affects filaggrin degradation products. Increased number of FLG repeats associate with a decreased risk for atopic dermatitis [6, 7]. Therefore, random copy number variation in the *FLG* repeats within individuals could affect filaggrin degradation products and its functions, and will allow them to adapt more readily to a new environment [22]. In human, the effect of repeat variation was explained as mentioned before; however similar skin disorder or the functional significance of the repeat variation within primates has not been reported. Therefore, further studies are required to verify the functional significance of copy number variation of FLG repeats in primates.

## Conclusions

We concluded that the DNA sequences obtained from the NCBI database were not complete and contained several gaps and re-sequenced them using PacBio RSII and MiSeq. This led to the detection of a different number of repeats for chimpanzee, gorilla, orangutan and crab-eating macaque. Also we concluded that *FLG* repeats evolved under the birth-and-death model, by showing species-specific clusters in crab-eating macaque and orangutan; high nucleotide diversity together with duplication and loss events in each species and the unlikeliness of gene conversion. We conclude that the copy number variation in the complete repeats of *FLG* across primates is a consequence of species-specific expansions following a long period of divergence.

## Methods

### Database search for primate *FLG* sequences

We obtained full-length *FLG* DNA sequences from the NCBI gene database (http://www.ncbi.nlm.nih.gov/gene/) for the following primates: *H. sapiens* (NC 000001.11), *P. troglodytes* (NC 006468.3), *G. gorilla* (NC 018424.1), *P. abelii* (NC 012591.1), and *M. fascicularis* (NC 022272.1). In addition we obtained *FLG-like* DNA sequences for *P. troglodytes* (NW 003460518.1) and *M. fascicularis* (NW 005093011.1 and NC 022274.1).

### Primate samples

*Macacca fascicularis* (ID: 181 and 246) and *Macacca mulatta* (ID: 725 and 970) samples were derived from cell line established from PBC by EBV transformation provided by Dr. Takafumi Ishida from the Department of Biological Sciences, Graduate School of Science, University of Tokyo. Two *G. gorilla* DNA samples (Primate ID: 1943 and 3846) were provided by Dr. Osamu Takenaka from the Primate Research Institute of Kyoto. Five *Pongo pygmaeus,* one *G. gorilla* and fourteen *P. troglodytes* DNA samples were obtained through the Great Ape Information Network (GAIN): *P. pygmaeus* (GAIN ID: 0091, 0031) from Tennoji Zoo, *P. pygmaeus* (GAIN ID: 0110) from Kobe City Zoo, *P. abelli* (GAIN ID: 0010) from Nagoya Higashiyama Zoo, *G. gorilla* (GAIN ID: 0080) from Ehime Tobe Zoo, and *P. troglodytes* (GAIN ID: 0143, 0158, 0170, 0212 0204, 0211, 0131, 0279, 0169, 0276, 0159, 0345 and Primate ID: 954 and 956) from Sanwa Kagaku Primate Park. All of the aforementioned zoos and parks are located in Japan. Human samples were Ecuadorian and recruited at Centro de la Piel, Quito-Ecuador, after the approval of the Ethics Committee of Centro de la Piel (CEPI 141014). Centro de la Piel follows the Sociedad Ecuatoriana de Bioética regulations. All the participants provided written informed consent. The Ethics Committee of National Institute of Genetics (nig 1419, 2014.11) approved the study protocol and the management of all samples.

### Sequencing of *FLG* in primates using PacBio RSII and MiSeq

We performed long-range PCR of *FLG* for the crab-eating macaque, orangutan, gorilla, and chimpanzee samples. Primers were designed at specific conserved regions in each species and the human repeat variation region, information about which is summarized in Additional file 1: Table S1. PCR reactions were performed using a PrimeSTAR® GXL DNA Polymerase kit, with a total reaction volume of 10 μl that included: genomic DNA (20 ng), PrimeSTAR GXL 5× buffer, dNTP mixture (200 μM), forward and reverse primers (0.2 μM each), PrimeSTAR GXL polymerase (0.25 U/10 μl), and water. The PCR conditions were as follows: 94 °C initial denaturation for 2 min, followed by 30 cycles each of denaturation (98 °C for 10 s) and elongation (68 °C for 10 min).

PacBio RS II (Pacific Biosciences, California, USA) is a single molecule, real-time DNA sequencing system that provides exceptionally long read lengths. We used all sequence reads generated by PacBio RSII that were longer than 8 kb. For the *de novo* and reference analyses, we used the Amplicon module in SMRT Portal and the Hierarchical Genome Assembly Process [19].

The MiSeq system (Illumina, San Diego, California, USA) was employed to generate high-throughput short reads for the identification of sequence variants. The DNA libraries were sequenced on the MiSeq platform with 350- and 250-bp paired-end modules.

We performed PacBio RSII and MiSeq system sequencing for the following samples *Macacca fascicularis* (ID: 181) derived from cell line established from PBC, the two *G. gorilla* DNA samples (Primate ID: 1943 and 3846) provided by Dr. Osamu Takenaka, *P. pygmaeus* (GAIN ID: 0091) and *P. troglodytes* (GAIN ID: 0143, 0158, 0170, and 0212) samples. The datasets supporting the conclusions of this article are available in DDBJ database, http://www.ddbj.nig.ac.jp/. Identification number are as follow; macaque (LC096129), orangutan (LC096130), gorilla (LC096131), and chimpanzee (LC096129).

### Identification of *FLG* repeats in primates

We performed a dot-matrix analysis to detect repeat *FLG* sequences in primates using the Harrplot 2.1.8 program from GENETYX Corporation. Initially, we compared the *FLG* sequence in human with that in crab-eating macaque, orangutan, gorilla, and chimpanzee. The matched sequences were then reanalyzed to search for possible repeat regions. Finally, we manually curated the possible repeat regions to determine the correct repeat sequences. Each of the identified repeats was considered an independent unit for the subsequent analyses. The same dot-matrix analysis was repeated with *FLG-like* sequences from chimpanzee and crab-eating macaque, however we used chimpanzee-repeat 1 and macaque-repeat 1 as references, respectively.

### Multiple alignment and phylogenetic analyses

We performed the multiple sequence alignment using the profile alignment for nucleotide and codon in ClustalW implemented in MEGA 6.06 (Additional file 1: Table S2A and B) [38]. Sandilands et al. [27] previously described the nucleotide and amino acid sequences for each repeat in human. We used their sequence of human-repeat 1 as a reference to perform nucleotide and codon multiple alignments with the repeated region previously recognized by dot-matrix analysis and identified the repeats for each species. We then constructed maximum parsimony trees, neighbor-joining trees, and maximum likelihood trees. Maximum parsimony trees were constructed using partial deletions and 1000 bootstrap resampling values. Neighbor-joining trees were constructed by considering pairwise deletions, proportional nucleotide differences (*p*-distance), and 1000 bootstrap resampling. We compared 25 DNA/Protein models in MEGA 6.06 and the best-fit model was Tamura-Nei + Gamma Distributed with Invariant sites model with an AICc of 15797.8 (Additional file 1: Table S3). Maximum likelihood trees were constructed using the following predefined parameters: partial deletions, Tamura-Nei model with a gamma distribution and invariable sites, the nearest-neighbor-interchange heuristic method, and 1000 bootstrap resampling. A cutoff value for the condensed tree was set at 50% (Fig. 2; Additional file 1: Figure S3A and B, Figure S4 A, B and C).

### Estimation of polymorphic/variant sites, nucleotide diversity, and ratio of synonymous and nonsynonymous sites using the DNAsp5 program

*K*a/*K*s is the ratio of the number of nonsynonymous nucleotide substitutions per total number of nonsynonymous sites for each codon (*K*a), to the number of synonymous nucleotide substitutions per total number of synonymous sites for each codon (*K*s) [39, 40]. We estimated the *K*a/*K*s ratio for each pair of within-species repeats excluding gaps using the program DNAsp5. The level of purifying selection was then calculated as (1 − *K*a/*K*s) × 100.

Nucleotide diversity (*π*), the average number of nucleotide differences per site between sequences, was calculated using the DNA polymorphism option in DNAsp5 [40].

### Likelihood ratio tests for positive selection

Selection on the repeat region of *FLG* was evaluated using the codon substitution models (Codeml) tool in the PAML package (version 4.7). We compared several pairs of nested models with and without positive selection to be tested in a likelihood ratio framework to examine whether adaptive evolution had occurred [4].

We used the repeat sequences aligned to human-repeat 1 and performed three separate analyses: site-based test, branch-based test, and branch-site test. The site-based test compared models with neutral/negative selection (M1a and M7 (beta)) and positive selection (M2a and M8 (beta and *ω*)). The branch-based test considered heterogeneity among lineages and compared a free ratio model (where each branch has a different *ω*) with a one-ratio null model (M0, where *ω* is fixed to 1 for all branches). The branch-site test was used to evaluate whether the signature of positive selection was evident at a specific branch (foreground) against the rest of the branches (background) in the phylogeny. The Bayes Empirical Bayes approach was used to detect positive selection sites.

In all of the analyses, statistical comparisons of the models were made using likelihood ratio tests, in which twice the log-likelihood difference between the alternative and null models was compared with the critical values from a *χ* ^2^ distribution, with the number of degrees of freedom equal to the difference in the number of parameters between the two models. In the branch-site test, the Bonferroni correction was employed for the derived *P* value, to control for an increased type I error rate when comparing multiple foreground lineages [4, 24, 25, 41].

### Recombination and gene conversion events

We used a range of nonparametric methods in the RDP4 software package to characterize recombination and gene conversion events in the sequence alignments. Six phylogenetic and nucleotide substitution models (RDP, GENECONV, Bootscan, MaxChi, Chimaera, and SiScan) were implemented [32]. The general parameters were set to linear sequences at an acceptable *P* value of 0.01 after Bonferroni correction. We set phylogenetic evidence, polish breakpoints, and check for alignment consistency as prerequisites for the data processing. The RDP model was used with internal and external references. The G-scale mismatch penalty of GENECONV was changed to 2. Bootscan settings were modified to perform 1000 bootstrap replicates with a cutoff percentage of 95%. SiScan, Chimaera, and MaxChi were run using the default settings [42–46].

### Inference of gene duplication and loss in the species tree

The parsimony-based algorithm in the NOTUNG program reconciles the species tree that has the better fit for duplication, transfer, loss, and incomplete lineage sorting events from a set of gene trees. The maximum likelihood tree described above was used as the gene tree [28–30].

### Repeat duplication and divergence time of *FLG* repeats

Bayesian Evolutionary Analysis Sampling Trees is a cross-platform program for the Bayesian analysis of molecular sequences using Markov chain Monte Carlo to weight potential trees according to their posterior probability. The resulting tree has branch lengths that are proportional to divergence time [47–49]. We used high and low estimates derived from the dates based on the young and old calibration estimates. Crab-eating macaque repeats have previously been estimated to diverge between 16.3 and 20.8 Mya [50].

The parameters with the highest effective sample size were the site model TN93, estimation of substitution rate with gamma distribution = 4, log-normal relaxed clock, and birth-and-death model for all three nucleotide positions.

## Additional file

Additional file 1: Additional tables and figures. (PDF 3.32 kb)

## Abbreviations

FLG: Filaggrin

## References

[1] Nei M, Rooney A (2005). Concerted and birth-and-death evolution of multigene families. Annu Rev Genet.

[2] Austen R, Kobayashi T (2007). Highly efficient concerte evolution in the ribosomal DNA repeats: total rDNA repeat variation revealed by whole genome shotgun sequence data. Genome Res.

[3] Eirín-López J, Rebordinos L, Rooney A, Rozas J (2012). The birth-and-death evolution of multigene families revisited. Genome Dyn.

[4] Sabbagh A, Marin J, Vyssiére C, Lecompte E, Boukouvala S, Poloni ES, Darlu P, Crouau-Roy B (2013). Rapid birth-and-death evolution of the xenobiotic metabolizing NAT gene family in vertebrates with evidence of adaptive selection. BMC Evol Biol.

[5] Brown S, Irwin W (2012). One remarkable molecule: filaggrin. J Invest Dermatol.

[6] Kezic S, O’Regan G, Yau N, Sandilands A, Campbell L, Kroboth K, Watson R, Rowland M, McLean I (2011). Levels of filaggrin degradation products are influenced by both filaggrin genotype and atopic dermatitis severity. Allergy.

[7] Brown S, Kroboth K, Sandilands A, Campbell LE, Pohler E, Kezic S, Cordell H, McLean I, Irvine A (2012). Intragenic copy number variation within filaggrin contributes to the risk of atopic dermatitis with a dose-dependent effect. J Invest Dermatol.

[8] De D, Handa S (2012). Filaggrin mutations and the skin. Indian J Dermatol Venereol Leprol.

[9] Gan SQ, McBride W, Idler W, Markov N, Steinert P (1990). Organization, structure and polymorphisms of the human profilaggrin gene. Biochemistry.

[10] Redon R, Ishikawa S, Fitch K, Feuk L, Perry G, Andrews D, Fiegler H, Shapero M, Carson A, Chen W (2006). Global variation in copy number in the human genome. Nature.

[11] Smith FJ, Irvine AD, Terron-Kwiatkowski A, Sandilands A, Campbell LE, Zhao Y, Liao H, Evans AT, Goudie D, Lewis-Jone S (2006). Loss-of-function mutations in the gene encoding filaggrin cause ichthyosis vulgaris. Nat Genet.

[12] Fallon P, Sasaki T, Sandilands A, Campbell L, Saunders S, Mangan N, Callanan J, Kawasaki H, Shiohama A, Kubo A (2009). A homozygous frameshift mutation in the murine filaggrin gene facilitates enhanced percutaneous allergen priming. Nat Genet.

[13] Kanda S, Sasaki T, Shiohama A, Nishifuji K, Amagai M, Iwasaki T, Kudoh J (2013). Characterization of canine filaggrin: gene structure and protein expression in dog skin. Vet Dermatol.

[14] Feuk L, Carson AR, Scherer SW (2006). Structural variation in the human genome. Nat Rev Genet.

[15] Fondon JW, Garner HR (2004). Molecular origins of rapid and continuous morphological evolution. PNAS.

[16] Gazave E, Darre F, Morcillo-Suarez C, Petit-Marty N, Carreno A, Marigorta U, Ryder O, Blancher A, Rocchi M, Bosch E (2011). Copy number variation analysis in the great apes reveals species-specific patterns of structural variation. Genome Res.

[17] Paudel Y, Madsen O, Megens HJ, Frantz L, Bosse M, Bastiaansen J, Crooijmans R, Groenen M (2013). Evolutionary dynamics of copy number variation in pig genomes in the context of adaptation and domestication. BMC Genomics.

[18] Sudmant P, Huddleston J, Catacchio C, Malig M, Hillier L, Baker C, Mohajeri K, Kondova I, Bontrop R, Persengiev S (2013). Evolution and diversity of copy number variation in the great ape lineage. Genome Res.

[19] Koren S, Schatz M, Walenz B, Martin J, Howard J, Ganapathy G, Wang Z, Rasko D, McCombie R, Jarvis E (2012). Hybrid error correction and de novo assembly of single-molecule sequencing reads. Nat Biotechnol.

[20] Martin J, Wang Z (2011). Next-generation transcriptome assembly. Nat Genet.

[21] Nei M, Rogozin I, Piontkivska H (2000). Purifying selection and birth-and-death evolution in the ubiquitin gene family. Proc Natl Acad Sci.

[22] Nei M (2007). The new mutation theory of phenotypic evolution. PNAS.

[23] Sandilands A, Sutherland C, Irvine A, McLean H (2009). Filaggrin in the frontline: role in skin barrier function and disease. J Cell Sci.

[24] Yang Z (2007). PAML 4: phylogenetic analysis by maximum likelihood. Mol Biol Evol.

[25] Yang Z (1997). PAML: a program package for phylogenetic analysis by maximum likelihood. Comput Appl Biosci.

[26] Hoste E, Kemperman P, Devos M, Denecker G, Kezic S, Yau N, Gilbert B, Lippens S, De Groote P, Roelandt R (2011). Caspase-14 is required for filaggrin degradation to natural moisturizing factors in skin. J Invest Dermatol.

[27] Sandilands A, Terron-Kwiatkowski A, Hull P, O’Regan G, Clayton T, Watson R, Carrick T, Evans A, Liao H, Zhao Y (2007). Comprehensive analysis of the gene encoding filaggrin uncovers prevalent and rare mutations in ichthyosis vulgaris and atopic eczema. Nat Genet.

[28] Durand D, Halldórsson BV, Vernot B (2006). A hybrid micro-macroevolutionary approach to gene tree reconstruction. J Comput Biol.

[29] Stolzer M, Lai H, Xu M, Sathaye D, Vernot B, Durand D (2012). Inferring duplications, losses, transfers, and incomplete lineage sorting with non-binary species trees. Bioinformatics.

[30] Vernot B, Stolzer M, Goldman A, Durand D (2008). Reconciliation with non-binary species trees. J Comput Biol.

[31] Zhu L, Zhang Y, Hu Y, Wen T, Wang Q. Dynamic actin gene family evolution in primates. Biomed Res Int. 2013;2013:1–11. Article ID 630803. 10.1155/2013/630803 PMC3690210 23841080

[32] Martin D, Lemey P, Lott M, Moulton V, Posada D, Lefeuvre P (2010). RDP3: a flexible and fast computer program for analyzing recombination. Bioinformatics.

[33] Sawyer SA. GENECONV: a computer package for the statistical detectin of gene conversion. Distributed by the author, Department of Mathematics, Washington University in St. Louis. 1999. http://www.math.wustl.edu/~sawyer.

[34] Chen JM, Cooper D, Chuzhanova N, Férec C, Patrinos G (2007). Gene conversion: mechanisms, evolution and human disease. Nature.

[35] Caburet S, Cocquet J, Vaiman D, Veitia RA (2005). Coding repeats and evolutionary “agility”. BioEssays.

[36] Hosak L, Silhan P, Hosakova J (2012). Genomic copy number variations: a breakthrough in our knowledge on schizophrenia etiology?. Neuro Endocrinol Lett.

[37] Aldred P, Hollox E, Armour J (2005). Copy number polymorphism and expression level variation of the human α-defensin genes DEFA1 and DEFA3. Hum Mol Genet.

[38] Tamura K, Peterson D, Peterson N, Stecher G, Nei M, Kumar S (2011). MEGA5: molecular evolutionary genetics analysis using maximum likelihood, evolutionary distance, and maximum parsimony methods. Mol Biol Evol.

[39] Hu T, Banzhaf W (2008). Nonsynonymous to synonymous substitution ratio ka/ks: measurement for rate of evolution in evolutionary computation. R. Parallel Problem Solving from Nature - PPSN X.

[40] Librado P, Rozas J (2009). DnaSP v5:a software for comprehensive analysis of DNA polymorphism data. Bioinformatics.

[41] Anisimova M, Bielawski J, Yang Z (2001). Accuracy and power of the likelihood ratio test in detecting adaptive molecular evolution. Mol Biol Evol.

[42] Martin D, Rybicki E (2000). RDP: detection of recombination amongst aligned sequences. Bioinformatics.

[43] Martin D, Posada D, Crandall K, Williamson C (2005). A modified bootscan algorithm for automated identification of recombinant sequences and recombination breakpoints. A modified bootscan algorithm for automated identification of recombinant sequences and recombination breakpoints. AIDS Res Hum Retroviruses.

[44] Maynard-Smith J (1992). Analyzing the mosaic structure of genes. J Mol Evol.

[45] Padidam M, Sawyer S, Fauquet C (1999). Possible emergence of new geminiviruses by frequent recombination. Virology.

[46] Posada D, Crandall K (2001). Evaluation of methods for detecting recombination from DNA sequences: computer simulations. Proc Natl Acad Sci.

[47] Bouckaert R, Heled J, Kuhnert D, Vaughan T, Wu C, Xie D, Suchard MA, Rambaut A, Drummond AJ (2014). BEAST2: a software platform for bayesian evolutionary analysis. PLoS Comput Biol.

[48] Drummond A, Suchard M, Xie D, Rambaut A (2012). Bayesian phylogenetics with BEAUti and the BEAST 1.7. Mol Biol Evol.

[49] Hahn M, De Bie T, Stajich J, Nguyen C, Cristianini N (2005). Estimating the tempo and mode of gene family evolution from comparative genomic data. Genome Res.

[50] Steiper M, Young N (2006). Primate molecular divergence dates. ScienceDirect.

