# Supplementary material for: Structure and evolution of the filaggrin gene repeated region in primates
Source: BMC Evol Biol. 2017 Jan 11;17:10. doi: 10.1186/s12862-016-0851-5 (PMC5225520; doi:10.1186/s12862-016-0851-5)

Filaggrin-like gene evolution in chimpanzee and crab-eating macaque

Our study focuses on the evolution of the repeated region of filaggrin under the birth-and-death model. The birth-and-death model suggests the presence of pseudogenes. In the NCBI gene database we did not find any filaggrin pseudogenes but there are two types of genes that are similar to the complete repeats from filaggrin; filaggrin-2 and filaggrin-like. Filaggrin-2 repeats are shorter than complete filaggrin repeats (225bp) and we did not consider filaggrin-2 for further analysis. We found one filaggrin-like gene located in chromosome 1 of chimpanzee and two filaggrin-like genes located in chromosomes 1 and 3 of macaque. We compared these sequences with repeat of the corresponding species by a dot-matrix analysis using the Harrplot program (Supplementary Figure S2C, D and E, Supplementary Material Online). Filaggrin-like gene in chimpanzee includes 1 complete repeat between two incomplete repeats. Only one complete filaggrin repeat was found in the chimpanzee filaggrin-like sequence thus we could not perform any evolutionary analysis. Filaggrin-like gene in macaque located in chromosome 3 does not include any filaggrin repeats and the one located in chromosome 1 includes 5 complete repeats. In crab-eating macaque filaggrin-like and filaggrin have a similar total variation, average *K*a/*K*s, nucleotide variation; in addition no positively selected sites were detected (Supplementary Table S6, S7 and S8, Supplementary Material Online). We concluded that filaggrin and filaggrin-like repeats have been evolving under a similar birth-and-death model with strong selective constraints and none positively selected codons.

Table S1. Primer sets for the filaggrin gene repeat region in human, chimpanzee, gorilla, orangutan, crab-eating macaque, and human variation in repeated region.

REPEATED REGION	
**Human**	
**Forward**	5′ -CTTGTCATATGGCTAACTGGCTTTCAGAGA-3′
**Reverse**	5′ -ATTGTGGGACAGTGATTATGTTGGAGAAAA-3′
**Human variation in repeated region**	
**Forward**	5′ -GTGCAAGCAGAAAAACATATGACA -3′
**Reverse**	5′ -CCTGTTTCGTGATCTGCCTTTGACATGG -3′
**Chimpanzee**	
**Forward**	5′ -TGTTCACATATAACACCTGAATGAGGATGAGAC-3′
**Reverse**	5′ -GGATAATAGAGAAAGATGTGCTAGCCCTG-3′
**Gorilla**	
**Forward**	5′ -GTAACACCTGAATGAGGATGAGACGAAA-3′
**Reverse**	5′ -TAGCCCTGATGTTGATATAGCCACTTTG-3′
**Orangutan**	
**Forward**	5′ -AAGAAAAAGCGTCACTTACCCCATCAAA-3′
**Reverse**	5′ -TTGACATGGCTTAATCACCACCTAAGTT-3′
**Crab-eating macaque**	
**Forward**	5′ -ACACCTGAGCGAGGATGAGATGAAAAAG-3′
**Reverse**	5′ -TCTGGCCATGGGGAAGTATGTAATTTGG-3′

Table S2. (**A**) Nucleotide alignment of all complete repeat sequences to Human R1 performed by ClustalW. (**B)** Aminoacid alignment of all complete repeat sequences to Human R1 performed by ClustalW.

A.

Nucleotide # (bp	1	2	3	4	5	6	7	8	9	10	11	12	13	14	15	16	17	18	19	20	21	22	23	24	25
HumanR1	C	G	G	T	C	T	G	G	A	C	G	T	T	C	A	G	G	G	T	C	T	T	C	C	C
HumanR2	A	.	.	.	.	.	C	.	.	.	.	.	.	.	.	.	.	.	.	.	.	.	T	.	.
HumanR3	A	.	.	.	.	.	.	.	.	.	.	.	.	.	.	.	.	.	.	.	.	.	T	.	A
HumanR4	A	.	C	.	.	.	.	.	.	.	.	.	.	.	.	A	.	.	.	.	.	.	T	.	.
HumanR5	A	.	.	.	.	.	.	.	.	.	.	.	.	.	.	.	.	.	.	.	.	.	T	.	.
HumanR6	A	.	C	.	.	.	.	.	.	.	.	.	.	.	.	.	.	.	.	.	.	.	T	.	.
HumanR7	A	.	.	.	.	.	.	.	.	.	.	.	.	.	.	.	.	.	.	.	.	.	T	.	.
HumanR8	A	C	.	.	.	.	.	.	.	.	A	.	.	.	.	.	.	A	.	.	.	.	T	.	.
HumanR9	A	C	.	.	.	.	.	.	.	.	A	.	.	.	.	.	.	A	.	.	.	.	T	.	.
HumanR10	A	.	.	.	.	.	.	.	.	.	.	.	.	.	.	.	.	.	.	.	.	.	T	.	.
ChimpanzeeR1	.	.	.	.	.	.	.	.	.	.	A	.	.	.	.	.	.	.	.	.	.	.	T	.	.
ChimpanzeeR2	A	.	C	.	.	.	.	.	.	.	.	.	.	.	.	.	.	.	.	.	.	.	T	.	.
ChimpanzeeR3	A	.	.	.	.	.	.	.	.	.	.	.	.	.	.	.	.	.	.	.	.	.	T	.	.
ChimpanzeeR4	A	.	.	.	.	.	.	.	.	.	.	.	.	.	.	.	.	.	.	.	.	.	T	.	.
ChimpanzeeR5	A	.	C	.	.	.	.	.	.	.	.	.	.	.	.	.	.	.	.	.	.	.	T	.	.
ChimpanzeeR6	A	.	.	.	.	.	.	.	.	.	.	.	.	.	.	.	.	.	.	.	.	.	T	.	.
ChimpanzeeR7	A	.	.	.	.	.	.	.	.	.	.	.	.	.	.	.	.	.	.	.	.	.	T	.	.
ChimpanzeeR8	A	.	.	.	.	.	.	.	.	.	.	.	.	.	.	.	.	A	.	.	.	.	T	.	.
ChimpanzeeR9	A	.	.	.	.	.	.	.	.	.	.	.	.	.	.	.	.	.	.	.	.	.	T	.	.
ChimpanzeeR10	A	.	C	.	.	.	.	.	.	.	.	.	.	.	.	.	.	.	.	.	.	.	T	.	.
GorillaR1	A	.	.	.	.	.	.	.	.	.	A	.	.	.	.	.	.	.	.	.	.	.	T	.	.
GorillaR2	A	.	.	.	.	.	.	.	.	.	.	.	.	.	.	.	.	.	.	.	.	.	T	.	.
GorillaR3	A	.	.	.	.	.	.	.	.	.	.	.	.	.	.	.	.	.	.	.	.	.	T	.	.
GorillaR4	A	.	.	.	.	.	.	.	.	.	.	.	.	.	.	.	.	.	.	.	.	.	T	.	.
GorillaR5	A	.	.	.	.	.	.	.	.	.	.	.	.	.	.	.	.	.	.	.	.	.	T	.	.
GorillaR6	A	.	.	.	.	.	.	.	.	.	.	.	.	.	.	.	.	.	.	.	.	.	T	.	.
GorillaR7	A	.	.	.	.	.	.	.	.	.	.	.	.	.	.	.	.	.	.	.	.	.	T	.	.
GorillaR8	A	.	.	.	.	.	.	.	.	.	.	.	.	.	.	.	.	.	.	.	.	.	T	.	.
GorillaR9	A	.	.	.	.	.	.	.	.	.	.	.	.	.	.	.	.	.	.	.	.	.	T	.	.
GorillaR10	A	.	.	.	.	.	.	.	.	.	.	.	.	.	.	.	.	.	.	.	.	.	T	.	.
OrangutanR1	A	.	.	.	.	.	.	.	.	.	A	.	.	.	.	.	.	.	.	.	.	.	T	.	.
OrangutanR2	A	.	.	.	.	.	.	.	.	.	.	.	.	.	.	.	.	.	.	.	.	.	T	.	.
OrangutanR3	T	.	.	.	.	.	.	.	.	.	.	.	.	.	.	.	.	.	.	.	.	.	T	.	.
OrangutanR4	A	.	C	.	.	.	.	.	.	.	.	.	.	.	.	.	.	.	.	.	.	.	T	.	.
OrangutanR5	A	.	C	.	.	.	.	.	.	.	A	.	.	.	.	.	.	.	.	.	.	.	T	.	.
OrangutanR6	A	.	.	.	.	.	.	.	.	.	.	.	.	.	.	.	.	.	.	.	.	.	T	.	.
OrangutanR7	A	.	.	.	.	.	.	.	.	.	.	.	.	.	.	.	.	.	.	.	.	.	T	.	.
OrangutanR8	A	.	.	.	.	.	.	.	.	.	.	.	A	.	.	.	.	.	.	.	.	.	T	.	.
OrangutanR9	A	A	.	.	.	.	.	.	.	.	A	.	.	.	.	.	.	.	.	.	.	.	T	.	.
MacaqueR12	A	.	.	.	.	.	.	.	.	.	.	.	.	.	.	.	.	.	.	.	.	.	T	.	.
MacaqueR1	A	.	.	.	.	.	.	.	.	.	.	.	.	.	.	.	.	.	.	.	.	.	T	.	.
MacaqueR2	A	.	.	.	.	.	.	.	.	.	.	.	.	.	.	.	.	.	.	.	.	.	T	.	.
MacaqueR3	A	.	.	.	.	.	.	.	.	.	.	.	.	.	.	.	.	.	.	.	.	.	T	.	G
MacaqueR4	A	.	.	.	.	.	.	.	.	.	.	.	.	.	.	.	.	.	.	.	.	.	T	.	.
MacaqueR5	A	.	.	.	.	.	.	.	.	.	.	.	.	.	.	.	.	.	.	.	.	.	T	.	G
MacaqueR6	A	.	.	.	.	.	.	.	.	.	.	.	.	.	.	.	.	.	.	.	.	.	T	.	.
MacaqueR7	A	.	.	.	.	.	.	.	.	.	.	.	.	.	.	.	.	.	.	.	.	.	T	.	G
MacaqueR8	A	.	.	.	.	.	.	.	.	.	.	.	.	.	.	.	.	.	.	.	.	.	T	.	.
MacaqueR9	A	.	.	.	.	.	.	.	.	.	.	.	.	.	.	.	.	.	.	.	.	.	T	.	G
MacaqueR10	A	.	.	.	.	.	.	.	.	.	.	.	.	.	.	.	.	.	.	.	.	.	T	.	.
MacaqueR11	A	.	.	.	.	.	.	.	.	.	.	.	.	.	.	.	.	.	.	.	.	.	T	.	G

Nucleotide # (bp	26	27	28	29	30	31	32	33	34	35	36	37	38	39	40	41	42	43	44	45	46	47	48	49	50
HumanR1	T	C	T	A	C	C	A	G	G	T	G	A	G	C	A	C	T	C	A	T	G	A	A	C	A
HumanR2	.	.	.	.	.	.	.	.	.	.	.	.	.	.	.	.	.	.	.	.	A	.	.	.	.
HumanR3	.	.	.	.	.	.	.	.	.	.	.	.	.	.	.	.	.	.	.	.	.	.	.	.	.
HumanR4	.	.	.	.	.	.	.	.	.	.	.	.	.	.	T	.	.	.	.	.	.	.	.	.	.
HumanR5	.	.	.	.	.	.	.	.	.	.	.	.	.	.	.	.	.	.	.	.	.	.	.	.	.
HumanR6	.	.	.	.	.	.	.	.	.	.	.	.	.	.	.	.	.	.	.	.	.	.	.	.	.
HumanR7	.	.	.	.	.	.	.	.	.	.	.	.	.	.	.	.	.	.	.	.	.	.	.	.	.
HumanR8	.	.	.	.	.	.	.	.	.	.	.	.	.	.	.	.	.	.	.	.	.	.	.	.	.
HumanR9	.	.	.	.	.	.	.	.	.	.	.	.	.	.	.	.	.	.	.	.	.	.	.	.	.
HumanR10	.	.	.	.	.	.	.	.	.	.	.	.	.	.	.	.	.	.	.	.	.	.	.	.	.
ChimpanzeeR1	.	.	.	.	.	.	.	.	.	.	.	.	.	.	.	.	.	.	.	.	.	.	.	.	.
ChimpanzeeR2	.	.	.	.	.	.	.	.	.	.	.	.	.	.	T	.	.	.	.	.	.	.	.	.	.
ChimpanzeeR3	.	.	.	.	.	.	.	.	.	.	.	.	.	.	.	.	.	.	.	.	.	.	.	.	.
ChimpanzeeR4	.	.	.	.	.	.	.	.	.	.	.	.	.	.	.	.	.	.	.	.	.	.	.	.	.
ChimpanzeeR5	.	.	.	.	.	.	.	.	.	.	.	.	.	.	T	.	.	.	.	.	.	.	.	.	.
ChimpanzeeR6	.	.	.	.	.	.	.	.	.	.	.	.	.	.	.	.	.	.	.	.	.	.	.	.	.
ChimpanzeeR7	.	.	.	.	.	.	.	.	.	.	.	.	.	.	.	.	.	.	.	.	.	.	.	.	.
ChimpanzeeR8	.	.	.	.	.	.	.	.	.	.	.	.	.	.	.	.	.	.	.	.	.	.	.	.	.
ChimpanzeeR9	.	.	.	.	.	.	.	.	.	.	.	.	.	.	.	.	.	.	.	.	.	.	.	.	.
ChimpanzeeR10	.	.	.	.	.	.	.	.	.	.	.	.	.	.	.	.	.	.	.	.	.	.	.	.	.
GorillaR1	.	.	.	.	.	.	.	.	.	.	.	.	.	.	.	.	.	.	.	.	.	.	.	.	.
GorillaR2	G	.	.	.	.	.	.	.	.	.	.	.	.	.	.	.	.	.	.	.	.	.	.	.	.
GorillaR3	.	.	.	.	.	.	.	.	.	.	.	.	.	.	T	.	.	.	.	.	.	.	.	.	.
GorillaR4	.	.	.	.	.	.	.	.	.	.	.	.	.	.	.	.	.	.	.	.	.	.	.	.	G
GorillaR5	.	.	.	.	.	.	.	.	.	.	.	.	.	.	T	.	.	.	.	.	.	.	.	.	.
GorillaR6	.	.	.	.	.	.	.	.	.	.	.	.	.	.	.	.	.	.	.	.	.	.	.	.	G
GorillaR7	.	.	.	.	.	.	.	.	.	.	.	.	.	.	T	.	.	.	.	.	.	.	.	.	.
GorillaR8	.	.	.	.	.	.	.	.	.	.	.	.	.	.	.	.	.	.	.	.	C	.	.	.	.
GorillaR9	.	.	.	.	.	.	.	.	.	.	.	.	.	.	T	.	.	.	.	.	.	.	.	.	.
GorillaR10	.	.	.	.	.	.	.	.	.	.	.	.	.	.	.	.	.	.	.	.	.	.	.	.	.
OrangutanR1	C	.	.	.	.	.	.	.	.	.	.	.	.	.	.	.	.	.	.	.	.	.	.	.	.
OrangutanR2	C	.	C	.	.	.	.	.	.	.	.	G	A	.	.	.	.	.	.	.	.	.	.	.	.
OrangutanR3	C	.	.	.	.	.	.	.	.	.	.	.	.	.	.	.	.	.	.	.	A	.	.	.	.
OrangutanR4	C	.	.	.	.	.	.	.	.	.	.	.	.	.	.	.	.	.	.	.	.	.	.	.	.
OrangutanR5	C	.	.	.	.	.	.	T	.	.	.	.	.	.	.	.	.	.	.	.	.	.	.	.	.
OrangutanR6	.	.	.	.	.	.	.	.	.	.	.	.	.	.	.	.	.	.	.	.	.	.	.	.	.
OrangutanR7	C	.	.	.	.	.	.	.	.	.	.	.	.	.	.	.	.	.	.	.	.	.	.	.	.
OrangutanR8	C	.	.	.	.	.	.	.	.	.	.	.	.	.	.	.	.	.	.	.	.	.	.	.	.
OrangutanR9	.	.	.	.	.	.	.	.	.	.	.	.	.	.	.	.	.	.	.	C	.	.	.	.	.
MacaqueR12	.	.	.	.	.	.	.	.	.	.	.	.	.	.	.	.	.	.	.	C	.	.	.	.	.
MacaqueR1	.	.	.	.	.	.	.	.	.	.	.	.	.	.	.	.	.	.	.	C	.	.	.	.	.
MacaqueR2	.	.	.	.	.	.	.	.	.	.	.	.	.	.	.	.	.	.	.	C	.	.	.	.	.
MacaqueR3	.	.	.	.	.	.	.	.	.	.	.	.	.	.	.	.	.	.	.	.	.	.	.	.	.
MacaqueR4	.	.	.	.	.	.	.	.	.	.	.	.	.	.	.	.	.	.	.	C	.	.	.	.	.
MacaqueR5	.	.	.	.	.	.	.	.	.	.	.	.	.	.	.	.	.	.	.	.	.	.	.	.	.
MacaqueR6	.	.	.	.	.	.	.	.	.	.	.	.	.	.	.	.	.	.	.	C	.	.	.	.	.
MacaqueR7	.	.	.	.	.	.	.	.	.	.	.	.	.	.	.	.	.	.	.	.	.	.	.	.	.
MacaqueR8	.	.	.	.	.	.	.	.	.	.	.	.	.	.	.	.	.	.	.	C	.	.	.	.	.
MacaqueR9	.	.	.	.	.	.	.	.	.	.	.	.	.	.	.	.	.	.	.	.	.	.	.	.	.
MacaqueR10	.	.	.	.	.	.	.	.	.	.	.	.	.	.	.	.	.	.	.	C	.	.	.	.	.
MacaqueR11	.	.	.	.	.	.	.	.	.	.	.	.	.	.	.	.	.	.	.	.	.	.	.	.	.

Nucleotide # (bp	51	52	53	54	55	56	57	58	59	60	61	62	63	64	65	66	67	68	69	70	71	72	73	74	75
HumanR1	G	C	C	T	G	A	C	T	C	T	G	C	C	C	A	T	G	G	A	C	G	G	A	C	C
HumanR2	.	T	.	.	.	.	G	.	.	C	T	.	.	.	.	.	.	.	.	T	.	.	.	.	A
HumanR3	.	T	.	.	.	.	G	.	.	.	.	.	.	.	.	.	.	.	G	.	.	.	.	.	.
HumanR4	.	T	.	.	.	.	G	.	.	C	A	.	A	.	.	C	.	.	.	.	A	.	.	.	T
HumanR5	.	T	.	.	.	.	G	.	.	C	.	.	.	.	.	.	.	.	.	.	.	C	.	.	A
HumanR6	.	T	.	.	.	.	G	.	.	C	A	.	.	.	.	.	.	.	.	.	A	.	T	.	T
HumanR7	.	T	.	.	.	.	G	.	.	C	.	.	.	.	.	.	.	.	.	.	.	.	.	.	.
HumanR8	.	T	.	.	.	.	G	.	.	C	T	.	.	.	.	.	.	.	.	T	.	.	.	.	G
HumanR9	.	T	.	.	.	.	G	.	.	C	T	.	.	.	.	.	.	.	.	T	.	.	.	.	G
HumanR10	.	T	.	.	.	.	G	.	.	.	.	.	.	.	.	.	.	.	G	.	.	.	.	.	.
ChimpanzeeR1	.	T	.	.	.	.	.	.	.	.	.	.	.	.	.	.	.	.	G	.	.	.	.	.	.
ChimpanzeeR2	.	T	.	.	.	.	G	.	.	C	A	.	A	T	.	C	.	.	.	.	A	.	.	.	T
ChimpanzeeR3	.	T	.	.	.	.	G	.	.	C	.	.	.	.	.	.	.	.	.	.	.	.	.	.	A
ChimpanzeeR4	.	T	.	.	.	.	G	.	.	.	.	.	.	.	.	.	.	.	G	.	.	.	.	.	.
ChimpanzeeR5	.	T	.	.	.	.	G	.	.	C	A	.	A	T	.	C	.	.	.	.	A	.	.	.	T
ChimpanzeeR6	.	T	.	.	.	.	G	.	.	C	.	.	.	.	.	.	.	.	.	.	.	.	.	.	A
ChimpanzeeR7	.	T	.	.	.	.	G	.	.	.	.	.	.	.	.	.	.	.	.	.	.	.	.	.	.
ChimpanzeeR8	.	T	.	.	.	.	A	.	.	C	T	.	.	.	.	.	.	.	.	T	.	.	.	.	A
ChimpanzeeR9	.	T	.	.	.	.	G	.	.	C	A	.	.	.	.	.	.	.	G	.	.	.	.	.	.
ChimpanzeeR10	.	.	.	.	.	.	G	.	.	C	A	.	.	.	.	.	.	.	.	.	A	.	T	.	T
GorillaR1	.	T	.	.	.	.	G	.	.	.	.	.	.	.	.	.	.	.	G	.	.	.	.	.	.
GorillaR2	.	T	.	.	.	.	G	.	A	C	T	.	.	.	.	.	.	.	.	T	.	.	.	.	A
GorillaR3	.	T	.	.	.	.	G	.	.	C	.	T	.	.	.	.	.	.	.	.	A	.	.	.	.
GorillaR4	.	T	.	.	.	.	G	.	.	.	.	.	.	.	.	.	.	.	G	.	.	.	.	.	.
GorillaR5	.	T	.	.	.	.	G	.	.	C	.	T	.	.	.	.	.	.	.	.	A	.	.	.	.
GorillaR6	.	T	.	.	.	.	G	.	.	.	.	.	.	.	.	.	.	.	G	.	.	.	.	.	.
GorillaR7	.	T	.	.	.	.	G	.	.	C	.	T	.	.	.	.	.	.	.	.	A	.	.	.	.
GorillaR8	.	T	.	.	.	.	G	.	.	C	T	.	.	.	.	.	.	.	.	T	.	.	.	.	A
GorillaR9	.	T	.	.	.	.	G	.	.	C	.	.	.	.	.	.	.	.	.	.	A	.	.	.	.
GorillaR10	.	T	.	.	.	.	G	.	.	C	.	.	.	.	.	.	.	.	.	.	A	.	.	.	G
OrangutanR1	.	T	.	.	.	.	G	.	A	C	.	.	.	.	.	.	.	.	.	.	A	.	C	.	T
OrangutanR2	.	T	.	.	.	.	G	.	.	.	.	.	.	.	.	.	.	.	.	.	.	.	.	.	.
OrangutanR3	.	T	.	.	.	.	G	.	.	C	A	.	.	.	.	.	.	.	.	.	.	.	.	.	.
OrangutanR4	.	T	.	.	.	.	G	.	.	C	.	.	.	.	.	.	.	.	.	.	.	.	.	.	T
OrangutanR5	.	T	.	.	.	.	G	.	.	C	.	T	.	.	.	.	.	.	.	.	.	.	.	.	.
OrangutanR6	.	T	.	.	.	.	G	.	.	C	.	T	.	.	.	.	.	C	.	.	.	.	.	.	T
OrangutanR7	.	T	.	.	.	.	G	.	.	C	.	T	.	.	.	.	.	.	.	.	.	.	.	.	.
OrangutanR8	.	T	.	.	.	.	G	.	.	C	.	.	.	.	.	.	.	.	.	.	A	.	.	.	.
OrangutanR9	.	T	.	.	.	.	G	.	.	C	A	.	.	.	.	.	.	.	.	.	.	.	.	.	.
MacaqueR12	.	T	.	.	.	.	G	.	.	C	A	.	.	.	.	.	.	.	.	.	.	.	.	.	T
MacaqueR1	.	T	.	.	.	.	G	.	.	C	A	.	.	.	.	.	.	.	.	.	.	.	.	.	T
MacaqueR2	.	T	.	.	.	.	G	.	.	C	A	.	.	.	.	.	.	.	.	.	.	.	.	.	T
MacaqueR3	.	T	.	.	.	.	G	.	.	C	A	.	.	.	.	.	.	.	.	.	.	.	.	.	T
MacaqueR4	.	T	.	.	.	.	G	.	.	C	A	.	.	.	.	.	.	.	.	.	.	.	.	.	T
MacaqueR5	.	T	.	.	.	.	G	.	.	C	A	.	.	.	.	.	.	.	.	.	.	.	.	.	T
MacaqueR6	.	T	.	.	.	.	G	.	.	C	A	.	.	.	.	.	.	.	.	.	.	.	.	.	T
MacaqueR7	.	T	.	.	.	.	G	.	.	C	A	.	.	.	.	.	.	.	.	.	.	.	.	.	T
MacaqueR8	.	T	.	.	.	.	G	.	.	C	A	.	.	.	.	.	.	.	.	.	.	.	.	.	T
MacaqueR9	.	T	.	.	.	.	G	.	.	C	A	.	.	.	.	.	.	.	.	.	.	.	.	.	T
MacaqueR10	.	T	.	.	.	.	G	.	.	C	A	.	.	.	.	.	.	.	.	.	.	.	.	.	T
MacaqueR11	.	T	.	.	.	.	G	.	.	C	A	.	.	.	.	.	.	.	.	.	.	.	.	.	T

Nucleotide # (bp	76	77	78	79	80	81	82	83	84	85	86	87	88	89	90	91	92	93	94	95	96	97	98	99	100
HumanR1	G	G	G	A	C	C	A	G	C	A	C	T	G	G	A	G	G	A	A	G	A	C	A	A	G
HumanR2	.	.	.	C	.	.	.	.	.	.	.	.	.	.	.	.	T	.	.	.	.	.	.	.	.
HumanR3	A	.	.	.	.	.	.	.	.	.	.	.	.	.	.	C	.	.	.	.	.	.	.	.	.
HumanR4	.	C	A	C	.	.	.	.	.	.	.	.	.	.	.	.	.	.	.	.	.	.	.	.	.
HumanR5	.	.	.	C	.	.	.	.	.	.	.	.	.	.	.	.	.	.	.	.	.	.	.	.	A
HumanR6	.	C	.	C	.	.	.	.	.	.	.	.	.	.	.	.	.	.	.	.	.	.	.	.	.
HumanR7	.	.	.	.	.	.	.	.	.	.	.	.	.	.	.	.	.	.	.	.	.	.	.	.	.
HumanR8	.	.	.	C	.	.	.	.	.	.	.	.	A	.	.	.	.	.	.	.	.	.	.	.	.
HumanR9	.	.	.	C	.	.	.	.	.	.	.	.	A	.	.	.	.	.	.	.	.	.	.	.	.
HumanR10	A	.	.	.	.	.	.	.	.	.	.	.	.	.	.	C	.	.	.	.	.	.	.	.	.
ChimpanzeeR1	.	.	.	.	.	.	.	.	.	.	.	.	.	.	.	.	.	.	.	.	.	.	.	.	.
ChimpanzeeR2	.	C	A	C	.	.	.	.	.	.	.	.	.	.	.	.	.	.	.	.	.	.	.	.	.
ChimpanzeeR3	.	.	.	C	.	.	.	.	.	.	.	.	.	.	.	.	.	.	.	.	.	.	.	.	A
ChimpanzeeR4	A	C	.	.	.	.	.	.	.	.	.	.	.	.	.	C	.	.	.	.	.	.	.	.	.
ChimpanzeeR5	.	C	A	C	.	.	.	.	.	.	.	.	.	.	.	.	.	.	.	.	.	.	.	.	.
ChimpanzeeR6	.	.	.	C	.	.	.	.	.	.	.	.	.	.	.	.	.	.	.	.	.	.	.	.	A
ChimpanzeeR7	.	.	.	.	.	.	.	.	.	.	.	.	.	.	.	.	.	.	.	.	.	A	.	.	A
ChimpanzeeR8	.	.	.	C	.	.	.	.	.	.	.	.	A	.	.	.	.	.	.	.	.	.	.	.	.
ChimpanzeeR9	.	.	T	.	.	.	.	.	.	.	.	.	.	.	.	.	.	.	.	.	.	.	.	.	.
ChimpanzeeR10	.	C	.	C	.	.	.	.	.	.	.	.	.	.	.	.	.	.	.	.	.	.	.	.	.
GorillaR1	.	.	T	.	.	.	.	.	.	.	.	.	.	.	.	.	.	.	.	.	.	.	.	.	A
GorillaR2	.	A	.	C	.	.	.	.	.	.	.	C	A	.	.	.	.	.	.	.	.	.	.	.	.
GorillaR3	.	.	.	.	.	.	.	.	.	.	.	.	.	.	.	.	.	.	.	.	.	.	.	.	A
GorillaR4	A	.	T	.	.	.	.	.	.	.	.	.	.	.	.	.	.	.	.	.	.	.	.	.	.
GorillaR5	.	.	.	.	.	.	.	.	.	.	.	.	.	.	.	.	.	.	.	.	.	.	.	.	A
GorillaR6	.	.	T	.	.	.	.	.	.	.	.	.	.	.	.	.	.	.	.	.	.	.	.	.	.
GorillaR7	A	.	.	.	.	.	.	.	.	.	.	.	.	.	.	.	.	.	.	.	.	.	.	.	A
GorillaR8	.	.	.	C	.	.	.	.	.	.	.	C	A	.	.	.	.	.	.	.	.	.	.	.	.
GorillaR9	.	.	.	.	.	.	.	.	.	.	.	.	.	.	.	.	.	.	.	.	.	.	.	.	A
GorillaR10	.	.	T	.	.	.	.	.	.	.	.	.	.	.	.	.	.	.	.	.	.	.	.	.	.
OrangutanR1	.	.	.	C	.	.	.	.	G	.	.	.	.	.	T	.	.	.	.	.	.	.	.	.	.
OrangutanR2	.	.	.	C	.	.	.	.	.	.	.	.	.	.	.	.	.	.	.	.	.	.	.	.	.
OrangutanR3	A	.	.	C	.	G	.	.	.	.	.	G	.	.	.	.	.	.	.	.	.	.	.	.	.
OrangutanR4	.	.	.	C	.	.	.	.	.	.	.	.	.	.	.	.	.	.	.	.	.	.	.	.	.
OrangutanR5	C	A	T	C	.	.	.	.	.	.	.	.	.	.	.	.	.	.	.	.	.	.	.	.	.
OrangutanR6	.	.	A	C	.	.	.	.	.	.	.	.	.	T	.	.	.	.	.	.	.	.	.	.	.
OrangutanR7	C	A	T	C	.	.	.	.	.	.	.	.	.	.	.	.	.	.	.	.	.	.	.	.	.
OrangutanR8	.	.	.	C	.	.	.	.	.	.	.	G	.	.	.	.	.	.	.	.	.	.	.	.	.
OrangutanR9	A	.	.	C	.	.	.	.	.	.	.	G	.	.	.	.	.	.	.	.	.	.	.	.	.
MacaqueR12	.	.	A	C	.	.	.	.	.	.	G	.	.	C	.	.	.	.	.	.	.	.	.	.	.
MacaqueR1	.	.	A	C	.	.	.	.	.	.	G	.	.	C	.	.	.	.	.	.	.	.	.	.	.
MacaqueR2	.	.	A	C	.	.	.	.	.	.	G	.	.	C	.	.	.	.	.	.	.	.	.	.	.
MacaqueR3	.	.	.	C	.	.	.	.	.	.	.	.	.	.	T	.	.	.	.	.	.	.	.	.	.
MacaqueR4	.	.	A	C	.	.	.	.	.	.	G	.	.	C	.	.	.	.	.	.	.	.	.	.	.
MacaqueR5	.	.	.	C	.	.	.	.	.	.	.	.	.	.	T	.	.	.	.	.	.	.	.	.	.
MacaqueR6	.	.	A	C	.	.	.	.	.	.	G	.	.	C	.	.	.	.	.	.	.	.	.	.	.
MacaqueR7	.	.	.	C	.	.	.	.	.	.	.	.	.	.	T	.	.	.	.	.	.	.	.	.	.
MacaqueR8	.	.	A	C	.	.	.	.	.	.	G	.	.	C	.	.	.	.	.	.	.	.	.	.	.
MacaqueR9	.	.	.	C	.	.	.	.	.	.	.	.	.	.	T	.	.	.	.	.	.	.	.	.	.
MacaqueR10	.	.	A	C	.	.	.	.	.	.	G	.	.	C	.	.	.	.	.	.	.	.	.	.	.
MacaqueR11	.	.	.	C	.	.	.	.	.	.	.	.	.	.	T	.	.	.	.	.	.	.	.	.	.

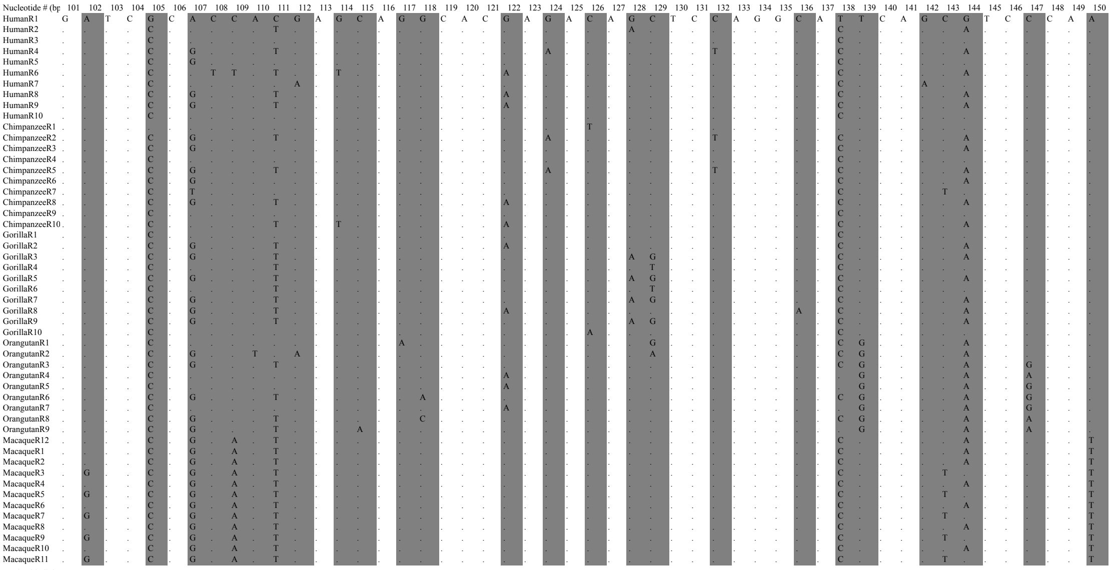

1

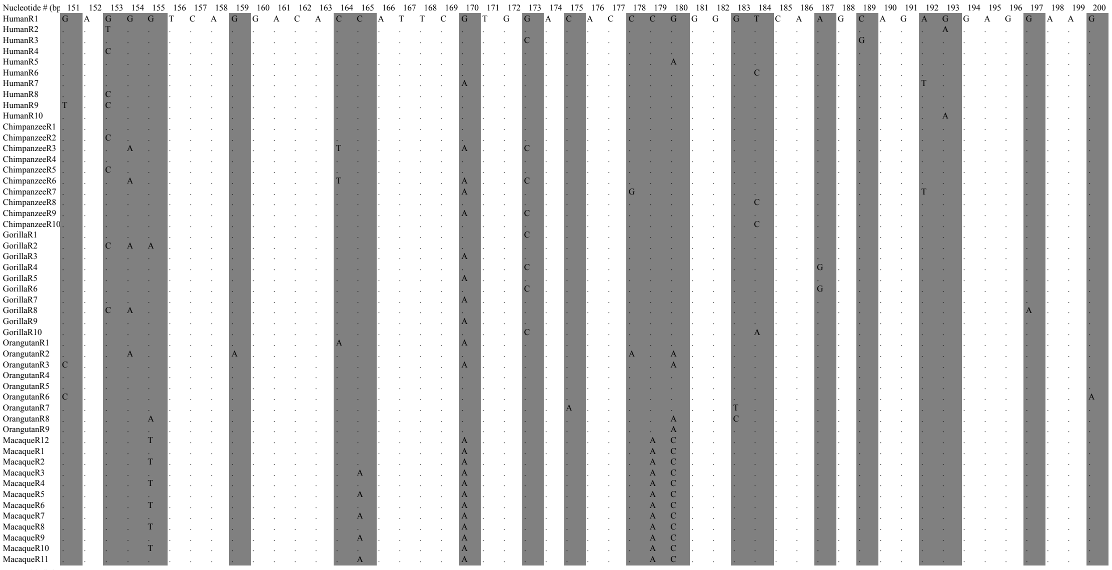

2

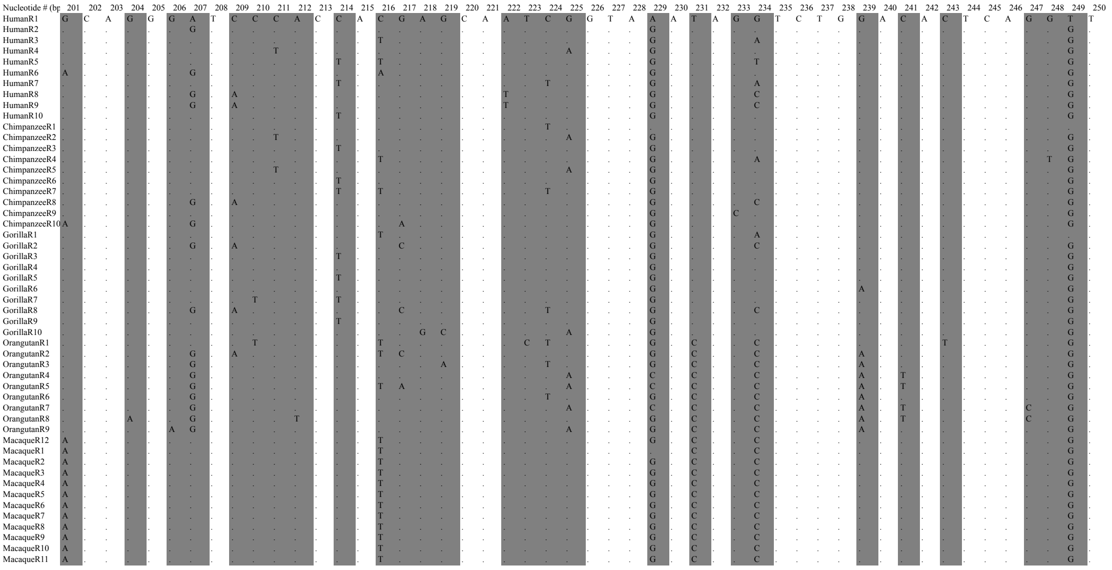

3

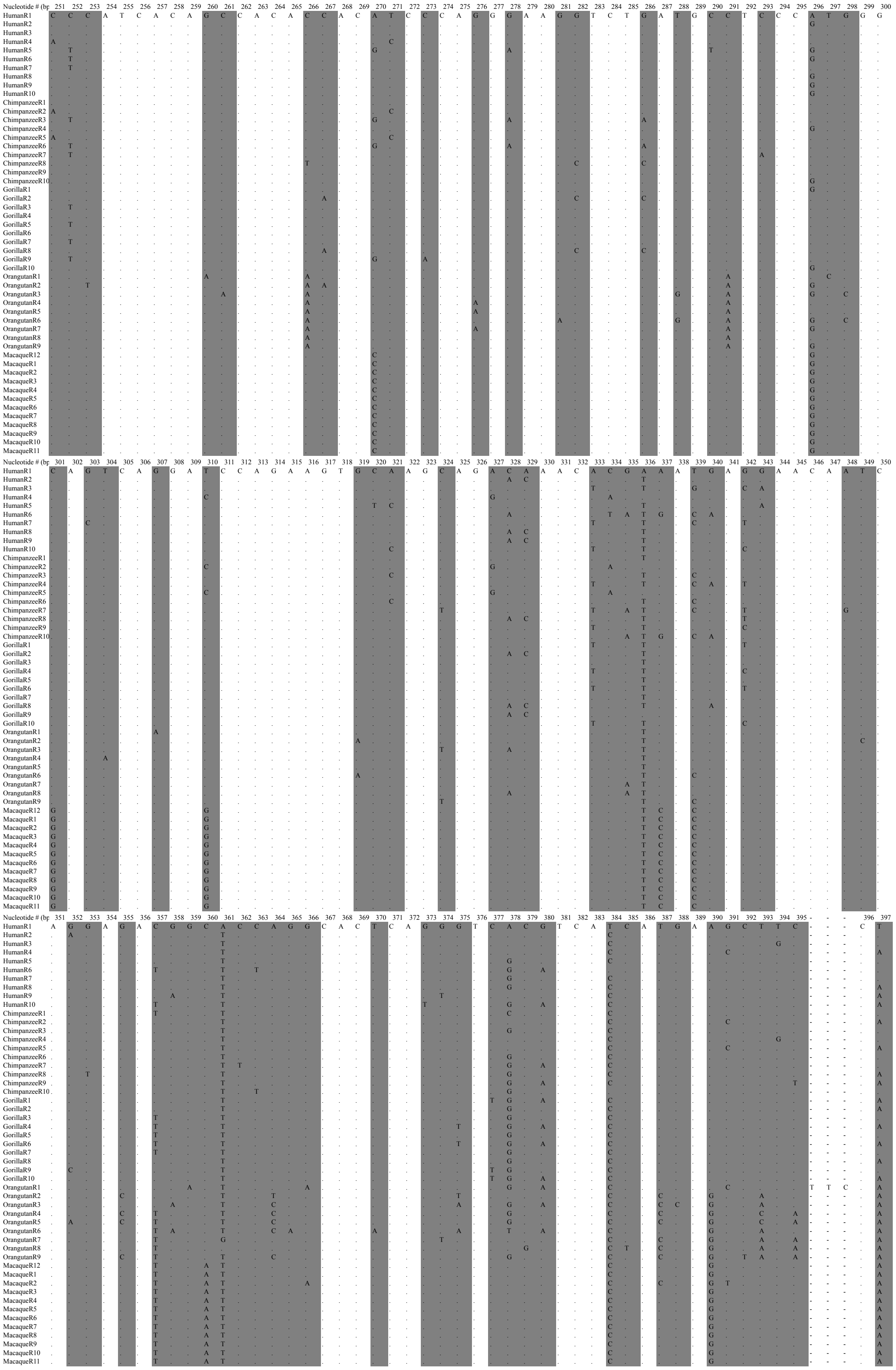

1

2

3

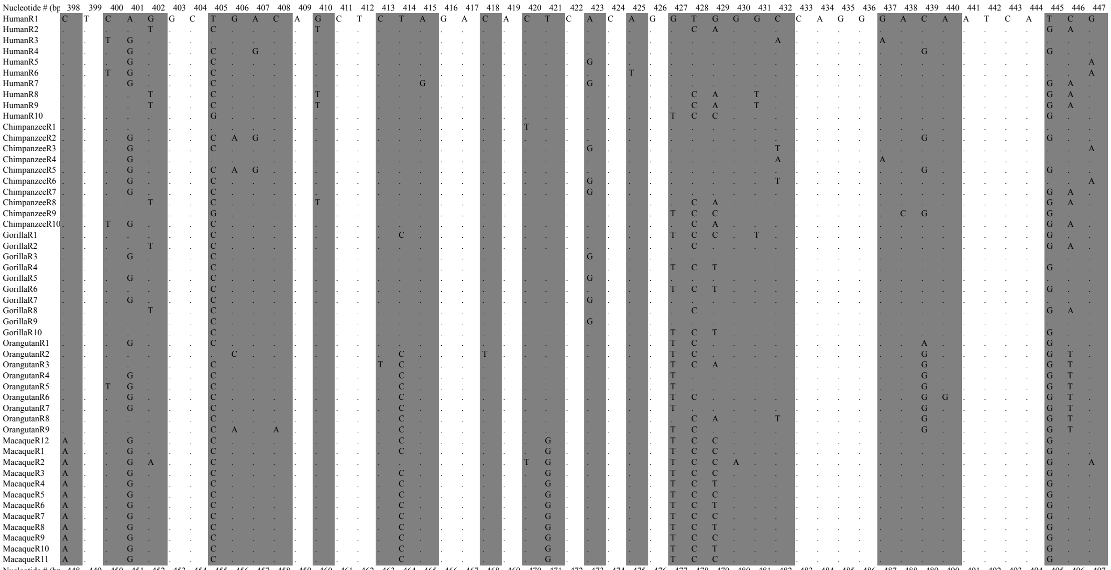

1

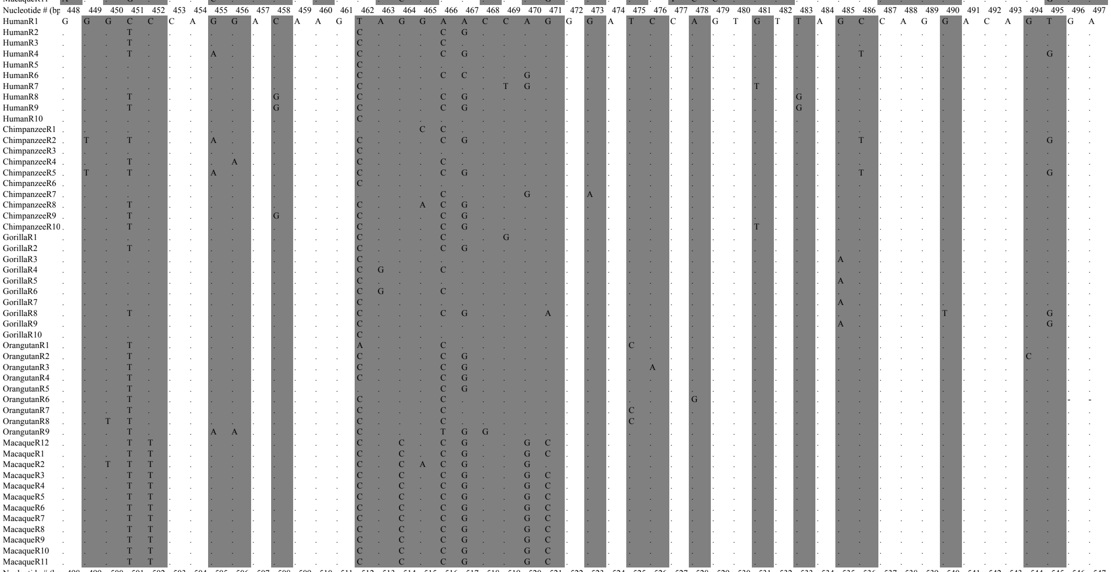

2

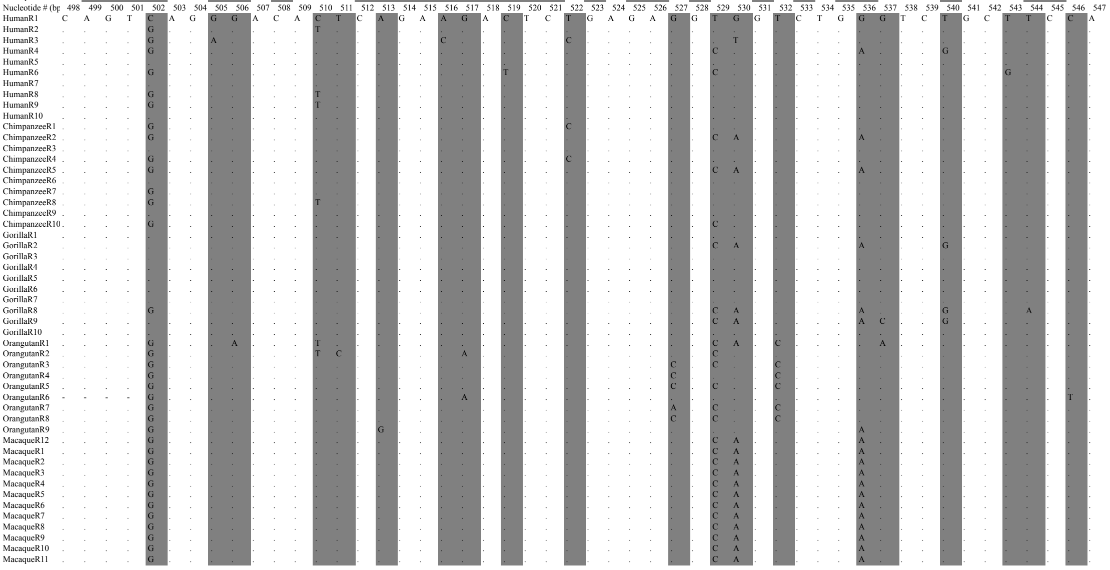

3

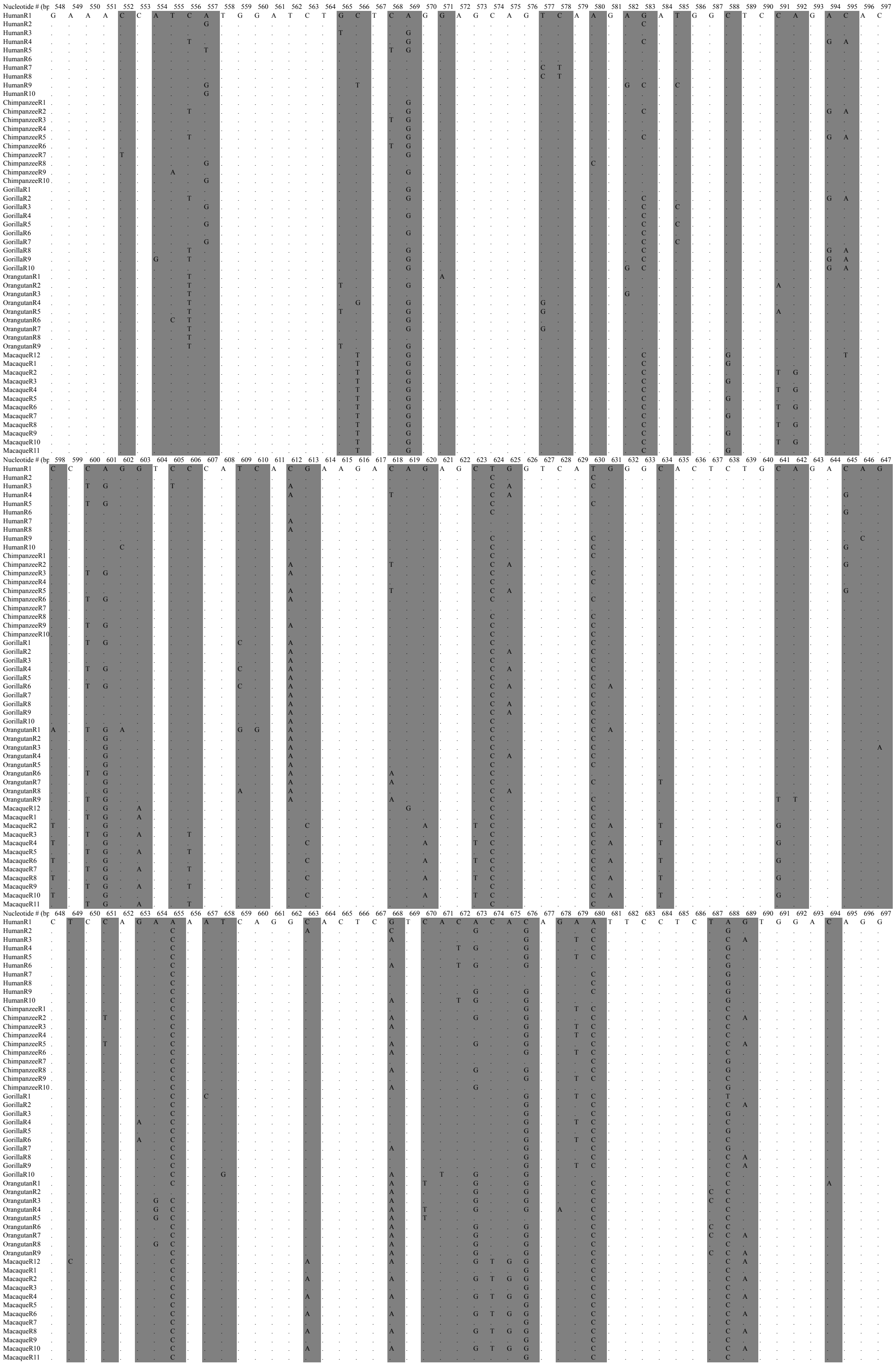

1

2

3

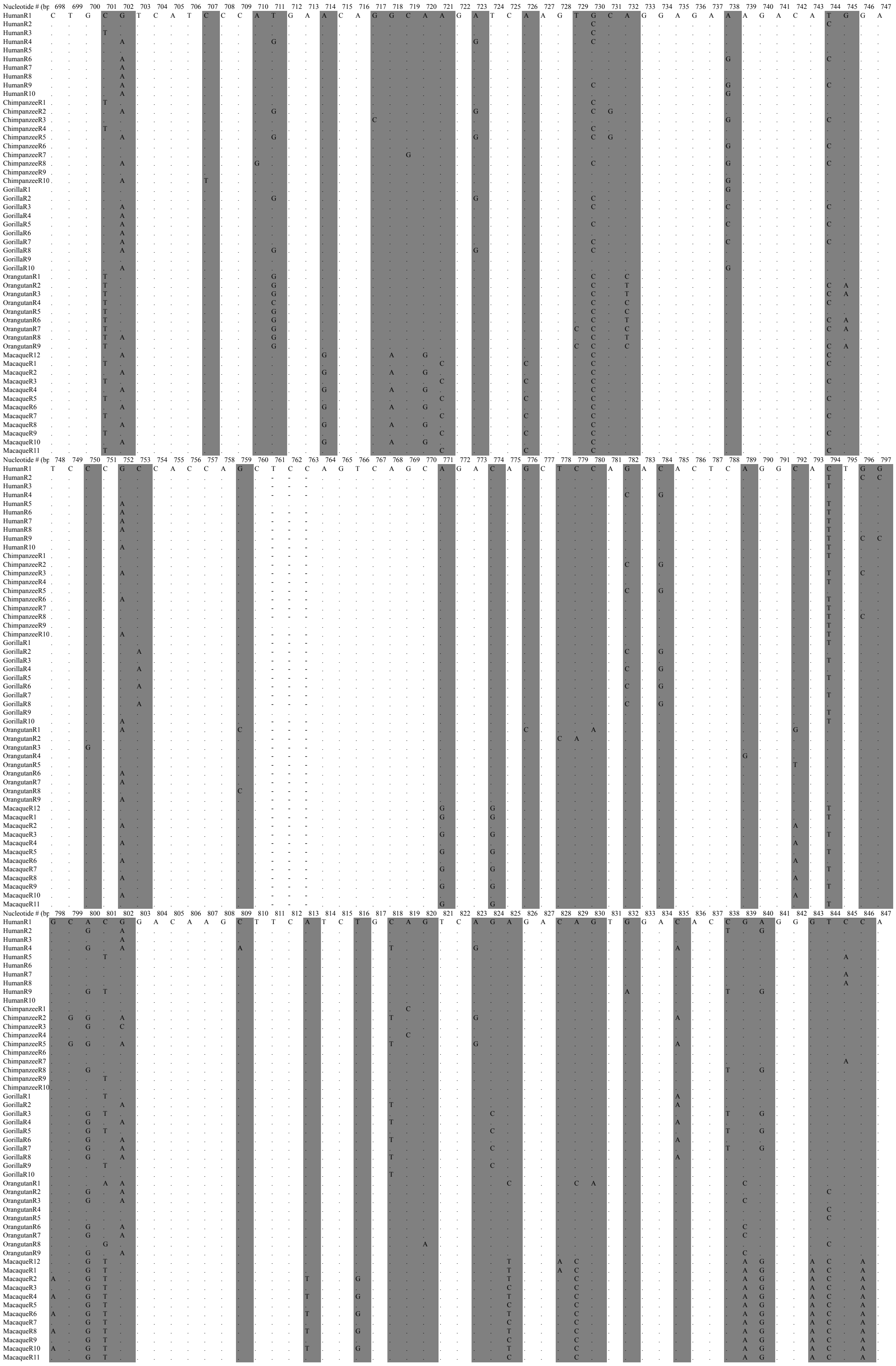

1

2

3

Nucleotide # (bp	848	849	850	851	852	853	854	855	856	857	858	859	860	861	862	863	864	865	866	867	868	869	870	871	872	873	874	875	876	877	878	879	880	881	882	883	884	885	886	887	888	889	890	891	892	893	894	895	896	897
HumanR1	G	T	G	G	T	A	G	T	C	A	G	G	C	C	A	C	T	G	A	C	A	G	T	G	A	G	G	G	A	C	A	T	T	C	A	G	A	A	G	A	C	T	C	A	G	A	C	A	C	A
HumanR2	.	.	.	.	.	.	.	.	.	.	.	.	.	.	.	G	.	.	.	T	.	.	.	.	.	.	.	.	.	.	.	.	.	.	.	.	.	G	.	.	G	.	.	.	.	.	.	.	.	.
HumanR3	.	.	.	.	.	.	.	.	.	.	.	.	T	.	.	.	.	A	.	.	.	.	.	.	.	.	.	.	.	.	.	.	.	.	.	.	.	.	.	.	.	.	.	.	.	.	.	.	.	.
HumanR4	.	.	.	.	.	.	.	C	.	.	.	.	.	.	.	G	.	.	.	.	.	.	C	.	.	.	.	.	.	.	.	C	.	.	.	.	.	.	.	.	G	.	.	.	.	.	.	.	.	.
HumanR5	.	.	.	.	.	.	.	.	.	.	.	.	.	.	.	G	.	.	.	.	.	.	.	.	.	.	.	.	.	.	.	.	.	.	.	.	.	.	.	.	.	.	.	.	.	.	.	.	.	.
HumanR6	.	.	.	.	.	.	.	.	.	.	.	.	.	.	.	G	.	.	.	.	.	.	.	.	.	.	.	.	.	.	.	.	.	.	.	.	.	.	.	.	.	.	.	.	.	.	.	.	.	.
HumanR7	.	.	.	.	.	.	.	.	.	.	.	.	.	.	.	G	.	.	.	.	.	A	.	.	.	.	.	.	.	.	.	.	.	.	.	.	.	.	.	.	.	.	.	.	.	.	.	.	.	.
HumanR8	.	.	.	.	.	.	.	.	.	.	.	.	.	.	.	G	.	.	.	.	.	A	.	.	.	.	.	.	.	.	.	.	.	.	.	.	.	.	.	.	.	.	.	.	.	.	.	.	.	.
HumanR9	.	.	.	.	.	.	.	.	.	.	.	.	.	.	.	G	.	.	.	T	.	.	.	.	.	.	.	.	.	.	.	.	.	.	.	.	.	.	.	.	G	.	.	.	.	.	.	.	.	.
HumanR10	.	.	.	.	.	.	.	.	.	.	.	.	.	.	.	G	.	.	.	.	.	.	.	.	.	.	.	.	.	.	.	.	.	.	.	.	.	.	.	.	.	.	.	.	.	.	.	.	.	.
ChimpanzeeR1	.	.	.	.	.	.	.	.	.	.	.	.	.	.	.	T	.	.	.	.	.	.	.	.	.	.	.	.	.	.	.	.	.	.	.	.	.	.	.	.	.	.	.	.	.	.	.	.	.	.
ChimpanzeeR2	.	.	.	.	.	.	.	.	.	.	.	.	.	.	.	G	.	.	.	.	.	.	C	.	.	.	.	.	.	.	.	C	.	.	.	.	.	.	.	.	.	.	.	.	.	.	.	.	.	.
ChimpanzeeR3	.	.	.	.	.	.	.	.	.	.	.	.	.	.	.	G	.	.	.	.	.	.	.	.	.	.	.	.	.	.	.	.	.	.	.	.	.	.	.	.	G	.	.	.	.	.	.	.	.	.
ChimpanzeeR4	.	.	.	.	.	.	.	.	.	.	.	.	.	.	.	T	.	.	.	.	.	.	.	.	.	.	.	.	.	.	.	.	.	.	.	.	.	.	.	.	.	.	.	.	.	.	.	.	.	.
ChimpanzeeR5	.	.	.	.	.	.	.	.	.	.	.	.	.	.	.	G	.	.	.	.	.	.	C	.	.	.	.	.	.	.	.	C	.	.	.	.	.	.	.	.	.	.	.	.	.	.	.	.	.	.
ChimpanzeeR6	.	.	.	.	.	.	.	.	.	.	.	.	.	.	.	G	.	.	.	.	.	.	.	.	.	.	.	.	.	.	.	.	.	.	.	.	.	.	.	.	.	.	.	.	.	.	.	.	.	.
ChimpanzeeR7	.	.	.	.	.	.	.	.	.	.	.	.	.	.	.	G	.	.	.	.	.	.	.	.	.	.	.	.	.	.	.	.	.	.	.	.	.	.	.	.	.	.	.	G	.	.	.	.	.	.
ChimpanzeeR8	.	.	.	.	.	.	.	.	.	.	.	.	.	.	.	G	.	.	.	T	.	.	.	.	.	.	.	.	.	.	.	.	.	.	.	.	.	.	.	.	G	.	.	.	.	.	.	.	.	.
ChimpanzeeR9	.	.	.	.	.	.	.	.	.	.	.	.	.	.	.	T	.	.	.	.	.	.	.	.	.	.	.	.	.	.	.	.	.	.	.	.	.	.	.	.	.	.	.	.	.	.	.	.	.	.
ChimpanzeeR10	.	.	.	.	.	.	.	.	.	.	.	.	.	.	.	G	.	.	.	.	.	.	.	.	.	.	.	.	.	.	.	.	.	.	.	.	.	.	.	.	.	.	.	.	.	.	.	.	.	.
GorillaR1	.	.	.	.	.	.	.	.	.	.	.	.	.	.	.	G	.	.	.	.	.	.	.	.	.	A	.	.	.	.	.	.	.	.	.	.	.	.	.	.	.	.	.	.	.	.	.	.	.	.
GorillaR2	.	.	.	.	.	.	.	.	.	.	.	.	.	.	.	G	.	.	.	.	.	.	C	.	.	.	.	.	.	.	.	C	.	.	.	.	.	.	.	.	.	.	.	.	.	.	.	.	.	.
GorillaR3	.	.	.	.	.	.	.	.	.	.	.	.	.	.	.	G	.	.	.	.	.	.	.	.	.	.	.	.	.	.	.	.	.	.	.	.	.	.	.	.	G	.	.	.	.	.	.	.	.	.
GorillaR4	.	.	.	.	.	.	.	.	.	.	.	.	.	.	.	G	.	.	.	.	.	.	C	.	.	.	.	.	.	.	.	C	.	.	.	.	.	.	.	.	.	.	.	.	.	.	.	.	.	.
GorillaR5	.	.	.	.	.	.	.	.	.	.	.	.	.	.	.	G	.	.	.	.	.	.	.	.	.	.	.	.	G	.	.	.	.	.	.	.	.	.	.	.	G	.	.	.	.	.	.	.	.	.
GorillaR6	.	.	.	.	.	.	.	.	.	.	.	.	.	.	.	G	.	.	.	.	.	.	C	.	.	.	.	.	.	.	.	C	.	.	.	.	.	.	.	.	.	.	.	.	.	.	.	.	.	.
GorillaR7	.	.	.	.	.	.	.	.	.	.	.	.	.	.	.	G	.	.	.	.	.	.	.	.	.	.	.	.	.	.	.	.	.	.	.	.	.	.	.	.	G	.	.	.	.	.	.	.	.	.
GorillaR8	.	.	.	.	.	.	.	.	.	.	.	.	.	.	.	G	.	.	.	.	.	.	C	.	.	.	.	.	.	.	.	C	.	.	.	.	.	.	.	.	G	.	.	.	.	.	.	.	.	.
GorillaR9	.	.	.	.	.	.	.	.	.	.	.	.	.	.	.	G	.	.	.	.	.	.	.	.	.	.	.	.	.	.	.	.	.	.	.	.	.	.	.	.	.	.	.	.	.	.	.	.	.	.
GorillaR10	.	.	.	.	.	.	.	.	.	.	.	.	.	.	.	G	.	.	.	.	.	.	.	.	.	.	.	.	.	.	.	.	.	.	.	.	.	.	.	.	.	.	.	.	.	.	.	.	.	.
OrangutanR1	.	.	.	.	.	.	.	.	.	.	.	.	.	.	.	A	.	C	.	.	.	.	.	C	.	.	.	.	.	.	.	C	.	.	.	.	.	.	.	.	.	.	.	.	.	.	.	.	.	.
OrangutanR2	T	.	.	.	.	.	.	.	.	.	.	.	.	.	.	G	.	C	.	.	.	.	.	.	.	.	.	.	.	.	.	.	.	.	.	.	.	.	.	.	.	.	.	.	.	.	.	.	.	.
OrangutanR3	.	.	.	.	.	.	.	.	.	.	.	.	.	.	.	G	A	.	.	.	.	.	.	.	.	.	.	.	.	.	.	.	.	.	.	.	.	.	.	.	.	.	.	.	.	.	.	.	.	.
OrangutanR4	T	.	.	.	.	.	.	.	.	.	.	.	.	.	.	G	.	C	.	.	.	.	.	.	.	.	.	.	.	.	.	.	.	.	.	.	.	.	.	.	.	.	.	.	.	.	.	.	.	.
OrangutanR5	T	.	.	.	.	.	.	.	.	.	.	.	.	.	.	G	.	C	.	.	.	.	.	.	.	.	.	.	.	.	.	.	.	.	.	.	.	.	.	.	.	.	.	.	.	.	.	.	.	.
OrangutanR6	.	.	.	.	.	.	.	.	.	.	.	.	.	.	.	G	A	.	.	.	.	.	.	.	.	.	.	.	.	.	.	.	.	.	.	.	.	.	.	.	.	.	.	.	.	.	.	.	.	.
OrangutanR7	.	.	.	.	.	.	.	.	.	.	.	.	.	.	.	G	.	.	.	.	.	.	.	.	.	.	.	.	.	.	.	.	.	.	.	.	.	.	.	.	.	.	.	.	.	.	.	.	.	.
OrangutanR8	T	.	.	.	.	.	.	.	.	.	.	.	.	.	.	G	.	C	.	.	.	.	.	.	.	.	.	.	.	.	.	.	.	.	.	.	.	.	.	.	.	.	.	.	.	.	.	.	.	.
OrangutanR9	.	.	.	.	.	.	A	.	.	.	.	.	.	.	.	G	.	.	.	.	.	.	.	.	.	.	.	.	.	.	.	.	.	.	.	.	.	.	.	.	.	.	.	.	.	.	.	.	.	.
MacaqueR12	.	C	.	.	.	.	.	.	.	.	.	T	T	.	.	G	.	.	.	.	.	.	.	.	.	.	.	.	.	.	.	.	.	.	.	.	.	.	C	.	.	.	.	.	.	.	.	.	.	.
MacaqueR1	.	C	.	.	.	.	.	.	.	.	.	T	T	.	.	G	.	.	.	.	.	.	.	.	.	.	.	.	.	.	.	.	.	.	.	.	.	.	C	.	.	.	.	.	.	.	.	.	.	.
MacaqueR2	.	C	.	.	.	.	.	.	.	.	C	T	T	.	.	G	.	.	.	.	.	.	.	.	.	.	.	.	.	.	.	.	.	.	.	.	.	.	.	.	.	.	.	.	.	.	.	.	.	.
MacaqueR3	.	C	.	.	.	.	.	.	.	.	.	T	T	.	.	G	.	.	.	.	.	.	.	.	.	.	.	.	.	.	.	.	.	.	.	.	.	.	C	.	.	.	.	.	.	.	.	.	.	.
MacaqueR4	.	C	.	.	.	.	.	.	.	.	C	T	T	.	.	G	.	.	.	.	.	.	.	.	.	.	.	.	.	.	.	.	.	.	.	.	.	.	.	.	.	.	.	.	.	.	.	.	.	.
MacaqueR5	.	C	.	.	.	.	.	.	.	.	.	T	T	.	.	G	.	.	.	.	.	.	.	.	.	.	.	.	.	.	.	.	.	.	.	.	.	.	C	.	.	.	.	.	.	.	.	.	.	.
MacaqueR6	.	C	.	.	.	.	.	.	.	.	C	T	T	.	.	G	.	.	.	.	.	.	.	.	.	.	.	.	.	.	.	.	.	.	.	.	.	.	.	.	.	.	.	.	.	.	.	.	.	.
MacaqueR7	.	C	.	.	.	.	.	.	.	.	.	T	T	.	.	G	.	.	.	.	.	.	.	.	.	.	.	.	.	.	.	.	.	.	.	.	.	.	C	.	.	.	.	.	.	.	.	.	.	.
MacaqueR8	.	C	.	.	.	.	.	.	.	.	C	T	T	.	.	G	.	.	.	.	.	.	.	.	.	.	.	.	.	.	.	.	.	.	.	.	.	.	.	.	.	.	.	.	.	.	.	.	.	.
MacaqueR9	.	C	.	.	.	.	.	.	.	.	.	T	T	.	.	G	.	.	.	.	.	.	.	.	.	.	.	.	.	.	.	.	.	.	.	.	.	.	C	.	.	.	.	.	.	.	.	.	.	.
MacaqueR10	.	C	.	.	.	.	.	.	.	.	C	T	T	.	.	G	.	.	.	.	.	.	.	.	.	.	.	.	.	.	.	.	.	.	.	.	.	.	.	.	.	.	.	.	.	.	.	.	.	.
MacaqueR11	.	C	.	.	.	.	.	.	.	.	.	T	T	.	.	G	.	.	.	.	.	.	.	.	.	.	.	.	.	.	.	.	.	.	.	.	.	.	C	.	.	.	.	.	.	.	.	.	.	.

Nucleotide # (bp	898	899	900	901	902	903	904	905	906	907	908	909	910	911	912	913	914	915	916	917	918	919	920	921	922	923	924	925	926	927	928	929	930	931	932	933	934	935	936	937	938	939	940	941	942	943	944	945	946	947
HumanR1	C	A	G	T	C	A	G	T	G	T	C	A	G	G	C	C	A	T	G	G	A	C	A	G	G	C	T	G	G	T	C	A	C	C	A	T	C	A	G	C	A	G	A	G	C	C	A	C	C	A
HumanR2	.	.	.	.	.	.	.	.	.	.	.	.	.	.	.	.	.	.	.	.	.	.	.	.	.	A	.	.	.	G	.	C	.	.	.	.	.	.	.	.	.	.	.	.	.	.	.	.	.	.
HumanR3	.	.	.	.	.	.	.	.	.	.	.	.	.	C	.	.	.	C	.	.	.	.	.	A	.	.	.	.	.	G	.	C	.	.	.	.	.	.	.	.	.	.	.	.	.	.	.	.	A	.
HumanR4	.	.	.	.	.	.	.	.	.	.	.	.	.	C	.	.	.	C	.	.	.	.	.	.	.	.	.	.	.	G	.	C	.	.	.	.	.	.	.	.	.	.	.	.	.	.	.	.	.	.
HumanR5	.	.	.	.	.	.	.	.	.	.	.	.	.	C	A	.	.	G	.	.	.	A	.	A	.	.	.	.	.	G	.	C	.	.	.	.	.	.	.	.	.	.	.	.	.	.	.	.	A	.
HumanR6	.	.	.	.	.	.	.	.	.	.	.	.	.	C	.	.	.	C	.	.	.	.	.	.	.	.	.	.	.	G	.	C	.	.	.	.	.	.	.	.	.	.	.	.	.	.	.	.	.	.
HumanR7	.	.	.	.	.	.	.	.	.	.	.	.	.	C	.	.	.	C	.	.	.	.	.	.	.	.	.	.	.	G	T	C	.	.	.	.	.	.	.	.	.	.	.	.	.	.	.	.	.	.
HumanR8	.	.	.	.	.	.	.	.	.	.	.	.	.	C	.	.	.	C	.	.	.	.	.	.	.	.	.	.	.	G	T	C	.	.	.	.	.	.	.	.	.	.	.	.	.	.	.	.	.	.
HumanR9	.	.	.	.	.	.	.	.	.	.	.	.	.	.	.	.	.	.	.	.	.	.	.	.	.	.	.	.	.	G	.	C	.	.	.	.	.	.	.	.	.	.	.	.	.	.	.	.	.	.
HumanR10	.	.	.	.	.	.	.	.	.	.	.	.	.	C	.	.	.	C	.	.	.	.	.	.	.	.	.	.	.	G	.	C	.	.	.	.	.	.	.	.	.	.	.	.	.	.	.	.	.	.
ChimpanzeeR1	.	.	.	.	.	.	.	.	.	.	.	.	T	C	.	.	.	C	.	.	.	.	.	A	.	.	.	.	.	G	.	C	.	.	.	.	.	.	.	.	.	.	.	.	.	.	.	.	A	.
ChimpanzeeR2	.	.	.	.	.	.	.	.	.	.	.	.	.	C	.	.	.	C	.	.	.	.	.	.	.	.	.	.	.	G	.	C	.	.	G	.	.	.	.	.	.	.	.	.	.	.	.	.	.	.
ChimpanzeeR3	.	.	.	.	.	.	.	.	.	.	.	.	.	.	.	.	.	.	.	.	.	.	.	.	.	A	.	.	.	G	.	C	.	.	.	.	.	.	.	.	.	.	.	.	.	.	.	.	.	.
ChimpanzeeR4	.	.	.	.	.	.	.	.	.	.	.	.	T	C	.	.	.	C	.	.	.	.	.	A	.	.	.	.	.	G	.	C	.	.	.	.	.	.	.	.	.	.	.	.	.	.	.	.	A	.
ChimpanzeeR5	.	.	.	.	.	.	.	.	.	.	.	.	.	C	.	.	.	C	.	.	.	.	.	.	.	.	.	.	.	G	.	C	.	.	G	.	.	.	.	.	.	.	.	.	.	.	.	.	.	.
ChimpanzeeR6	.	.	.	.	.	.	C	.	.	.	.	.	.	C	.	.	.	C	.	.	.	.	.	.	.	.	.	.	.	G	.	C	.	.	.	.	.	.	.	.	.	.	.	.	.	.	.	.	.	.
ChimpanzeeR7	.	.	.	.	.	.	.	.	.	.	.	.	.	C	.	.	.	C	.	.	.	.	.	.	.	.	.	.	.	G	T	C	.	.	.	.	.	.	.	.	.	.	.	.	.	.	.	.	.	.
ChimpanzeeR8	.	.	.	.	.	.	.	.	.	.	.	.	.	.	.	.	.	.	.	.	.	.	.	.	.	A	.	.	.	G	.	C	.	.	.	.	.	.	.	.	.	.	.	.	.	.	.	.	.	.
ChimpanzeeR9	.	.	.	.	.	.	.	.	.	.	.	.	.	C	.	.	.	G	.	.	.	.	.	A	.	.	.	.	.	G	.	C	.	.	.	.	.	.	.	.	G	.	.	.	.	.	.	.	A	.
ChimpanzeeR10	.	.	.	.	.	.	.	.	.	.	.	.	.	C	.	.	.	.	.	.	.	.	.	.	.	.	.	.	.	G	.	C	.	.	.	.	.	.	.	.	.	.	.	.	.	.	.	.	.	.
GorillaR1	.	.	.	.	.	.	.	.	.	.	.	.	.	C	.	.	.	C	.	.	.	.	.	.	.	.	.	.	.	G	.	C	.	.	G	.	.	.	.	.	.	.	.	.	.	.	.	.	.	.
GorillaR2	.	.	.	.	.	.	.	.	.	.	.	.	.	C	.	.	.	C	.	.	.	.	.	.	.	.	.	.	.	G	.	C	.	.	G	.	.	.	.	.	.	.	.	.	.	.	.	.	.	.
GorillaR3	.	.	.	.	.	.	.	.	.	.	.	.	.	.	.	.	.	.	.	.	.	.	.	.	.	.	.	.	.	G	.	C	.	.	.	.	.	.	.	.	.	.	.	.	.	.	.	.	.	.
GorillaR4	.	.	.	.	.	.	.	.	.	.	.	.	.	C	.	.	.	C	.	.	.	.	.	.	.	.	.	.	.	G	.	C	.	.	G	.	.	.	.	.	.	.	.	.	.	.	.	.	.	.
GorillaR5	.	.	.	.	.	.	.	.	.	.	.	.	.	.	.	.	.	.	.	.	.	.	.	.	.	.	.	.	.	G	.	C	.	.	.	.	.	.	.	.	.	.	.	.	.	.	.	.	.	.
GorillaR6	.	.	.	.	.	.	.	.	.	.	.	.	.	C	.	.	.	C	.	.	.	.	.	.	.	.	.	.	.	G	.	C	.	.	G	.	.	.	.	.	.	.	.	.	.	.	.	.	.	.
GorillaR7	.	.	.	.	.	.	.	.	.	.	.	.	.	.	.	.	.	.	.	.	.	.	.	.	.	.	.	.	.	G	.	C	.	.	.	.	.	.	.	.	.	.	.	.	.	.	.	.	.	.
GorillaR8	.	.	.	.	.	.	.	.	.	.	.	.	.	C	.	.	.	C	.	.	.	.	.	.	.	.	.	.	.	G	.	C	.	.	G	.	.	.	.	.	.	.	.	.	.	.	.	.	.	.
GorillaR9	.	.	.	.	.	.	.	.	.	.	.	.	.	C	.	.	.	C	.	.	.	.	.	.	.	.	.	.	.	G	.	C	.	.	G	.	.	.	.	.	.	.	.	.	.	.	.	.	.	.
GorillaR10	.	.	.	.	.	.	.	.	.	.	.	.	.	C	.	.	.	C	.	.	.	.	.	.	.	.	.	.	.	G	.	C	.	.	.	.	.	.	.	.	.	.	.	.	.	.	.	.	.	.
OrangutanR1	.	.	.	.	.	.	.	.	A	.	.	.	C	C	.	.	.	C	.	.	.	.	.	.	.	.	.	.	.	G	G	C	.	.	.	.	.	G	.	.	.	.	.	.	.	.	.	.	.	.
OrangutanR2	.	.	.	.	.	.	.	.	.	.	.	.	.	C	A	.	.	C	.	.	.	.	.	.	C	.	.	A	.	G	.	C	.	.	.	.	.	.	.	.	.	.	.	.	.	.	.	.	.	.
OrangutanR3	.	.	.	.	.	.	.	.	.	.	.	.	.	C	.	.	.	.	.	.	.	.	.	.	.	.	.	.	.	G	.	C	.	.	.	.	.	.	.	.	.	.	.	.	.	.	.	.	A	.
OrangutanR4	.	.	.	.	.	.	.	.	.	.	.	.	.	C	.	.	.	.	.	.	.	.	.	.	.	.	.	.	.	G	.	C	.	.	.	.	.	.	.	.	.	.	.	.	.	.	.	.	A	.
OrangutanR5	.	.	.	.	.	.	.	.	.	.	.	.	.	C	.	.	.	.	.	.	.	.	.	.	.	.	.	.	.	G	.	C	.	.	.	.	.	.	.	.	.	.	.	.	G	.	.	.	.	.
OrangutanR6	.	.	.	.	.	.	.	.	.	.	.	.	.	C	.	.	.	.	.	.	.	.	.	.	.	A	.	.	.	G	.	C	.	.	.	.	.	.	.	.	.	.	.	.	.	.	.	.	.	.
OrangutanR7	.	.	.	.	.	.	.	.	.	.	.	.	.	C	.	.	.	.	.	.	.	.	.	.	.	.	.	.	.	G	.	C	.	.	.	.	.	.	.	.	.	.	.	.	.	.	.	.	.	.
OrangutanR8	.	.	.	.	.	.	.	.	.	.	.	.	C	C	.	.	.	C	.	.	.	.	.	.	C	.	.	A	.	G	.	C	.	.	.	.	.	.	.	.	.	.	.	.	.	.	.	.	.	.
OrangutanR9	.	.	.	.	.	.	.	.	.	.	.	.	.	C	.	.	.	.	.	A	.	.	.	.	.	.	.	.	.	G	.	C	.	.	.	.	.	.	.	.	.	.	.	.	.	.	.	.	.	.
MacaqueR12	.	.	.	.	.	.	.	.	.	.	.	.	.	.	.	.	.	C	.	.	.	.	.	.	.	.	.	.	.	A	.	C	.	.	.	.	.	C	.	.	.	C	.	.	.	.	.	.	.	.
MacaqueR1	.	.	.	.	.	.	.	.	.	.	.	.	.	.	.	.	.	C	.	.	.	.	.	.	.	.	.	.	.	A	.	C	.	.	.	.	.	C	.	.	.	C	.	.	.	.	.	.	.	.
MacaqueR2	.	.	.	.	.	.	.	.	.	.	.	.	.	.	.	.	.	G	.	.	.	.	.	.	.	.	.	.	.	G	.	G	.	.	.	.	.	C	.	.	.	C	.	.	.	.	.	.	.	.
MacaqueR3	.	.	.	.	.	.	.	.	.	.	.	.	.	.	.	.	.	C	.	.	.	.	.	.	.	.	.	.	.	A	.	C	.	.	.	.	.	C	.	.	.	C	.	.	.	.	.	.	.	.
MacaqueR4	.	.	.	.	.	.	.	.	.	.	.	.	.	.	.	.	.	G	.	.	.	.	.	.	.	.	.	.	.	G	.	G	.	.	.	.	.	C	.	.	.	C	.	.	.	.	.	.	.	.
MacaqueR5	.	.	.	.	.	.	.	.	.	.	.	.	.	.	.	.	.	C	.	.	.	.	.	.	.	.	.	.	.	A	.	C	.	.	.	.	.	C	.	.	.	C	.	.	.	.	.	.	.	.
MacaqueR6	.	.	.	.	.	.	.	.	.	.	.	.	.	.	.	.	.	G	.	.	.	.	.	.	.	.	.	.	.	G	.	G	.	.	.	.	.	C	.	.	.	C	.	.	.	.	.	.	.	.
MacaqueR7	.	.	.	.	.	.	.	.	.	.	.	.	.	.	.	.	.	C	.	.	.	.	.	.	.	.	.	.	.	A	.	C	.	.	.	.	.	C	.	.	.	C	.	.	.	.	.	.	.	.
MacaqueR8	.	.	.	.	.	.	.	.	.	.	.	.	.	.	.	.	.	G	.	.	.	.	.	.	.	.	.	.	.	G	.	G	.	.	.	.	.	C	.	.	.	C	.	.	.	.	.	.	.	.
MacaqueR9	.	.	.	.	.	.	.	.	.	.	.	.	.	.	.	.	.	C	.	.	.	.	.	.	.	.	.	.	.	A	.	C	.	.	.	.	.	C	.	.	.	C	.	.	.	.	.	.	.	.
MacaqueR10	.	.	.	.	.	.	.	.	.	.	.	.	.	.	.	.	.	G	.	.	.	.	.	.	.	.	.	.	.	G	.	G	.	.	.	.	.	C	.	.	.	C	.	.	.	.	.	.	.	.
MacaqueR11	.	.	.	.	.	.	.	.	.	.	.	.	.	.	.	.	.	C	.	.	.	.	.	.	.	.	.	.	.	A	.	C	.	.	.	.	.	C	.	.	.	C	.	.	.	.	.	.	.	.

Nucleotide # (bp	948	949	950	951	952	953	954	955	956	957	958	959	960	961	962	963	964	965	966	967	968	969	970	971	972	973	974	975
HumanR1	A	G	A	G	T	C	C	G	C	A	C	G	T	G	A	C	C	G	G	T	C	A	G	G	G	G	A	A
HumanR2	.	.	.	.	.	.	.	.	.	.	.	.	.	.	.	.	T	.	.	.	.	.	.	.	.	.	G	.
HumanR3	.	.	.	.	.	.	.	.	.	.	.	.	.	.	G	.	.	A	.	.	.	.	.	.	.	.	.	.
HumanR4	.	.	.	.	.	.	.	A	.	.	.	.	.	.	G	.	.	A	.	.	.	.	.	.	.	.	.	.
HumanR5	.	.	.	.	.	.	.	.	.	.	.	.	.	.	G	.	.	A	.	.	.	.	.	.	.	.	.	.
HumanR6	.	.	.	.	.	.	.	A	.	.	.	.	.	.	G	.	.	.	.	.	.	.	.	C	A	.	G	.
HumanR7	.	.	.	.	.	.	.	.	.	.	.	.	.	.	G	.	.	.	.	.	.	.	.	.	.	.	.	.
HumanR8	.	.	.	.	.	.	.	.	.	.	.	.	.	.	G	.	.	.	.	.	.	.	.	.	.	.	.	.
HumanR9	.	.	.	.	.	.	.	.	.	.	.	.	.	.	.	.	.	.	.	.	.	.	.	.	.	.	G	.
HumanR10	.	.	.	.	.	.	.	A	.	.	.	.	.	.	G	.	.	.	.	.	.	.	.	C	A	.	G	.
ChimpanzeeR1	.	.	.	.	.	.	.	.	.	.	.	.	.	.	G	.	.	A	.	.	.	.	.	.	.	.	.	.
ChimpanzeeR2	.	.	.	.	.	.	.	A	.	.	.	.	.	.	G	.	.	A	.	.	.	.	.	.	.	.	.	.
ChimpanzeeR3	.	.	.	.	.	.	.	.	.	.	.	.	.	.	.	.	T	.	.	.	.	.	.	.	.	.	G	.
ChimpanzeeR4	.	.	.	.	.	.	.	.	.	.	.	.	.	.	G	.	.	A	.	.	.	.	.	.	.	.	.	.
ChimpanzeeR5	.	.	.	.	.	.	.	A	.	.	.	.	.	.	G	.	.	A	.	.	.	.	.	.	.	.	.	.
ChimpanzeeR6	.	.	.	.	.	.	.	A	.	.	.	.	.	.	G	.	.	.	.	.	.	.	.	C	.	.	.	.
ChimpanzeeR7	.	.	.	.	.	.	.	.	.	.	.	.	.	.	G	.	.	.	.	.	.	.	.	.	.	.	.	.
ChimpanzeeR8	.	.	.	.	.	.	T	.	.	.	.	.	.	.	.	.	.	.	.	.	.	.	.	.	.	.	G	.
ChimpanzeeR9	.	.	.	.	.	.	T	.	.	.	.	.	.	.	G	.	.	A	.	.	.	.	.	.	.	.	.	.
ChimpanzeeR10	.	.	.	.	.	.	.	A	.	.	.	.	.	.	G	.	.	.	.	.	.	.	.	C	A	.	.	.
GorillaR1	.	.	.	.	.	.	.	C	.	.	.	A	.	.	G	.	.	.	.	.	.	.	.	.	.	.	.	.
GorillaR2	.	.	.	.	.	.	.	A	.	.	.	.	.	.	C	.	.	A	.	.	.	.	.	.	.	.	.	.
GorillaR3	.	.	.	.	.	.	.	A	.	.	.	.	.	.	.	.	.	.	.	.	.	.	.	.	.	.	G	.
GorillaR4	.	.	.	.	.	.	.	A	.	.	.	.	.	.	C	.	.	A	.	.	.	.	.	.	.	.	.	.
GorillaR5	.	.	.	.	.	.	.	A	.	.	.	.	.	.	.	.	.	.	.	.	.	.	.	.	.	.	G	.
GorillaR6	.	.	.	.	.	.	.	A	.	.	.	.	.	.	C	.	.	A	.	.	.	.	.	.	.	.	.	.
GorillaR7	.	.	.	.	.	.	.	A	.	.	.	.	.	.	.	.	.	.	.	.	.	.	.	.	.	.	G	.
GorillaR8	.	.	.	.	.	.	.	A	.	.	.	.	.	.	C	.	.	A	.	.	.	.	A	.	.	.	.	.
GorillaR9	.	.	.	.	.	.	.	A	.	.	.	.	.	.	G	.	.	.	.	.	.	.	.	C	.	.	.	.
GorillaR10	.	.	.	.	.	.	.	A	.	.	.	.	.	.	G	.	.	A	.	.	.	.	.	C	A	.	.	.
OrangutanR1	.	.	.	.	.	.	.	.	.	.	.	A	.	.	G	.	.	.	.	.	.	.	.	.	.	.	.	.
OrangutanR2	.	.	.	.	.	.	.	.	.	.	.	.	.	.	G	.	.	A	.	.	.	.	.	.	.	.	.	.
OrangutanR3	.	.	.	.	.	.	A	.	.	.	.	A	.	.	G	.	.	.	.	.	.	.	.	.	.	.	.	.
OrangutanR4	.	.	.	.	.	.	.	.	.	.	.	A	.	.	G	.	.	.	.	.	.	.	.	.	.	.	.	.
OrangutanR5	.	.	.	.	.	.	.	.	.	.	.	.	.	.	G	.	T	.	.	.	.	.	.	.	.	.	.	.
OrangutanR6	.	.	.	.	.	.	.	.	.	.	.	A	.	.	G	.	T	.	.	.	.	.	.	.	.	.	.	.
OrangutanR7	.	.	.	.	.	.	.	.	.	.	.	.	.	.	G	.	.	.	.	.	.	.	.	.	.	.	.	.
OrangutanR8	.	.	.	.	.	.	T	.	.	.	.	.	.	.	G	T	.	A	.	.	.	.	.	.	.	.	.	.
OrangutanR9	.	.	.	.	.	.	.	.	.	.	.	.	.	.	G	.	.	.	.	.	.	.	.	.	.	.	.	.
MacaqueR12	.	.	.	.	.	.	T	.	.	.	.	.	.	.	G	.	.	.	.	.	.	.	.	.	.	.	.	.
MacaqueR1	.	.	.	.	.	.	T	.	.	.	.	.	.	.	G	.	.	.	.	.	.	.	.	.	.	.	.	.
MacaqueR2	.	.	.	.	.	.	T	.	.	.	.	.	.	.	G	.	.	.	.	.	.	.	.	.	.	.	.	.
MacaqueR3	.	.	.	.	.	.	T	.	.	.	.	.	.	.	G	.	.	.	.	.	.	.	.	.	.	.	.	.
MacaqueR4	.	.	.	.	.	.	T	.	.	.	.	.	.	.	G	.	.	.	.	.	.	.	.	.	.	.	.	.
MacaqueR5	.	.	.	.	.	.	T	.	.	.	.	.	.	.	G	.	.	.	.	.	.	.	.	.	.	.	.	.
MacaqueR6	.	.	.	.	.	.	T	.	.	.	.	.	.	.	G	.	.	.	.	.	.	.	.	.	.	.	.	.
MacaqueR7	.	.	.	.	.	.	T	.	.	.	.	.	.	.	G	.	.	.	.	.	.	.	.	.	.	.	.	.
MacaqueR8	.	.	.	.	.	.	T	.	.	.	.	.	.	.	G	.	.	.	.	.	.	.	.	.	.	.	.	.
MacaqueR9	.	.	.	.	.	.	T	.	.	.	.	.	.	.	G	.	.	.	.	.	.	.	.	.	.	.	.	.
MacaqueR10	.	.	.	.	.	.	T	.	.	.	.	.	.	.	G	.	.	.	.	.	.	.	.	.	.	.	.	.
MacaqueR11	.	.	.	.	.	.	T	.	.	.	.	.	.	.	G	.	.	.	.	.	.	.	.	.	.	.	.	.

B.

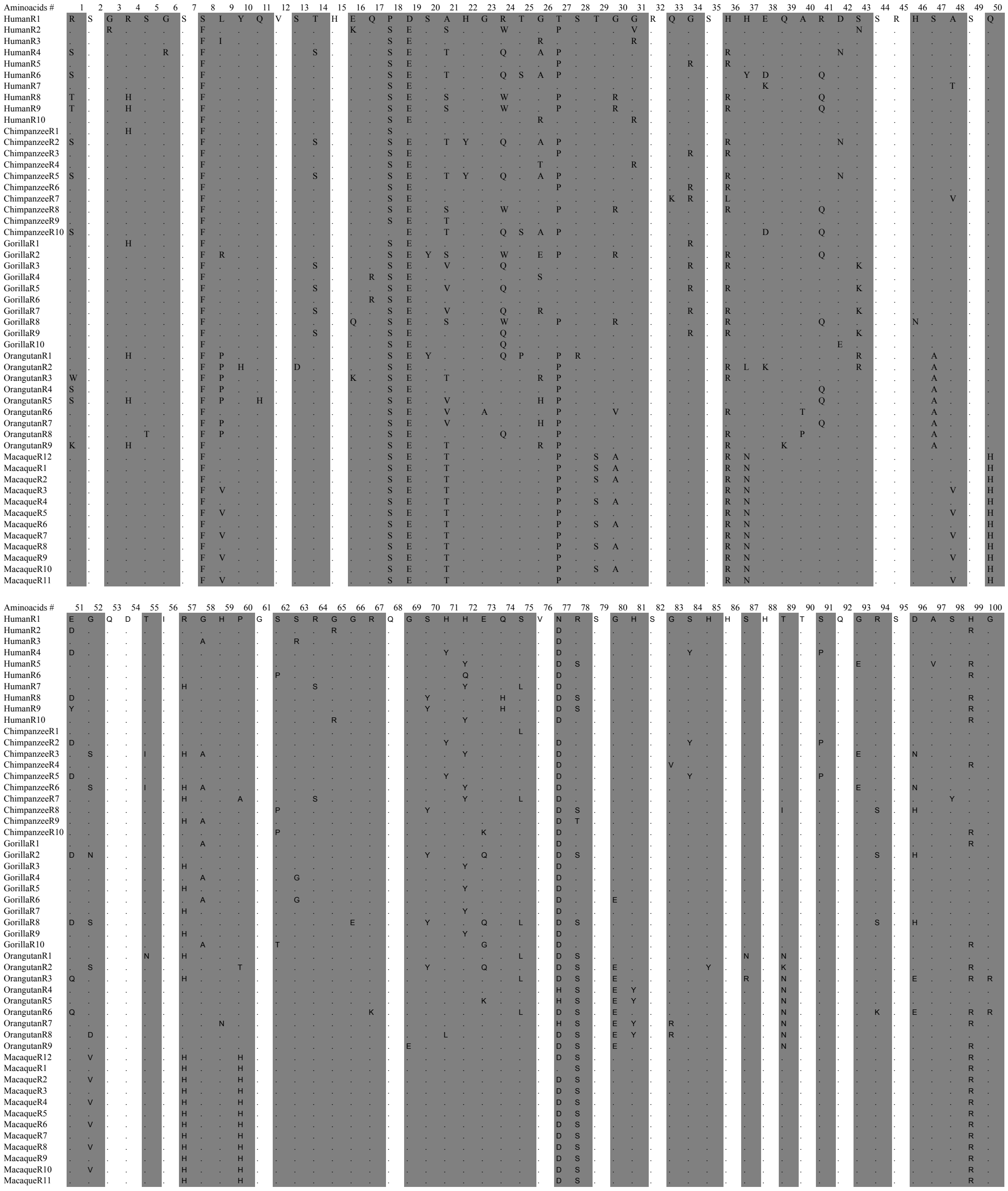

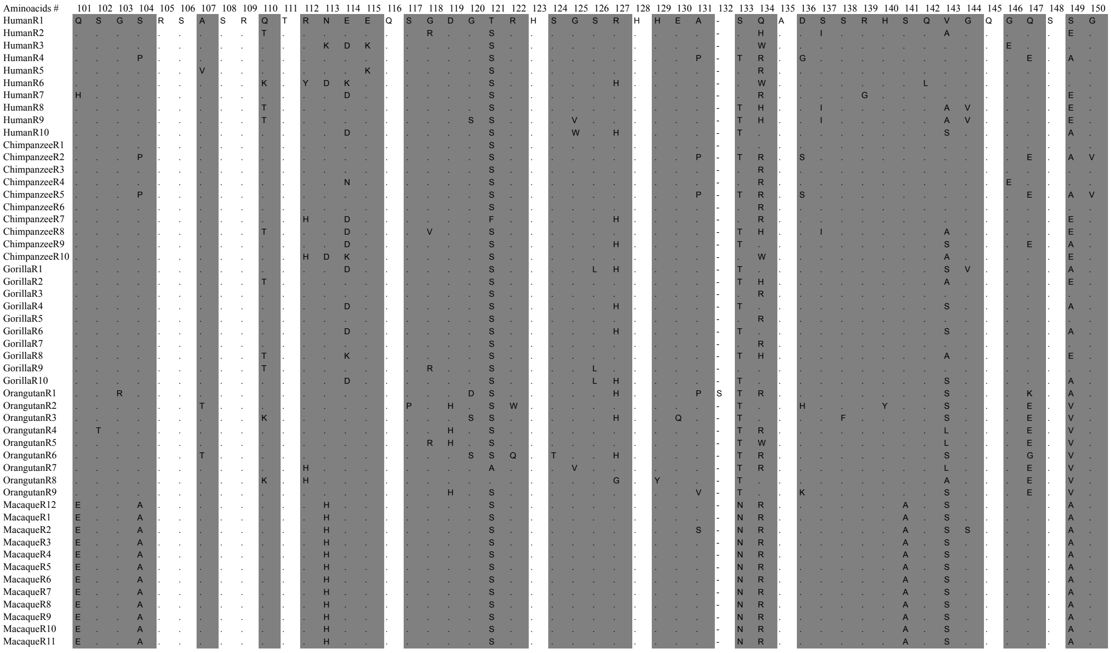

1

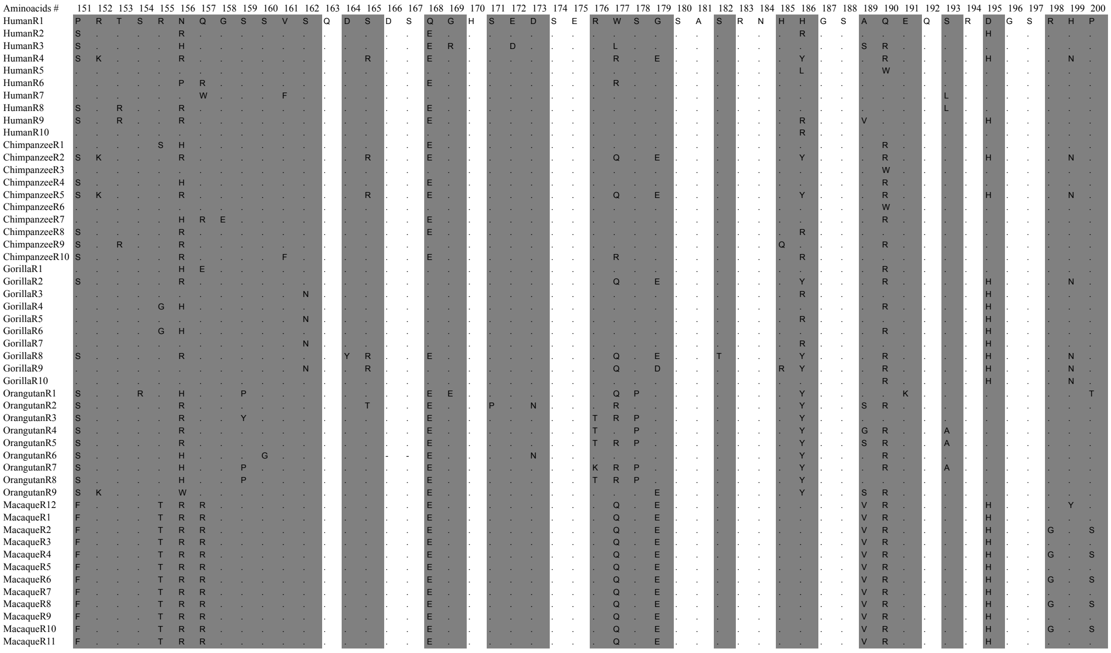

2

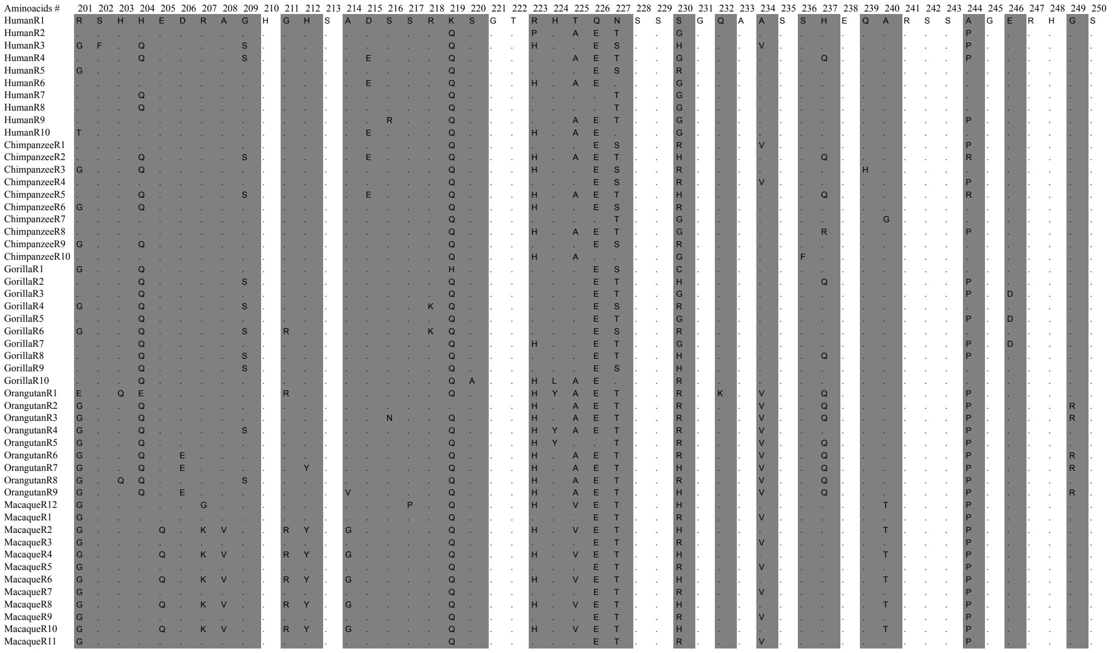

1

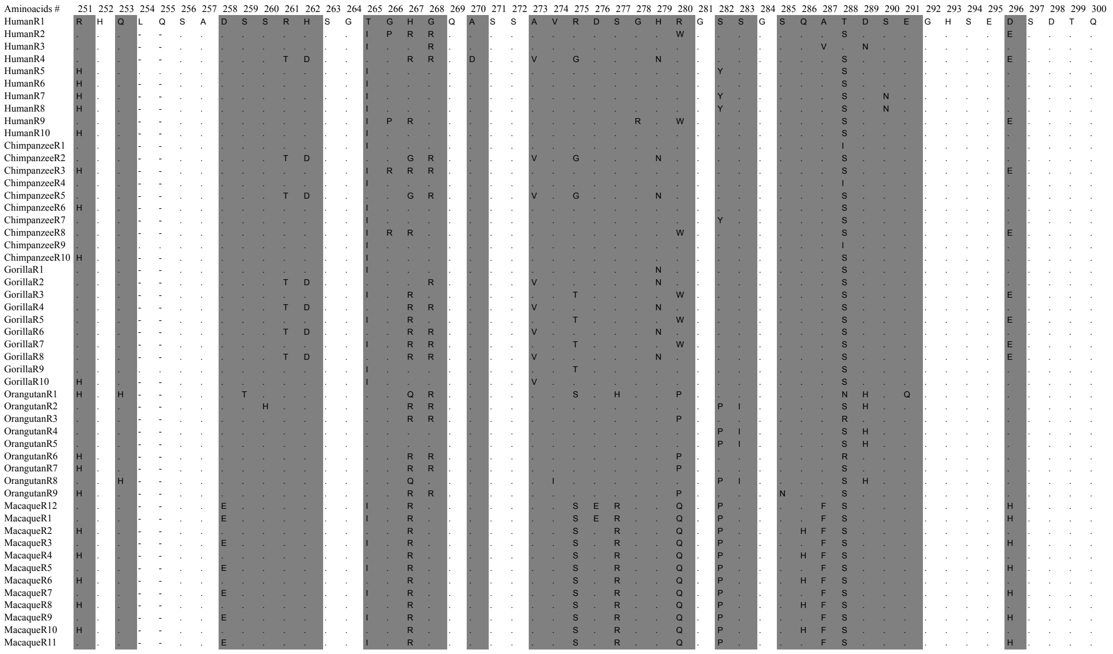

2

Aminoacids #	301	302	303	304	305	306	307	308	309	310	311	312	313	314	315	316	317	318	319	320	321	322	323	324	325
HumanR1	S	V	S	G	H	G	Q	A	G	H	H	Q	Q	S	H	Q	E	S	A	R	D	R	S	G	E
HumanR2	.	.	.	.	.	.	.	D	.	P	.	.	.	.	.	.	.	.	.	.	.	W	.	.	G
HumanR3	.	.	.	A	.	.	.	.	.	P	.	.	.	.	.	K	.	.	.	.	G	Q	.	.	.
HumanR4	.	.	.	A	.	.	.	.	.	P	.	.	.	.	.	.	.	.	T	.	G	Q	.	.	.
HumanR5	.	.	.	A	Q	.	K	.	.	P	.	.	.	.	.	K	.	.	.	.	G	Q	.	.	.
HumanR6	.	.	.	A	.	.	.	.	.	P	.	.	.	.	.	.	.	.	T	.	G	.	.	A	G
HumanR7	.	.	.	A	.	.	.	.	.	S	.	.	.	.	.	.	.	.	.	.	G	.	.	.	.
HumanR8	.	.	.	A	.	.	.	.	.	S	.	.	.	.	.	.	.	.	.	.	G	.	.	.	.
HumanR9	.	.	.	.	.	.	.	.	.	P	.	.	.	.	.	.	.	.	.	.	.	.	.	.	G
HumanR10	.	.	.	A	.	.	.	.	.	P	.	.	.	.	.	.	.	.	T	.	G	.	.	A	G
ChimpanzeeR1	.	.	.	S	.	.	.	.	.	P	.	.	.	.	.	K	.	.	.	.	G	Q	.	.	.
ChimpanzeeR2	.	.	.	A	.	.	.	.	.	P	R	.	.	.	.	.	.	.	T	.	G	Q	.	.	.
ChimpanzeeR3	.	.	.	.	.	.	.	D	.	P	.	.	.	.	.	.	.	.	.	.	.	W	.	.	G
ChimpanzeeR4	.	.	.	S	.	.	.	.	.	P	.	.	.	.	.	K	.	.	.	.	G	Q	.	.	.
ChimpanzeeR5	.	.	.	A	.	.	.	.	.	P	R	.	.	.	.	.	.	.	T	.	G	Q	.	.	.
ChimpanzeeR6	.	L	.	A	.	.	.	.	.	P	.	.	.	.	.	.	.	.	T	.	G	.	.	A	.
ChimpanzeeR7	.	.	.	A	.	.	.	.	.	S	.	.	.	.	.	.	.	.	.	.	G	.	.	.	.
ChimpanzeeR8	.	.	.	.	.	.	.	D	.	P	.	.	.	.	.	.	.	.	.	.	.	.	.	.	G
ChimpanzeeR9	.	.	.	A	Q	.	.	.	.	P	.	.	R	.	.	K	.	.	.	.	G	Q	.	.	.
ChimpanzeeR10	.	.	.	A	.	.	.	.	.	P	.	.	.	.	.	.	.	.	T	.	G	.	.	A	.
GorillaR1	.	.	.	A	.	.	.	.	.	P	R	.	.	.	.	.	.	.	P	H	G	.	.	.	.
GorillaR2	.	.	.	A	.	.	.	.	.	P	R	.	.	.	.	.	.	.	T	.	A	Q	.	.	.
GorillaR3	.	.	.	.	.	.	.	.	.	P	.	.	.	.	.	.	.	.	T	.	.	.	.	.	G
GorillaR4	.	.	.	A	.	.	.	.	.	P	R	.	.	.	.	.	.	.	T	.	A	Q	.	.	.
GorillaR5	.	.	.	.	.	.	.	.	.	P	.	.	.	.	.	.	.	.	T	.	.	.	.	.	G
GorillaR6	.	.	.	A	.	.	.	.	.	P	R	.	.	.	.	.	.	.	T	.	A	Q	.	.	.
GorillaR7	.	.	.	.	.	.	.	.	.	P	.	.	.	.	.	.	.	.	T	.	.	.	.	.	G
GorillaR8	.	.	.	A	.	.	.	.	.	P	R	.	.	.	.	.	.	.	T	.	A	Q	.	R	.
GorillaR9	.	.	.	A	.	.	.	.	.	P	R	.	.	.	.	.	.	.	T	.	G	.	.	A	.
GorillaR10	.	.	.	A	.	.	.	.	.	P	.	.	.	.	.	.	.	.	T	.	G	Q	.	A	.
OrangutanR1	.	.	.	P	.	.	.	.	.	A	.	R	.	.	.	.	.	.	.	H	G	.	.	.	.
OrangutanR2	.	.	.	A	.	.	.	P	R	P	.	.	.	.	.	.	.	.	.	.	G	Q	.	.	.
OrangutanR3	.	.	.	A	.	.	.	.	.	P	.	.	.	.	.	K	.	.	.	H	G	.	.	.	.
OrangutanR4	.	.	.	A	.	.	.	.	.	P	.	.	.	.	.	K	.	.	.	H	G	.	.	.	.
OrangutanR5	.	.	.	A	.	.	.	.	.	P	.	.	.	R	.	.	.	.	.	.	G	W	.	.	.
OrangutanR6	.	.	.	A	.	.	.	D	.	P	.	.	.	.	.	.	.	.	.	H	G	W	.	.	.
OrangutanR7	.	.	.	A	.	.	.	.	.	P	.	.	.	.	.	.	.	.	.	.	G	.	.	.	.
OrangutanR8	.	.	.	P	.	.	.	P	R	P	.	.	.	.	.	.	.	.	.	.	G	Q	.	.	.
OrangutanR9	.	.	.	A	.	E	.	.	.	P	.	.	.	.	.	.	.	.	.	.	G	.	.	.	.
MacaqueR12	.	.	.	.	.	.	.	.	.	P	.	P	H	.	.	.	.	.	.	.	G	.	.	.	.
MacaqueR1	.	.	.	.	.	.	.	.	.	P	.	P	H	.	.	.	.	.	.	.	G	.	.	.	.
MacaqueR2	.	.	.	.	Q	.	.	.	.	R	.	P	H	.	.	.	.	.	.	.	G	.	.	.	.
MacaqueR3	.	.	.	.	.	.	.	.	.	P	.	P	H	.	.	.	.	.	.	.	G	.	.	.	.
MacaqueR4	.	.	.	.	Q	.	.	.	.	R	.	P	H	.	.	.	.	.	.	.	G	.	.	.	.
MacaqueR5	.	.	.	.	.	.	.	.	.	P	.	P	H	.	.	.	.	.	.	.	G	.	.	.	.
MacaqueR6	.	.	.	.	Q	.	.	.	.	R	.	P	H	.	.	.	.	.	.	.	G	.	.	.	.
MacaqueR7	.	.	.	.	.	.	.	.	.	P	.	P	H	.	.	.	.	.	.	.	G	.	.	.	.
MacaqueR8	.	.	.	.	Q	.	.	.	.	R	.	P	H	.	.	.	.	.	.	.	G	.	.	.	.
MacaqueR9	.	.	.	.	.	.	.	.	.	P	.	P	H	.	.	.	.	.	.	.	G	.	.	.	.
MacaqueR10	.	.	.	.	Q	.	.	.	.	R	.	P	H	.	.	.	.	.	.	.	G	.	.	.	.
MacaqueR11	.	.	.	.	.	.	.	.	.	P	.	P	H	.	.	.	.	.	.	.	G	.	.	.	.

Table S3. DNA/Protein Model comparison between 25 models including the number of parameters, AICc and lnL

Model	#Parameters	AICc	lnL
TN93+G+I	106	15792.76	-7790.15
TN93+G	105	15797.00	-7793.28
GTR+G+I	109	15797.15	-7789.33
GTR+G	108	15801.25	-7792.39
HKY+G+I	105	15803.62	-7796.58
HKY+G	104	15807.12	-7799.34
T92+G+I	103	15854.64	-7824.10
T92+G	102	15858.75	-7827.16
K2+G+I	102	15872.82	-7834.20
K2+G	101	15875.50	-7836.54
TN93+I	105	16001.73	-7895.64
GTR+I	108	16004.65	-7894.09
HKY+I	104	16011.18	-7901.37
T92+I	102	16062.56	-7929.07
K2+I	101	16073.61	-7935.60
JC+G+I	101	16259.30	-8028.44
JC+G	100	16261.94	-8030.77
JC+I	100	16455.10	-8127.35
GTR	107	16547.98	-8166.76
TN93	104	16561.66	-8176.61
HKY	103	16574.90	-8184.23
K2	100	16609.66	-8204.63
T92	101	16645.80	-8221.69
JC	99	16984.97	-8393.28

Table S4. The *K*a/*K*s ratio and (1 − *K*a/*K*s) × 100 for pairs of repeats of the filaggrin gene within five primate species.

**Human**					
**Repeat 1**	**Repeat 2**	***K*s**[a]	***K*a**[b]	***K*a/*K*s**[c]	**(1 − *K*a/*K*s) × 100**[d]
**HumanR10**	**HumanR3**	**0.068**	**0.071**	**1.049**	
**HumanR3**	**HumanR7**	**0.071**	**0.074**	**1.047**	
HumanR7	HumanR8	0.054	0.053	0.978	2.2
HumanR3	HumanR8	0.101	0.089	0.877	12.3
HumanR2	HumanR3	0.104	0.090	0.862	13.8
HumanR2	HumanR8	0.066	0.057	0.856	14.4
HumanR3	HumanR9	0.124	0.105	0.846	15.4
HumanR7	HumanR9	0.110	0.091	0.825	17.5
HumanR3	HumanR5	0.080	0.064	0.800	20
HumanR1	HumanR3	0.090	0.071	0.794	20.6
HumanR4	HumanR7	0.124	0.097	0.783	21.7
HumanR2	HumanR7	0.103	0.080	0.775	22.5
HumanR4	HumanR8	0.119	0.091	0.765	23.5
HumanR2	HumanR5	0.110	0.083	0.762	23.8
HumanR6	HumanR7	0.095	0.071	0.755	24.5
HumanR2	HumanR9	0.050	0.037	0.754	24.6
HumanR6	HumanR8	0.098	0.073	0.746	25.4
HumanR10	HumanR8	0.090	0.065	0.727	27.3
HumanR5	HumanR7	0.078	0.056	0.723	27.7
HumanR6	HumanR9	0.121	0.086	0.712	28.8
HumanR1	HumanR8	0.100	0.068	0.686	31.4
HumanR2	HumanR6	0.113	0.078	0.685	31.5
HumanR10	HumanR7	0.075	0.051	0.685	31.5
HumanR8	HumanR9	0.052	0.035	0.680	32
HumanR10	HumanR9	0.112	0.075	0.669	33.1
HumanR10	HumanR4	0.136	0.091	0.667	33.3
HumanR1	HumanR2	0.110	0.073	0.663	33.7
HumanR5	HumanR9	0.135	0.088	0.653	34.7
HumanR3	HumanR4	0.150	0.096	0.636	36.4
HumanR1	HumanR9	0.133	0.084	0.635	36.5

HumanR1	HumanR4	0.150	0.094	0.630	37
HumanR4	HumanR6	0.136	0.086	0.630	37
HumanR2	HumanR4	0.140	0.088	0.629	37.1
HumanR4	HumanR9	0.153	0.095	0.622	37.8
HumanR3	HumanR6	0.140	0.085	0.604	39.6
HumanR4	HumanR5	0.162	0.097	0.595	40.5
HumanR10	HumanR2	0.108	0.063	0.588	41.2
HumanR5	HumanR6	0.127	0.074	0.583	41.7
HumanR5	HumanR8	0.117	0.068	0.579	42.1
HumanR1	HumanR7	0.087	0.050	0.574	42.6
HumanR10	HumanR5	0.103	0.057	0.551	44.9
HumanR10	HumanR6	0.096	0.051	0.525	47.5
HumanR1	HumanR10	0.104	0.050	0.480	52
HumanR1	HumanR6	0.143	0.068	0.471	52.9
HumanR1	HumanR5	0.122	0.049	0.401	59.9

Chimpanzee

**Repeat 1**	**Repeat 2**	***K*s[a]**	***K*a[b]**	***K*a/*K*s[c]**	**(1 − *K*a/*K*s) × 100[d]**
**ChimpanzeeR3**	**ChimpanzeeR6**	**0.004**	**0.018**	**4.023**	
**ChimpanzeeR10**	**ChimpanzeeR3**	**0.079**	**0.079**	**1.010**	
ChimpanzeeR10	ChimpanzeeR8	0.070	0.066	0.937	6.3
ChimpanzeeR10	ChimpanzeeR2	0.115	0.091	0.792	20.8
ChimpanzeeR10	ChimpanzeeR5	0.115	0.091	0.792	20.8
ChimpanzeeR6	ChimpanzeeR8	0.100	0.076	0.758	24.2
ChimpanzeeR10	ChimpanzeeR6	0.083	0.063	0.758	24.2
ChimpanzeeR2	ChimpanzeeR7	0.145	0.107	0.736	26.4
ChimpanzeeR5	ChimpanzeeR7	0.145	0.107	0.736	26.4
ChimpanzeeR10	ChimpanzeeR9	0.098	0.071	0.722	27.8
ChimpanzeeR2	ChimpanzeeR8	0.148	0.103	0.695	30.5
ChimpanzeeR5	ChimpanzeeR8	0.148	0.103	0.695	30.5
ChimpanzeeR3	ChimpanzeeR8	0.095	0.065	0.686	31.4
ChimpanzeeR2	ChimpanzeeR3	0.148	0.099	0.673	32.7
ChimpanzeeR3	ChimpanzeeR5	0.148	0.099	0.673	32.7
ChimpanzeeR2	ChimpanzeeR6	0.137	0.092	0.670	33

ChimpanzeeR5	ChimpanzeeR6	0.137	0.092	0.670	33
ChimpanzeeR2	ChimpanzeeR9	0.136	0.091	0.669	33.1
ChimpanzeeR5	ChimpanzeeR9	0.136	0.091	0.669	33.1
ChimpanzeeR3	ChimpanzeeR7	0.093	0.062	0.666	33.4
ChimpanzeeR4	ChimpanzeeR7	0.082	0.053	0.649	35.1
ChimpanzeeR3	ChimpanzeeR9	0.092	0.059	0.646	35.4
ChimpanzeeR8	ChimpanzeeR9	0.115	0.071	0.622	37.8
ChimpanzeeR2	ChimpanzeeR4	0.146	0.089	0.608	39.2
ChimpanzeeR4	ChimpanzeeR5	0.146	0.089	0.608	39.2
ChimpanzeeR4	ChimpanzeeR9	0.073	0.044	0.607	39.3
ChimpanzeeR6	ChimpanzeeR7	0.088	0.052	0.590	41
ChimpanzeeR10	ChimpanzeeR7	0.113	0.066	0.584	41.6
ChimpanzeeR7	ChimpanzeeR9	0.101	0.058	0.571	42.9
ChimpanzeeR3	ChimpanzeeR4	0.107	0.060	0.566	43.4
ChimpanzeeR1	ChimpanzeeR2	0.163	0.089	0.544	45.6
ChimpanzeeR1	ChimpanzeeR5	0.163	0.089	0.544	45.6
ChimpanzeeR10	ChimpanzeeR4	0.112	0.060	0.537	46.3
ChimpanzeeR6	ChimpanzeeR9	0.091	0.049	0.537	46.3
ChimpanzeeR7	ChimpanzeeR8	0.146	0.076	0.519	48.1
ChimpanzeeR4	ChimpanzeeR8	0.140	0.072	0.511	48.9
ChimpanzeeR4	ChimpanzeeR6	0.102	0.050	0.495	50.5
ChimpanzeeR10	ChimpanzeeR1	0.134	0.066	0.495	50.5
ChimpanzeeR1	ChimpanzeeR8	0.143	0.069	0.482	51.8
ChimpanzeeR1	ChimpanzeeR9	0.091	0.040	0.443	55.7
ChimpanzeeR1	ChimpanzeeR7	0.112	0.047	0.422	57.8
ChimpanzeeR1	ChimpanzeeR3	0.133	0.055	0.410	59
ChimpanzeeR1	ChimpanzeeR6	0.128	0.044	0.348	65.2
ChimpanzeeR1	ChimpanzeeR4	0.068	0.022	0.324	67.6
ChimpanzeeR2	ChimpanzeeR5	0.000	0.000		
**Gorilla**					
**Repeat 1**	**Repeat 2**	***K*s[a]**	***K*a[b]**	***K*a/*K*s[c]**	**(1 − *K*a/*K*s) × 100[d]**
**GorillaR2**	**GorillaR8**	0.009	0.022	2.477	
**GorillaR2**	**GorillaR9**	0.063	0.071	1.117	
**GorillaR8**	**GorillaR9**	0.073	0.080	1.098	

**GorillaR7**	**GorillaR8**	0.090	0.097	1.075	
**GorillaR3**	**GorillaR8**	0.090	0.096	1.062	
**GorillaR1**	**GorillaR8**	0.088	0.093	1.057	
**GorillaR5**	**GorillaR8**	0.095	0.096	1.007	
**GorillaR2**	**GorillaR7**	0.090	0.091	1.003	
GorillaR2	GorillaR3	0.090	0.089	0.991	0.9
GorillaR4	GorillaR6	0.004	0.004	0.953	4.7
GorillaR1	GorillaR2	0.086	0.082	0.951	4.9
GorillaR2	GorillaR5	0.095	0.089	0.940	6
GorillaR4	GorillaR8	0.085	0.070	0.826	17.4
GorillaR6	GorillaR7	0.084	0.069	0.822	17.8
GorillaR6	GorillaR8	0.087	0.071	0.811	18.9
GorillaR4	GorillaR7	0.081	0.065	0.802	19.8
GorillaR3	GorillaR9	0.051	0.041	0.801	19.9
GorillaR1	GorillaR7	0.082	0.065	0.791	20.9
GorillaR3	GorillaR4	0.083	0.066	0.790	21
GorillaR3	GorillaR6	0.086	0.067	0.775	22.5
GorillaR7	GorillaR9	0.061	0.045	0.748	25.2
GorillaR4	GorillaR5	0.088	0.066	0.747	25.3
GorillaR1	GorillaR3	0.084	0.063	0.746	25.4
GorillaR2	GorillaR4	0.083	0.062	0.739	26.1
GorillaR2	GorillaR6	0.085	0.063	0.735	26.5
GorillaR5	GorillaR9	0.056	0.041	0.735	26.5
GorillaR5	GorillaR6	0.091	0.067	0.734	26.6
GorillaR1	GorillaR8	0.128	0.092	0.717	28.3
GorillaR1	GorillaR5	0.089	0.063	0.705	29.5
GorillaR1	GorillaR2	0.115	0.080	0.696	30.4
GorillaR1	GorillaR9	0.086	0.059	0.686	31.4
GorillaR4	GorillaR9	0.100	0.067	0.667	33.3
GorillaR6	GorillaR9	0.103	0.068	0.659	34.1
GorillaR1	GorillaR7	0.109	0.066	0.604	39.6
GorillaR1	GorillaR3	0.101	0.061	0.602	39.8
GorillaR1	GorillaR4	0.078	0.045	0.574	42.6
GorillaR1	GorillaR5	0.106	0.061	0.573	42.7
GorillaR1	GorillaR9	0.103	0.059	0.569	43.1
GorillaR1	GorillaR6	0.082	0.046	0.557	44.3

GorillaR1	GorillaR6	0.072	0.036	0.497	50.3
GorillaR3	GorillaR7	0.009	0.004	0.471	52.9
GorillaR1	GorillaR1	0.085	0.039	0.462	53.8
GorillaR1	GorillaR4	0.077	0.035	0.449	55.1
GorillaR5	GorillaR7	0.013	0.004	0.313	68.7
GorillaR3	GorillaR5	0.004	0.000	0.000	
**Orangutan**					
**Repeat 1**	**Repeat 2**	***Ks*** [a]	***Ka*** [b]	***Ka*/*Ks*** [c]	**(1 − *Ka*/*Ks*) × 100** [d]
OrangutanR2	OrangutanR7	0.085	0.080	0.943	5.7
OrangutanR4	OrangutanR7	0.052	0.047	0.909	9.1
OrangutanR5	OrangutanR7	0.054	0.046	0.855	14.5
OrangutanR2	OrangutanR3	0.097	0.079	0.815	18.5
OrangutanR1	OrangutanR2	0.148	0.117	0.788	21.2
OrangutanR2	OrangutanR6	0.102	0.081	0.788	21.2
OrangutanR7	OrangutanR9	0.073	0.055	0.763	23.7
OrangutanR6	OrangutanR7	0.078	0.060	0.759	24.1
OrangutanR2	OrangutanR5	0.100	0.075	0.758	24.2
OrangutanR5	OrangutanR6	0.106	0.080	0.754	24.6
OrangutanR4	OrangutanR9	0.086	0.065	0.753	24.7
OrangutanR4	OrangutanR6	0.093	0.070	0.752	24.8
OrangutanR6	OrangutanR8	0.119	0.086	0.729	27.1
OrangutanR3	OrangutanR8	0.101	0.071	0.702	29.8
OrangutanR1	OrangutanR5	0.140	0.097	0.695	30.5
OrangutanR5	OrangutanR9	0.104	0.070	0.674	32.6
OrangutanR4	OrangutanR5	0.043	0.029	0.671	32.9
OrangutanR3	OrangutanR5	0.110	0.074	0.668	33.2
OrangutanR2	OrangutanR4	0.107	0.070	0.659	34.1
OrangutanR2	OrangutanR8	0.115	0.075	0.657	34.3
OrangutanR8	OrangutanR9	0.128	0.082	0.641	35.9
OrangutanR6	OrangutanR9	0.095	0.060	0.635	36.5
OrangutanR3	OrangutanR9	0.096	0.059	0.610	39
OrangutanR3	OrangutanR7	0.100	0.060	0.601	39.9
OrangutanR1	OrangutanR6	0.162	0.097	0.599	40.1
OrangutanR1	OrangutanR4	0.147	0.087	0.592	40.8

OrangutanR2	OrangutanR9	0.120	0.071	0.587	41.3
OrangutanR3	OrangutanR4	0.103	0.060	0.581	41.9
OrangutanR7	OrangutanR8	0.108	0.063	0.579	42.1
OrangutanR1	OrangutanR7	0.159	0.090	0.569	43.1
OrangutanR5	OrangutanR8	0.121	0.066	0.549	45.1
OrangutanR3	OrangutanR6	0.093	0.049	0.528	47.2
OrangutanR1	OrangutanR9	0.192	0.101	0.526	47.4
OrangutanR4	OrangutanR8	0.113	0.054	0.476	52.4
OrangutanR1	OrangutanR3	0.198	0.089	0.448	55.2
OrangutanR1	OrangutanR8	0.198	0.087	0.440	56

Crab-eating macaque

**Repeat 1**	**Repeat 2**	***Ks***[a]	***Ka***[b]	***Ka*/*Ks***[c]	**(1 − *Ka*/*Ks*) × 100**[d]
Crab-eating macaqueR1	Crab-eating macaqueR1	0.049	0.026	0.530	47
Crab-eating macaqueR1	Crab-eating macaqueR4	0.049	0.026	0.530	47
Crab-eating macaqueR1	Crab-eating macaqueR6	0.049	0.026	0.530	47
Crab-eating macaqueR1	Crab-eating macaqueR8	0.049	0.026	0.530	47
Crab-eating macaqueR1	Crab-eating macaqueR1	0.035	0.015	0.441	55.9
Crab-eating macaqueR1	Crab-eating macaqueR1	0.086	0.033	0.388	61.2
Crab-eating macaqueR1	Crab-eating macaqueR4	0.086	0.033	0.388	61.2
Crab-eating macaqueR1	Crab-eating macaqueR6	0.086	0.033	0.388	61.2
Crab-eating macaqueR1	Crab-eating macaqueR8	0.086	0.033	0.388	61.2
Crab-eating macaqueR1	Crab-eating macaqueR2	0.087	0.029	0.332	66.8
Crab-eating macaqueR1	Crab-eating macaqueR1	0.071	0.021	0.291	70.9

Crab-eating macaqueR1	Crab-eating macaqueR3	0.071	0.021	0.291	70.9
Crab-eating macaqueR1	Crab-eating macaqueR5	0.071	0.021	0.291	70.9
Crab-eating macaqueR1	Crab-eating macaqueR7	0.071	0.021	0.291	70.9
Crab-eating macaqueR1	Crab-eating macaqueR9	0.071	0.021	0.291	70.9
Crab-eating macaqueR1	Crab-eating macaqueR1	0.126	0.036	0.288	71.2
Crab-eating macaqueR1	Crab-eating macaqueR3	0.126	0.036	0.288	71.2
Crab-eating macaqueR1	Crab-eating macaqueR5	0.126	0.036	0.288	71.2
Crab-eating macaqueR1	Crab-eating macaqueR7	0.126	0.036	0.288	71.2
Crab-eating macaqueR1	Crab-eating macaqueR9	0.126	0.036	0.288	71.2
Crab-eating macaqueR1	Crab-eating macaqueR4	0.126	0.036	0.288	71.2
Crab-eating macaqueR1	Crab-eating macaqueR6	0.126	0.036	0.288	71.2
Crab-eating macaqueR1	Crab-eating macaqueR8	0.126	0.036	0.288	71.2
Crab-eating macaqueR3	Crab-eating macaqueR4	0.126	0.036	0.288	71.2
Crab-eating macaqueR3	Crab-eating macaqueR6	0.126	0.036	0.288	71.2
Crab-eating macaqueR3	Crab-eating macaqueR8	0.126	0.036	0.288	71.2
Crab-eating macaqueR4	Crab-eating macaqueR5	0.126	0.036	0.288	71.2
Crab-eating macaqueR4	Crab-eating macaqueR7	0.126	0.036	0.288	71.2
Crab-eating macaqueR4	Crab-eating macaqueR9	0.126	0.036	0.288	71.2

Crab-eating macaqueR5	Crab-eating macaqueR6	0.126	0.036	0.288	71.2
Crab-eating macaqueR5	Crab-eating macaqueR8	0.126	0.036	0.288	71.2
Crab-eating macaqueR6	Crab-eating macaqueR7	0.126	0.036	0.288	71.2
Crab-eating macaqueR6	Crab-eating macaqueR9	0.126	0.036	0.288	71.2
Crab-eating macaqueR7	Crab-eating macaqueR8	0.126	0.036	0.288	71.2
Crab-eating macaqueR8	Crab-eating macaqueR9	0.126	0.036	0.288	71.2
Crab-eating macaqueR1	Crab-eating macaqueR2	0.126	0.036	0.288	71.2
Crab-eating macaqueR1	Crab-eating macaqueR1	0.035	0.008	0.234	76.6
Crab-eating macaqueR1	Crab-eating macaqueR3	0.035	0.008	0.234	76.6
Crab-eating macaqueR1	Crab-eating macaqueR5	0.035	0.008	0.234	76.6
Crab-eating macaqueR1	Crab-eating macaqueR7	0.035	0.008	0.234	76.6
Crab-eating macaqueR1	Crab-eating macaqueR9	0.035	0.008	0.234	76.6
Crab-eating macaqueR1	Crab-eating macaqueR2	0.168	0.039	0.233	76.7
Crab-eating macaqueR2	Crab-eating macaqueR3	0.168	0.039	0.233	76.7
Crab-eating macaqueR2	Crab-eating macaqueR5	0.168	0.039	0.233	76.7
Crab-eating macaqueR2	Crab-eating macaqueR7	0.168	0.039	0.233	76.7
Crab-eating macaqueR2	Crab-eating macaqueR9	0.168	0.039	0.233	76.7
Crab-eating macaqueR1	Crab-eating macaqueR2	0.045	0.003	0.060	94

Crab-eating macaqueR2	Crab-eating macaqueR4	0.045	0.003	0.060	94
Crab-eating macaqueR2	Crab-eating macaqueR6	0.045	0.003	0.060	94
Crab-eating macaqueR2	Crab-eating macaqueR8	0.045	0.003	0.060	94
Crab-eating macaqueR1	Crab-eating macaqueR4	0	0		
Crab-eating macaqueR1	Crab-eating macaqueR6	0	0		
Crab-eating macaqueR1	Crab-eating macaqueR8	0	0		
Crab-eating macaqueR1	Crab-eating macaqueR3	0	0		
Crab-eating macaqueR1	Crab-eating macaqueR5	0	0		
Crab-eating macaqueR1	Crab-eating macaqueR7	0	0		
Crab-eating macaqueR1	Crab-eating macaqueR9	0	0		
Crab-eating macaqueR3	Crab-eating macaqueR5	0	0		
Crab-eating macaqueR3	Crab-eating macaqueR7	0	0		
Crab-eating macaqueR3	Crab-eating macaqueR9	0	0		
Crab-eating macaqueR4	Crab-eating macaqueR6	0	0		
Crab-eating macaqueR4	Crab-eating macaqueR8	0	0		
Crab-eating macaqueR5	Crab-eating macaqueR7	0	0		
Crab-eating macaqueR5	Crab-eating macaqueR9	0	0		
Crab-eating macaqueR6	Crab-eating macaqueR8	0	0		

Crab-eating macaqueR7	Crab-eating macaqueR9	0	0

[a]*K*s refers to the number of synonymous nucleotide substitutions per total number of synonymous sites for each codon.

[b]*K*a refers to the number of nonsynonymous nucleotide substitutions per total number of nonsynonymous sites for each codon.

[c]*K*a/*K*s > 1 (bold) suggests positive selection.

[d]Purifying selection is measured by (1 − *K*a/*K*s) × 100.

Table S5. Recombination and gene conversion events in the filaggrin gene in primates, as detected by GeneConv.

	**Breakpoint positions**				**Detection methods**					
**Recombination event number**	**Begin**	**End**	**Recombinant sequence**	**Major parental sequence**	**RDP**	**GENECONV**	**Bootscan**	**Maxchi**	**Chimaera**	**SiSscan**
				**Crab-eating macaque**						
1	656	735	Crab-eating macaque-R1	Crab-eating macaque-R12	NS	7.75E-03	NS	2.63E-04	7.46E-04	NS
2	1	266	Crab-eating macaque-R2	Crab-eating macaque-R11	NS	6.05E-03	4.08E-03	NS	NS	NS
				**Orangutan**						
1	651	972	Orangutan-R7	Orangutan-R4	8.95E-05	2.36E-04	1.27E-04	1.32E-04	3.66E-04	1.04E-04
				**Gorilla**						
1	575	978	Gorilla-R8	Gorilla-R10	NS	NS	NS	1.69E-04	1.25E-03	1.72E-05
2	245	594	Gorilla-R9	Gorilla-R4	NS	NS	6.75E-08	3.85E-04	NS	4.92E-08
				**Chimpanzee**						
1	172	975	Chimpanzee-R6	Chimpanzee-R3	NS	NS	4.16E-03	1.70E-03	2.93E-03	NS
				**Human**						
1	534	975	Human-R8	Human-R9	NS	5.85E-09	5.25E-11	3.59E-09	4.89E-10	8.55E-12
2	504	975	Human-R10	Human-R3	NS	2.66E-03	5.50E-08	3.39E-06	2.62E-07	9.72E-06

Table S6. Comparison between filaggrin repeat units and filaggrin-like repeat units from macaque.

Species	No. of repeats	Total no. variation sites	Average (1 − Ka/Ks) x 100[a]	Nucleotide variation
**CRAB-EATING MACAQUE**				
**Crab-eating macaque FLG**	12	69	70.96	0.032
**Crab-eating macaque FLG-like**	5	73	71.33	0.039

[a] (1-Ka/Ks) x 100 measures the percentage of purifying selection.

Table S7. The *K*a/*K*s ratio and (1 − *K*a/*K*s) × 100 for pairs of repeats of the filaggrin-like gene within macaque.

Crab-eating macaque FLG-like

Repeat 1	Repeat 2	Ks	Ka	Ka/Ks	(1 − *K*a/*K*s) x 100[d]
Macaque-like1	Macaque-like2	0.031	0.016	0.53	47
Macaque-like1	Macaque-like3	0.144	0.033	0.23	77
Macaque-like1	Macaque-like4	0.108	0.024	0.22	78
Macaque-like1	Macaque-like5	0.085	0.016	0.19	81
Macaque-like2	Macaque-like3	0.17	0.044	0.26	74
Macaque-like2	Macaque-like4	0.133	0.038	0.29	71
Macaque-like2	Macaque-like5	0.099	0.030	0.30	70
Macaque-like3	Macaque-like4	0.035	0.011	0.31	69
Macaque-like3	Macaque-like5	0.076	0.019	0.25	75
Macaque-like4	Macaque-like5	0.048	0.014	0.29	71

[a]*K*s refers to the number of synonymous nucleotide substitutions per total number of synonymous sites for each codon.

[b]*K*a refers to the number of nonsynonymous nucleotide substitutions per total number of nonsynonymous sites for each codon.

[c]*K*a/*K*s > 1 (bold) suggests positive selection.

[d]Purifying selection is measured by (1 − *K*a/*K*s) × 100.

Table S8. Positively selected codons of the filaggrin-like in macaque.

Crab-eating macaque FLG-like

Sites-based test

**Models compared**	***P* value**	**Positively selected codons**
M1a vs. M2a[a]	0.99	No sites
M7 vs M8 [a]	0.99	No sites

[a]The site-based test compared the M1a(nearly neutral) and M2a (positive selection) models and the M7 (Beta) and M8 (Beta and ω)models.

Figure S1. Diagram of filaggrin gene structure and repeated region. Human filaggrin gene includes 3 exons and 2 introns, with the repeat region being found on the third. The rest of the human filaggrin gene comprises exon 1 with 54bp, intron 1 with 9613bp, exon 2 with 159bp, an intron 2 with 570bp and, in exon 3 a Domain A, which contains two calcium-binding domain, and Domain B, which facilitates the translocation in terminally differentiating keratinocytes, with 879bp, for a total of 11275bp which we further refer this region as the "non-repeated region". Neither exons other than exon 3 nor introns have filaggrin repeated units. The filaggrin repeat region consists of complete repeats that are flanked by two partial repeats. Black = partial repeats and white = complete repeats. UTR = Untranslated region.

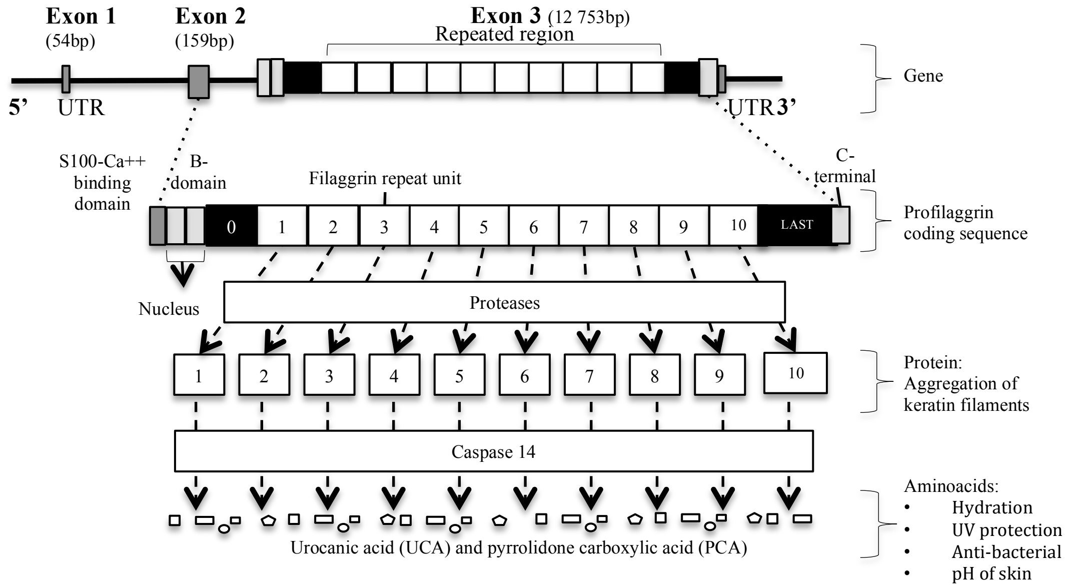

Figure S2. Dot-plot matrices between the human repeat region (*x*-axis) and primate exon 3 (*y*-axis) sequences of the filaggrin gene downloaded from the National Center for Biotechnology Information database. (**A**) Dot-plot matrix between the human repeat region and orangutan exon 3 sequences, compared using the nucleotide method with a 5-unit size filter option; misalignment regions were found between 5,200 and 9,100 bp, as shown in Figure 1. (**B**) Dot-plot matrix between the human repeat region and gorilla exon 3 sequences, using the nucleotide method with a 5-unit size filter option; misalignment regions (shown as red lines) were found at the end of the gorilla sequence after 9,000 bp, as shown in Figure 1. (**C**) Dot-plot matrix between the chimpanzee FLG-like sequence and chimpanzee-repeat 1, using the nucleotide method with a 5-unit size filter option; filaggrin repeat units are shown as yellow lines. Filaggrin-like gene in chimpanzee includes 1 complete repeat between two incomplete repeats. (**D**) Dot-plot matrix between the crab-eating macaque FLG-like sequence located in chromosome 1 and macaque-repeat 1, using the nucleotide method with a 5-unit size filter option; filaggrin repeat units are shown as purple lines. Filaggrin-like gene in macaque located in chromosome 1 includes 5 complete filaggrin repeats. (**E**) Dot-plot matrix between the crab-eating macaque FLG-like sequence located in chromosome 3 and macaque-repeat 1, using the nucleotide method with a 5-unit size filter option; none filaggrin repeat units were found.

A.

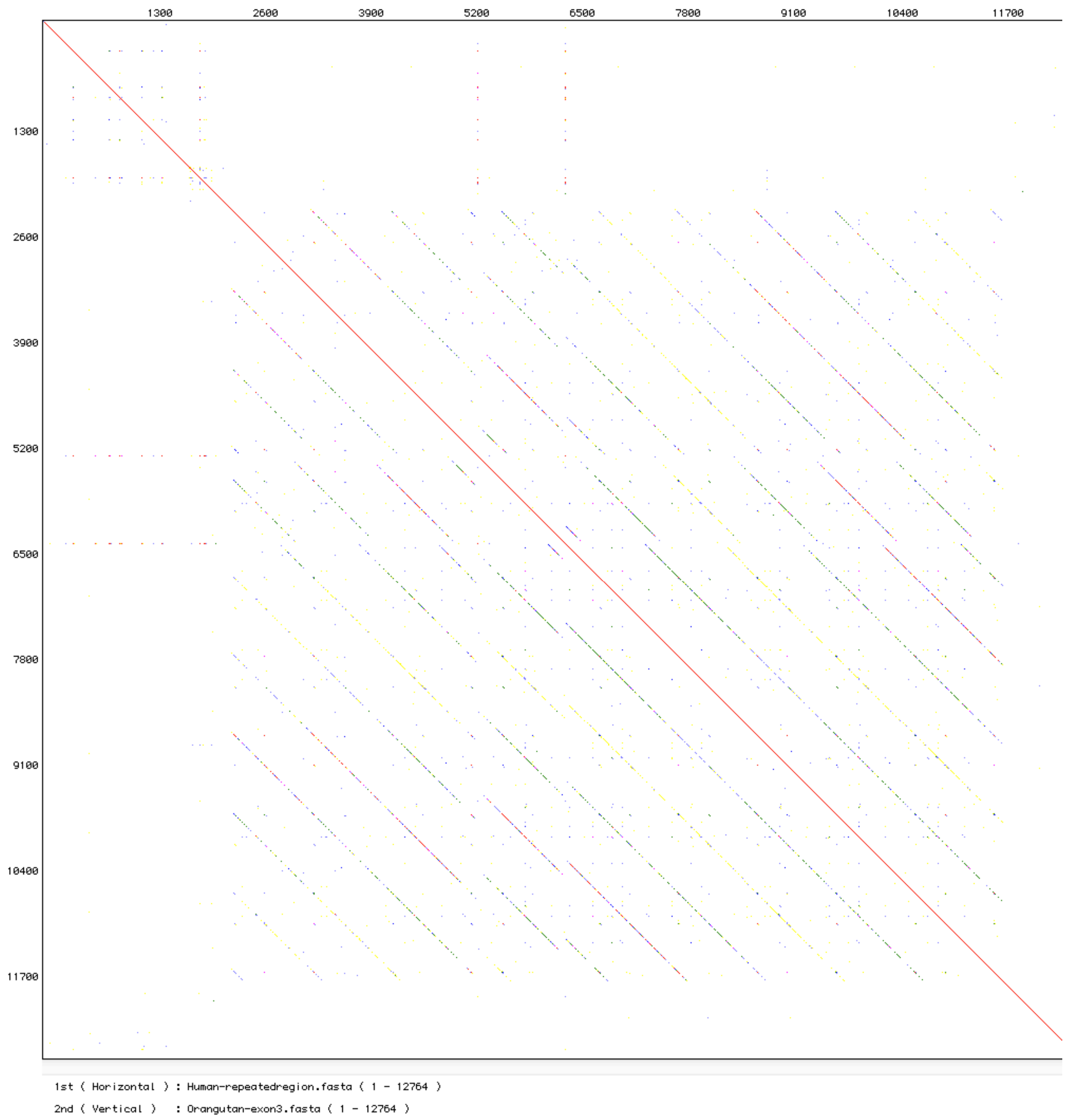
1300
2600
3900
5200
6500
7800
9100
10400
11700
1st (Horizontal) : Human-repeatedregion.fasta (1 - 12764)
2nd (Vertical) : Orangutan-exon3.fasta (1 - 12764)

B

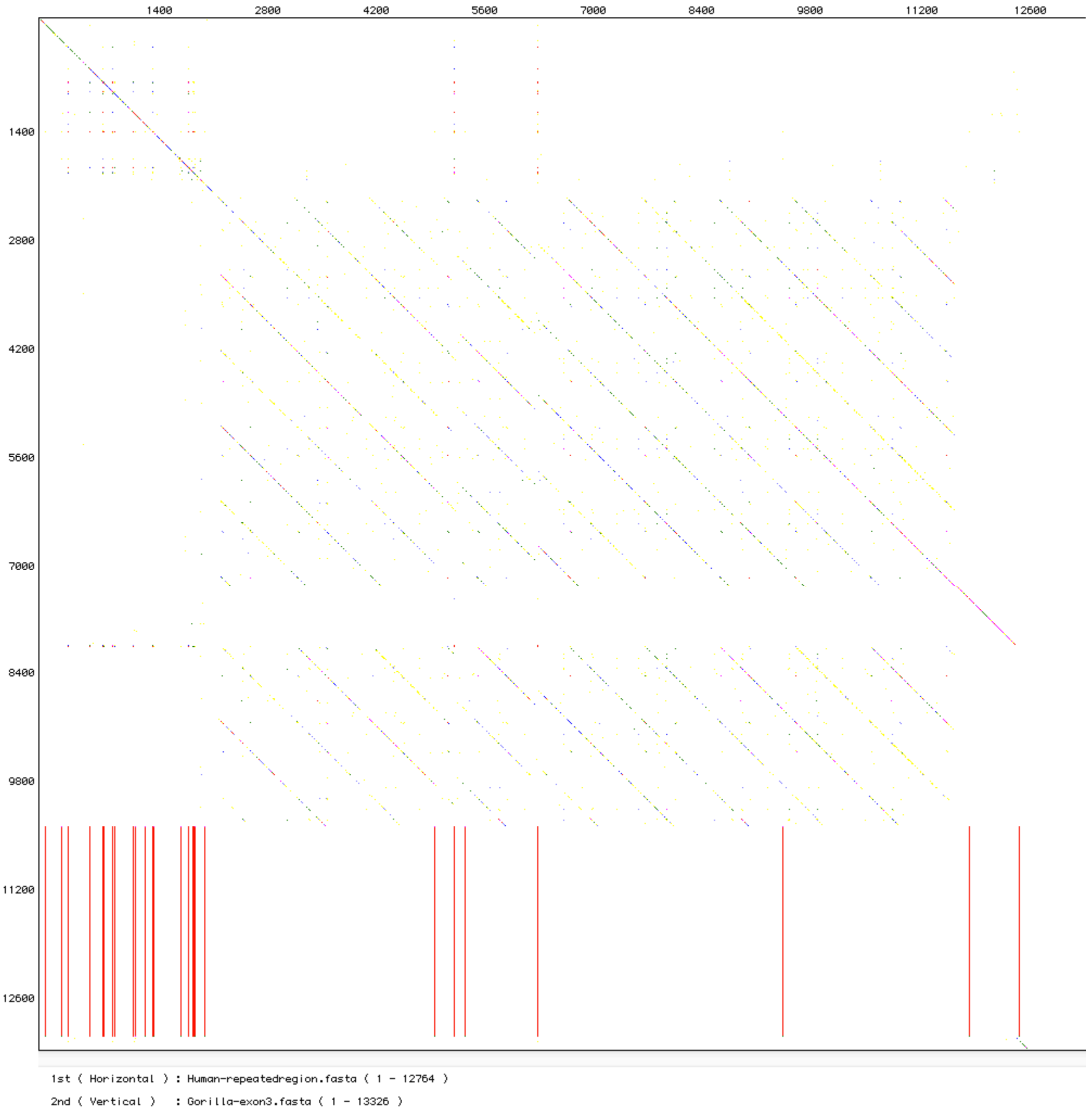
1400
2800
4200
5600
7000
8400
9800
11200
12600
1st (Horizontal) : Human-repeatedregion.fasta (1 - 12764)
2nd (Vertical) : Gorilla-exon3.fasta (1 - 13326)

C

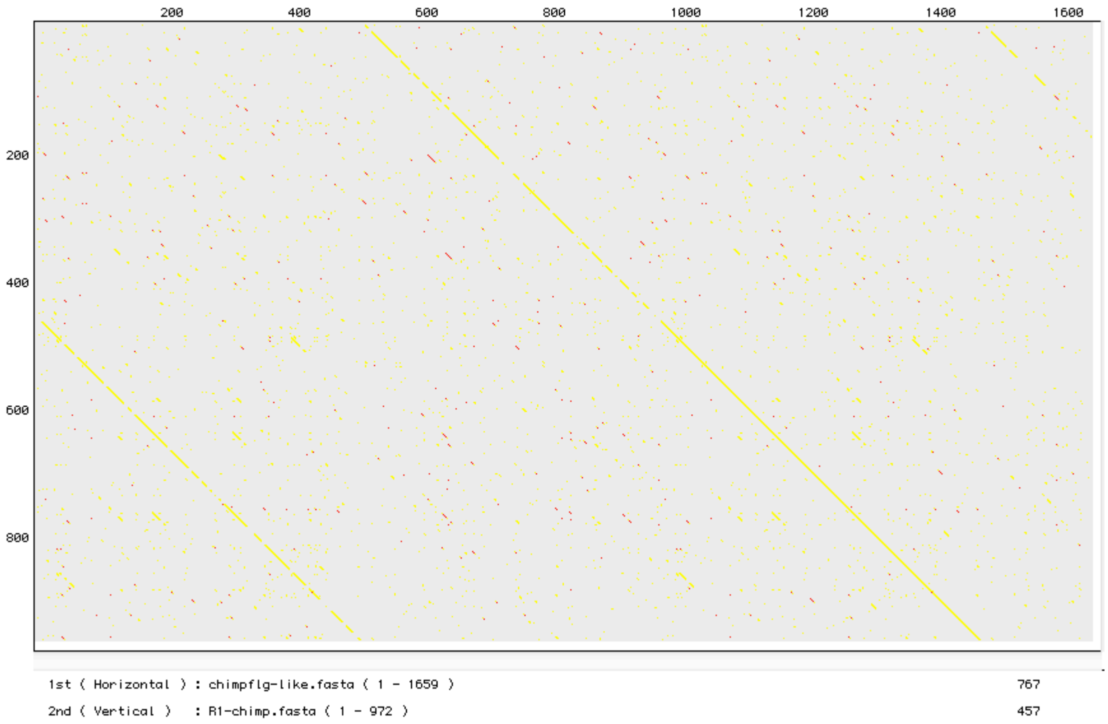
200
400
600
800
1000
1200
1400
1600
200
400
600
800
1st (Horizontal) : chimpflg-like.fasta (1 - 1659) 767
2nd (Vertical) : R1-chimp.fasta (1 - 972) 457

D

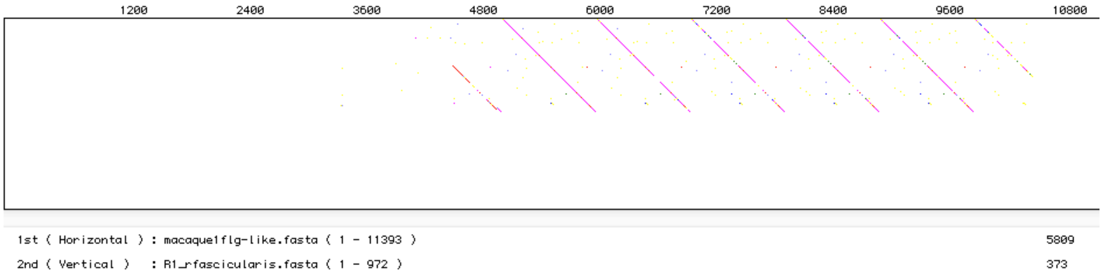
1200
2400
3600
4800
6000
7200
8400
9600
10800
1st (Horizontal) : macaque1flg-like.fasta (1 - 11393)
5809
2nd (Vertical) : R1_rfascicularis.fasta (1 - 972)
373

E

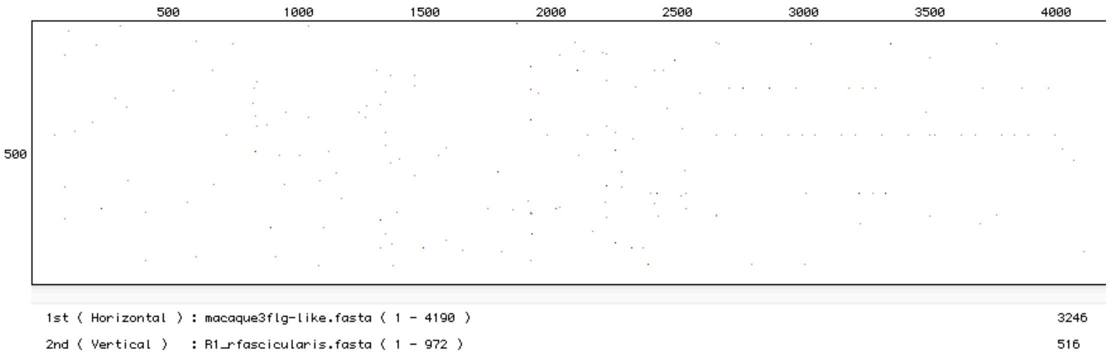
500
1000
1500
2000
2500
3000
3500
4000
500
1st (Horizontal) : macaque3flg-like.fasta (1 - 4190)
3246
2nd (Vertical) : R1_rfascicularis.fasta (1 - 972)
516

Figure S3. Phylogenetic tree reconstructions using all complete repeats of the filaggrin gene in human, chimpanzee, gorilla, orangutan, and crab-eating macaque. (**A**) Neighbor-joining-tree reconstruction using all complete repeats of *FLG* in human, chimpanzee, gorilla, orangutan, and crab-eating macaque. The following parameters were used: pairwise deletion, proportional nucleotide differences, 1,000 bootstrap resampling, and a cutoff value of 50%. Bootstrap values are shown at the beginning of each branch. (**B**) Maximum parsimony tree reconstruction using all complete repeats in these primates and the following parameters: partial deletion, and a 1,000 bootstrap resampling.

A.

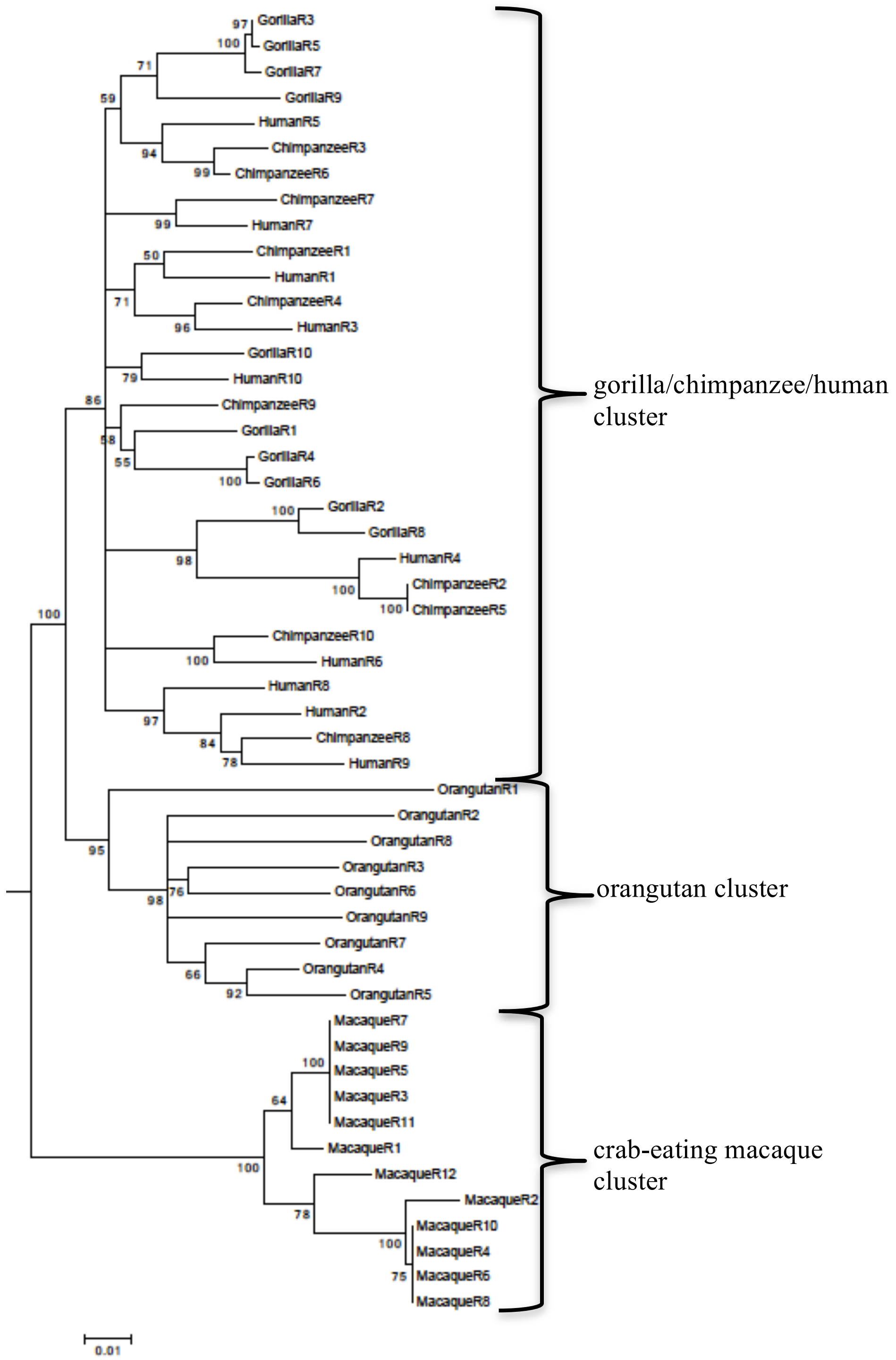
GorillaR3
GorillaR5
GorillaR7
GorillaR9
HumanR5
ChimpanzeeR3
ChimpanzeeR6
ChimpanzeeR7
HumanR7
ChimpanzeeR1
HumanR1
ChimpanzeeR4
HumanR3
GorillaR10
HumanR10
ChimpanzeeR9
GorillaR1
GorillaR4
GorillaR6
GorillaR2
GorillaR8
HumanR4
ChimpanzeeR2
ChimpanzeeR5
ChimpanzeeR10
HumanR6
HumanR8
HumanR2
ChimpanzeeR8
HumanR9
OrangutanR1
OrangutanR2
OrangutanR8
OrangutanR3
OrangutanR6
OrangutanR9
OrangutanR7
OrangutanR4
OrangutanR5
MacaqueR7
MacaqueR9
MacaqueR5
MacaqueR3
MacaqueR11
MacaqueR1
MacaqueR12
MacaqueR2
MacaqueR10
MacaqueR4
MacaqueR6
MacaqueR8
0.01
gorilla/chimpanzee/human cluster
orangutan cluster
crab-eating macaque cluster

B.

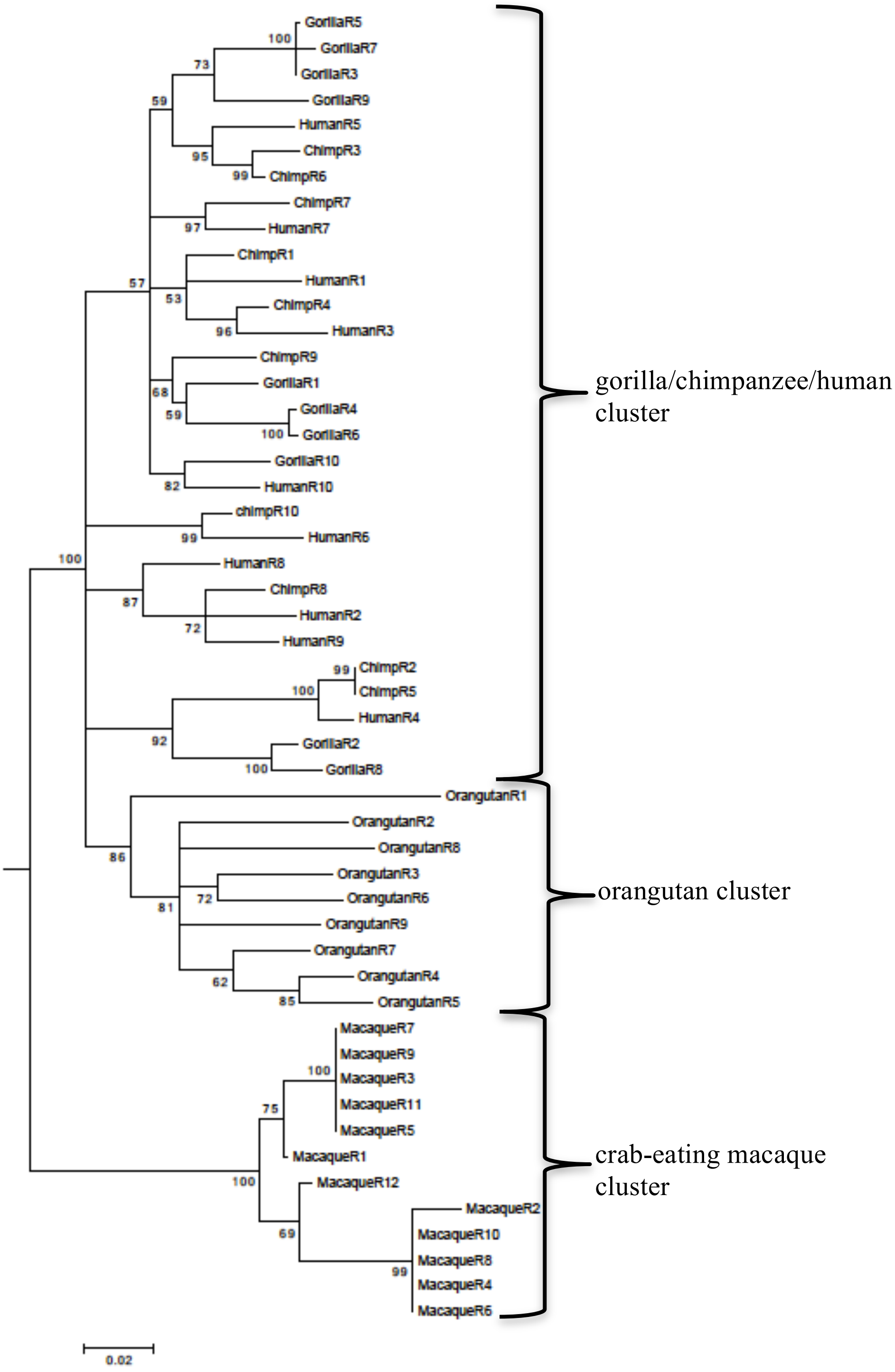
GorillaR5
GorillaR7
GorillaR3
GorillaR9
HumanR5
ChimpR3
ChimpR6
ChimpR7
HumanR7
ChimpR1
HumanR1
ChimpR4
HumanR3
ChimpR9
GorillaR1
GorillaR4
GorillaR6
GorillaR10
HumanR10
chimpR10
HumanR6
HumanR8
ChimpR8
HumanR2
HumanR9
ChimpR2
ChimpR5
HumanR4
GorillaR2
GorillaR8
OrangutanR1
OrangutanR2
OrangutanR8
OrangutanR3
OrangutanR6
OrangutanR9
OrangutanR7
OrangutanR4
OrangutanR5
MacaqueR7
MacaqueR9
MacaqueR3
MacaqueR11
MacaqueR5
MacaqueR1
MacaqueR12
MacaqueR2
MacaqueR10
MacaqueR8
MacaqueR4
MacaqueR6
gorilla/chimpanzee/human cluster
orangutan cluster
crab-eating macaque cluster
0.02

Figure S4. Phylogenetic tree reconstructions using all complete repeats of the filaggrin gene in human, chimpanzee, gorilla, orangutan, crab-eating macaque, dog and mouse. (**A**) Maximum likelihood tree reconstruction using all complete repeats in human, chimpanzee, gorilla, orangutan, crab-eating macaque, dog, and mouse and the following parameters: partial deletion option, Tamura-Nei model with gamma distribution and invariable sites, nearest-neighbor-interchange heuristic method, 1,000 bootstrap resampling, and a cutoff value of 50%. (**B**) Neighbor-joining-tree reconstruction using all complete repeats of *FLG* in human, chimpanzee, gorilla, orangutan, crab-eating macaque, dog, and mouse. The following parameters were used: pairwise deletion, proportional nucleotide differences, 1,000 bootstrap resampling, and a cutoff value of 50%. Bootstrap values are shown at the beginning of each branch. (**C**) Maximum parsimony tree reconstruction using all complete repeats in human, chimpanzee, gorilla, orangutan, crab-eating macaque, dog, and mouse and the following parameters: partial deletion, and a 1,000 bootstrap resampling. (**D**) A "Reconciled" tree using the complete repeats of the filaggrin gene in human, chimpanzee, gorilla, orangutan, crab-eating macaque, dog, and mouse. This "reconciled" tree was able to detect duplication (red squares) and loss events (light gray italics) from the most common ancestor of these species. Mouse repeats duplicated 15 times, dog repeats duplicated 3 times, crab-eating macaque repeats duplicated 11 times, orangutan repeats duplicated 8 times, and gorilla/chimpanzee/human repeats duplicated 18 times. In human, the counterpart to chimpanzee-repeat 9 has been lost, while in chimpanzee; the counterparts to human-repeats 10 and 8 have been lost.

A.

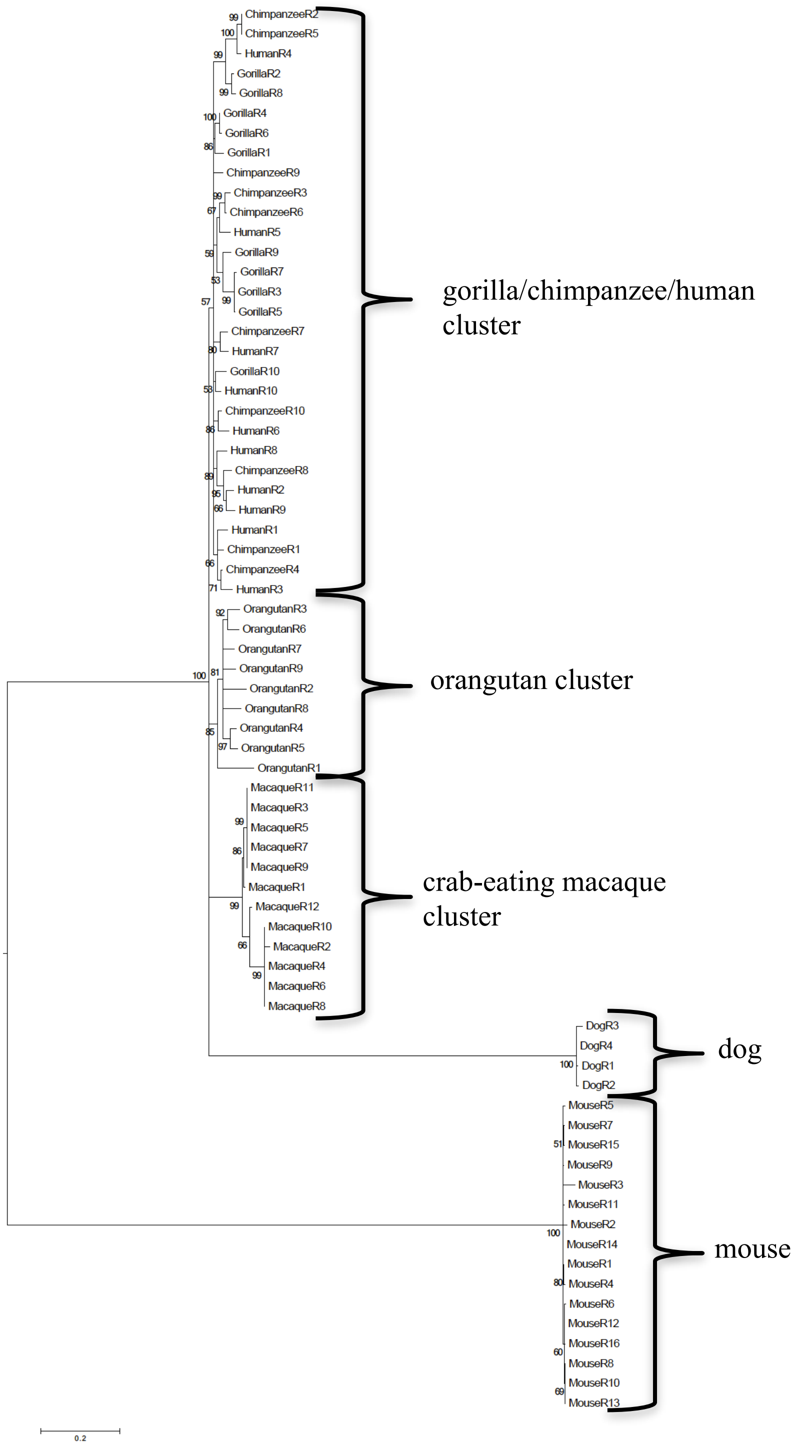

ChimpanzeeR2
ChimpanzeeR5
HumanR4
GorillaR2
GorillaR8
GorillaR4
GorillaR6
GorillaR1
ChimpanzeeR9
ChimpanzeeR3
ChimpanzeeR6
HumanR5
GorillaR9
GorillaR7
GorillaR3
GorillaR5
ChimpanzeeR7
HumanR7
GorillaR10
HumanR10
ChimpanzeeR10
HumanR6
HumanR8
ChimpanzeeR8
HumanR2
HumanR9
HumanR1
ChimpanzeeR1
ChimpanzeeR4
HumanR3
OrangutanR3
OrangutanR6
OrangutanR7
OrangutanR9
OrangutanR2
OrangutanR8
OrangutanR4
OrangutanR5
OrangutanR1
MacaqueR11
MacaqueR3
MacaqueR5
MacaqueR7
MacaqueR9
MacaqueR1
MacaqueR12
MacaqueR10
MacaqueR2
MacaqueR4
MacaqueR6
MacaqueR8
DogR3
DogR4
DogR1
DogR2
MouseR5
MouseR7
MouseR15
MouseR9
MouseR3
MouseR11
MouseR2
MouseR14
MouseR1
MouseR4
MouseR6
MouseR12
MouseR16
MouseR8
MouseR10
MouseR13
gorilla/chimpanzee/human cluster
orangutan cluster
crab-eating macaque cluster
dog
mouse
0.2

B.

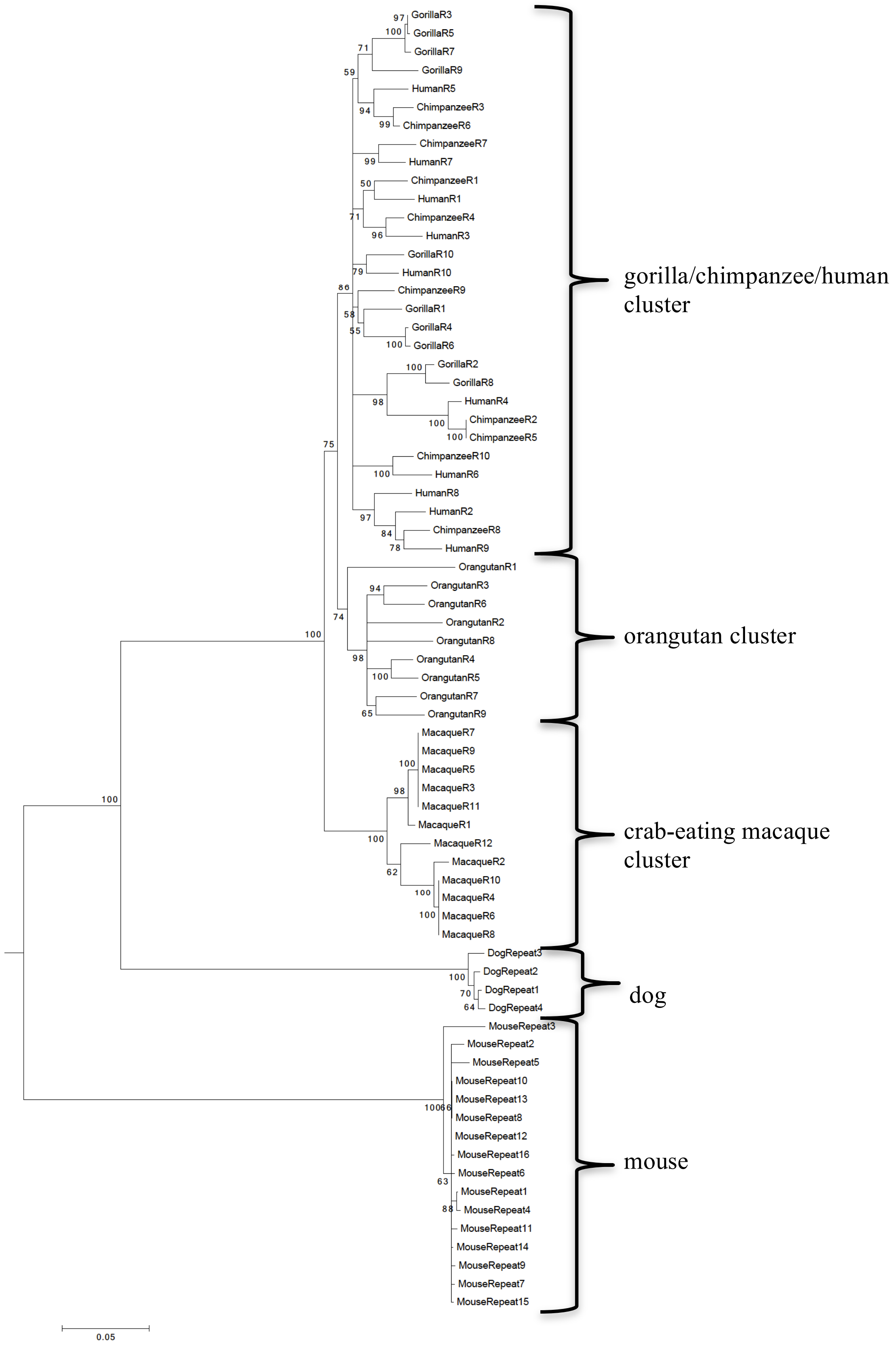
GorillaR3
GorillaR5
GorillaR7
GorillaR9
HumanR5
ChimpanzeeR3
ChimpanzeeR6
ChimpanzeeR7
HumanR7
ChimpanzeeR1
HumanR1
ChimpanzeeR4
HumanR3
GorillaR10
HumanR10
ChimpanzeeR9
GorillaR1
GorillaR4
GorillaR6
GorillaR2
GorillaR8
HumanR4
ChimpanzeeR2
ChimpanzeeR5
ChimpanzeeR10
HumanR6
HumanR8
HumanR2
ChimpanzeeR8
HumanR9
OrangutanR1
OrangutanR3
OrangutanR6
OrangutanR2
OrangutanR8
OrangutanR4
OrangutanR5
OrangutanR7
OrangutanR9
MacaqueR7
MacaqueR9
MacaqueR5
MacaqueR3
MacaqueR11
MacaqueR1
MacaqueR12
MacaqueR2
MacaqueR10
MacaqueR4
MacaqueR6
MacaqueR8
DogRepeat3
DogRepeat2
DogRepeat1
DogRepeat4
MouseRepeat3
MouseRepeat2
MouseRepeat5
MouseRepeat10
MouseRepeat13
MouseRepeat8
MouseRepeat12
MouseRepeat16
MouseRepeat6
MouseRepeat1
MouseRepeat4
MouseRepeat11
MouseRepeat14
MouseRepeat9
MouseRepeat7
MouseRepeat15
gorilla/chimpanzee/human cluster
orangutan cluster
crab-eating macaque cluster
dog
mouse
0.05

C.

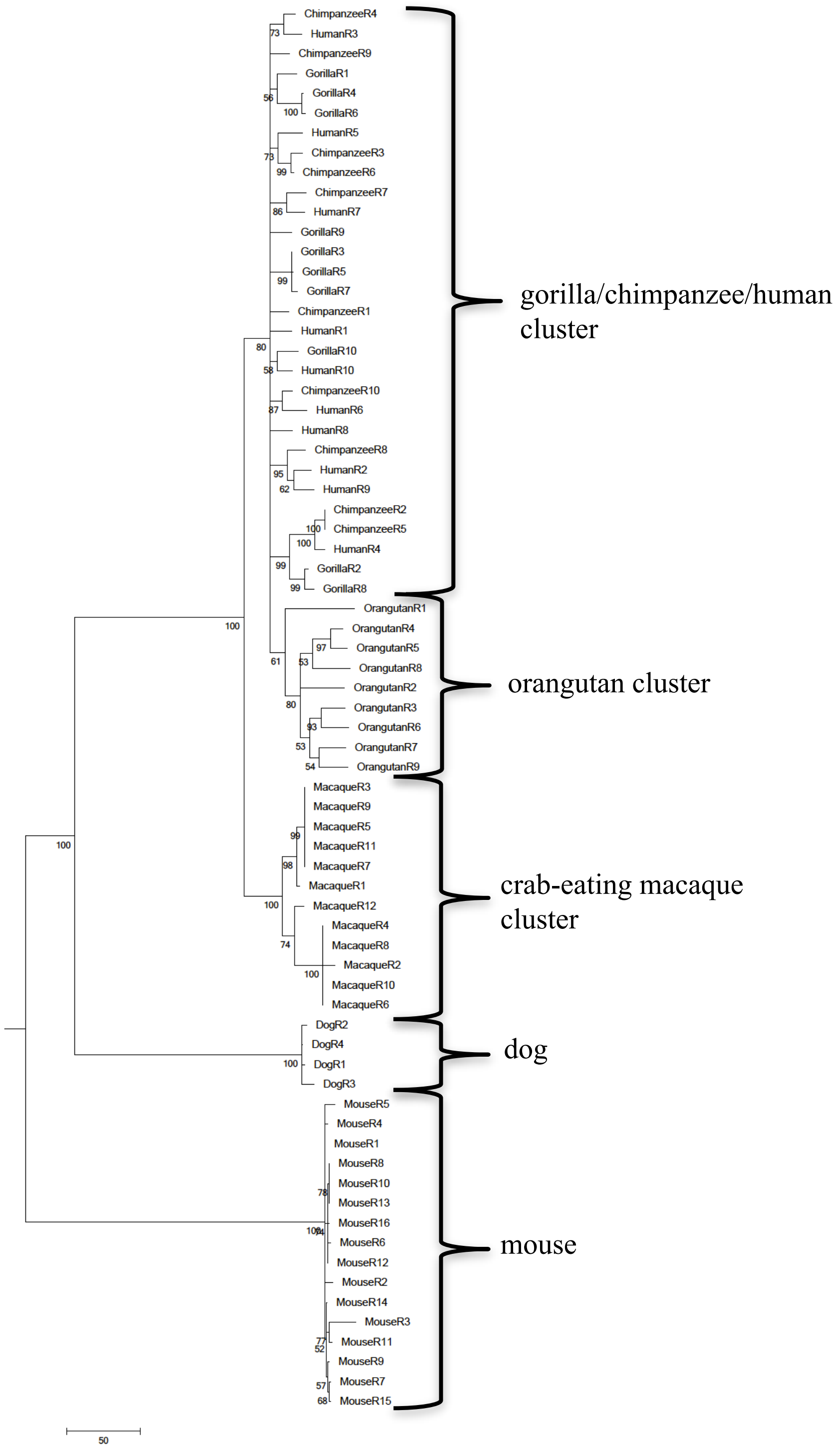
ChimpanzeeR4
HumanR3
ChimpanzeeR9
GorillaR1
GorillaR4
GorillaR6
HumanR5
ChimpanzeeR3
ChimpanzeeR6
ChimpanzeeR7
HumanR7
GorillaR9
GorillaR3
GorillaR5
GorillaR7
ChimpanzeeR1
HumanR1
GorillaR10
HumanR10
ChimpanzeeR10
HumanR6
HumanR8
ChimpanzeeR8
HumanR2
HumanR9
ChimpanzeeR2
ChimpanzeeR5
HumanR4
GorillaR2
GorillaR8
OrangutanR1
OrangutanR4
OrangutanR5
OrangutanR8
OrangutanR2
OrangutanR3
OrangutanR6
OrangutanR7
OrangutanR9
MacaqueR3
MacaqueR9
MacaqueR5
MacaqueR11
MacaqueR7
MacaqueR1
MacaqueR12
MacaqueR4
MacaqueR8
MacaqueR2
MacaqueR10
MacaqueR6
DogR2
DogR4
DogR1
DogR3
MouseR5
MouseR4
MouseR1
MouseR8
MouseR10
MouseR13
MouseR16
MouseR6
MouseR12
MouseR2
MouseR14
MouseR3
MouseR11
MouseR9
MouseR7
MouseR15
gorilla/chimpanzee/human cluster
orangutan cluster
crab-eating macaque cluster
dog
mouse
50

D.

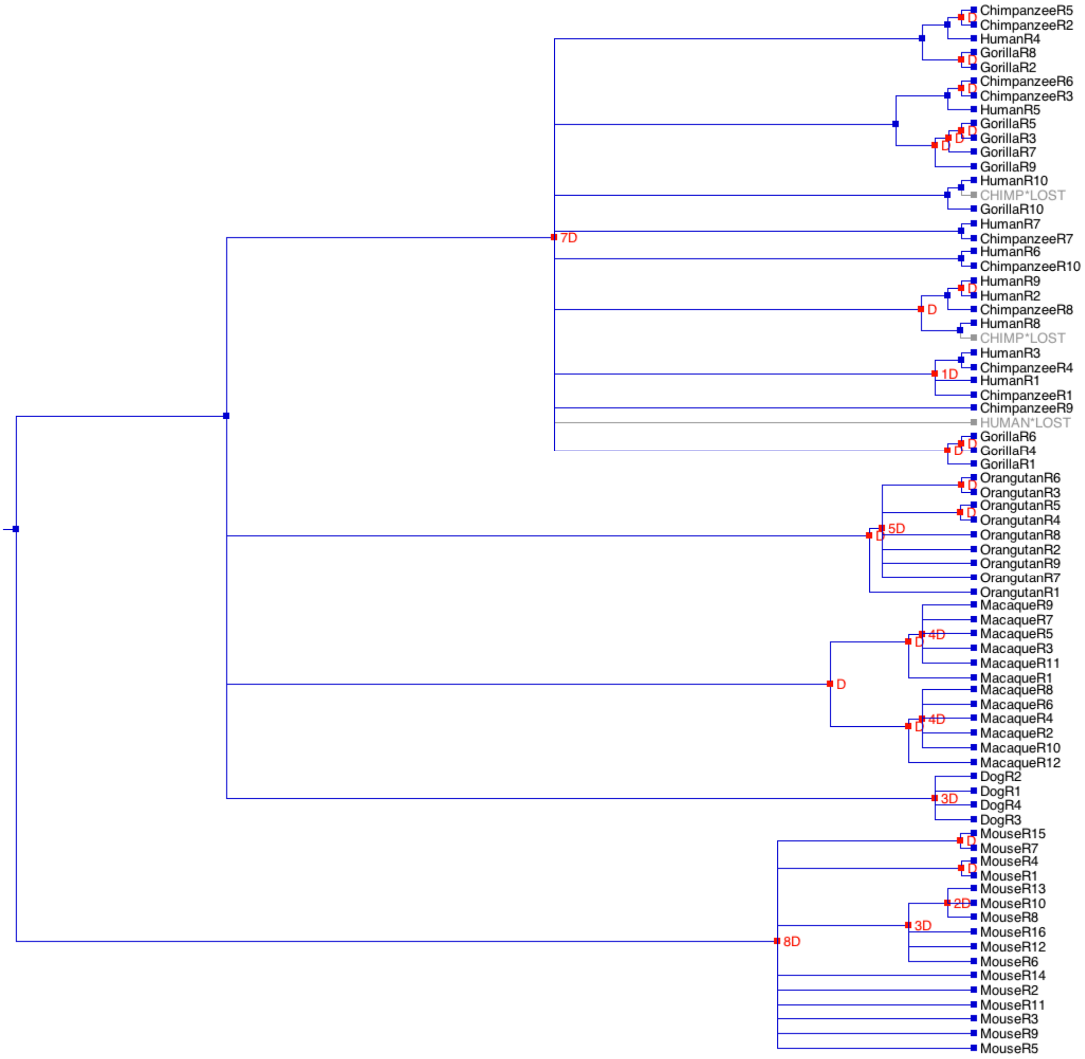
ChimpanzeeR5
ChimpanzeeR2
HumanR4
GorillaR8
GorillaR2
ChimpanzeeR6
ChimpanzeeR3
HumanR5
GorillaR5
GorillaR3
GorillaR7
GorillaR9
HumanR10
CHIMP*LOST
GorillaR10
HumanR7
ChimpanzeeR7
HumanR6
ChimpanzeeR10
HumanR9
HumanR2
ChimpanzeeR8
HumanR8
CHIMP*LOST
HumanR3
ChimpanzeeR4
HumanR1
ChimpanzeeR1
ChimpanzeeR9
HUMAN*LOST
GorillaR6
GorillaR4
GorillaR1
OrangutanR6
OrangutanR3
OrangutanR5
OrangutanR4
OrangutanR8
OrangutanR2
OrangutanR9
OrangutanR7
OrangutanR1
MacaqueR9
MacaqueR7
MacaqueR5
MacaqueR3
MacaqueR11
MacaqueR1
MacaqueR8
MacaqueR6
MacaqueR4
MacaqueR2
MacaqueR10
MacaqueR12
DogR2
DogR1
DogR4
DogR3
MouseR15
MouseR7
MouseR4
MouseR1
MouseR13
MouseR10
MouseR8
MouseR16
MouseR12
MouseR6
MouseR14
MouseR2
MouseR11
MouseR3
MouseR9
MouseR5
7D
D
1D
5D
4D
4D
3D
2D
3D
8D

Figure S5. Neighbor-joining tree reconstruction using complete repeats of the filaggrin gene in five primate species. Repeats that were found to have a possible recombinant region or gene conversion were excluded. The following parameters were used: pairwise deletion, proportional nucleotide differences, 1,000 bootstrap resampling, and a cutoff value of 50%. Most of the topology remained the same as shown in Supplementary figure S3A.

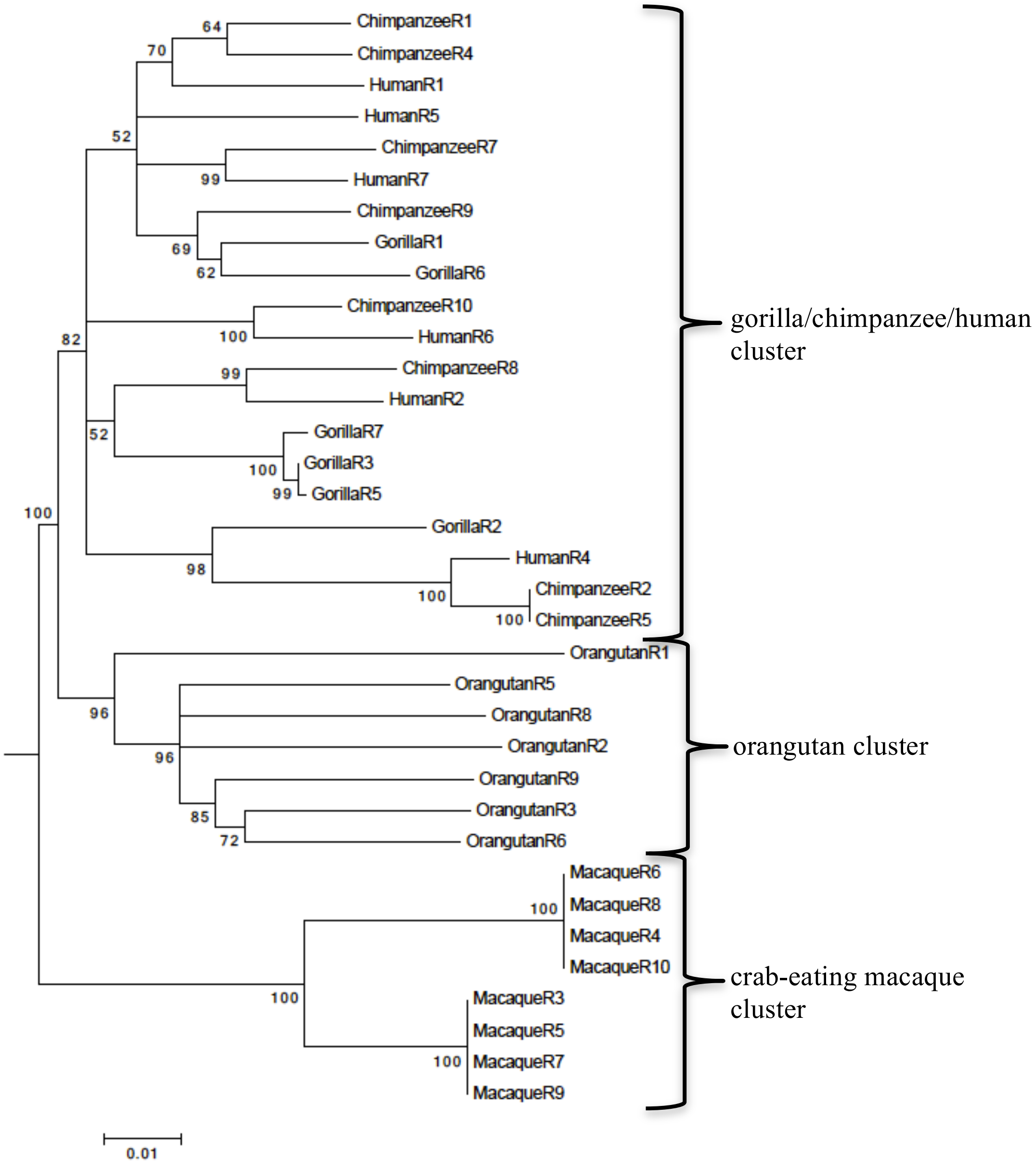

Figure S6. Gel electrophoresis picture of filaggrin repeated regions of all primates. Primers used are described in Table S1. Ladder used is λ DNA *Hin*d *III*. Human repeated region vary from 12000 to 14000 bp depending on the number of repeats. Repeat variation is also seen in chimpanzee and macaque. **Upper** Gel electrophoresis sample order: Chimpanzee GAIN ID: 0143, 0212, 0158, 0170, 0204, 0211, 0131, 0279, 0169, 0276, 0159 0345, and Primate ID: 954 and 956. **Middle** Gel electrophoresis sample order: Gorilla Primate ID: 1943 and 3846, and GAIN ID:0080, Orangutan Primate ID: 2541 and GAIN ID: 0091, 0031, 0110 and 0010, *Macacca fascicularis* ID:181 and 246 and *Macacca mulatta* ID: 725 and 970. **Lower** Gel electrophoresis sample order: First four samples include PCR product for human repeated region: homozygote for 12 repeats, heterozygote for 11 and 12 repeats, heterozygote for 10 and 11 repeats, heterozygote for 10 and 12 repeats. Final four samples shows the repeat variation region: homozygote for 12 repeats, heterozygote for 11 and 12 repeats, heterozygote for 10 and 11 repeats, heterozygote for 10 and 12 repeats.

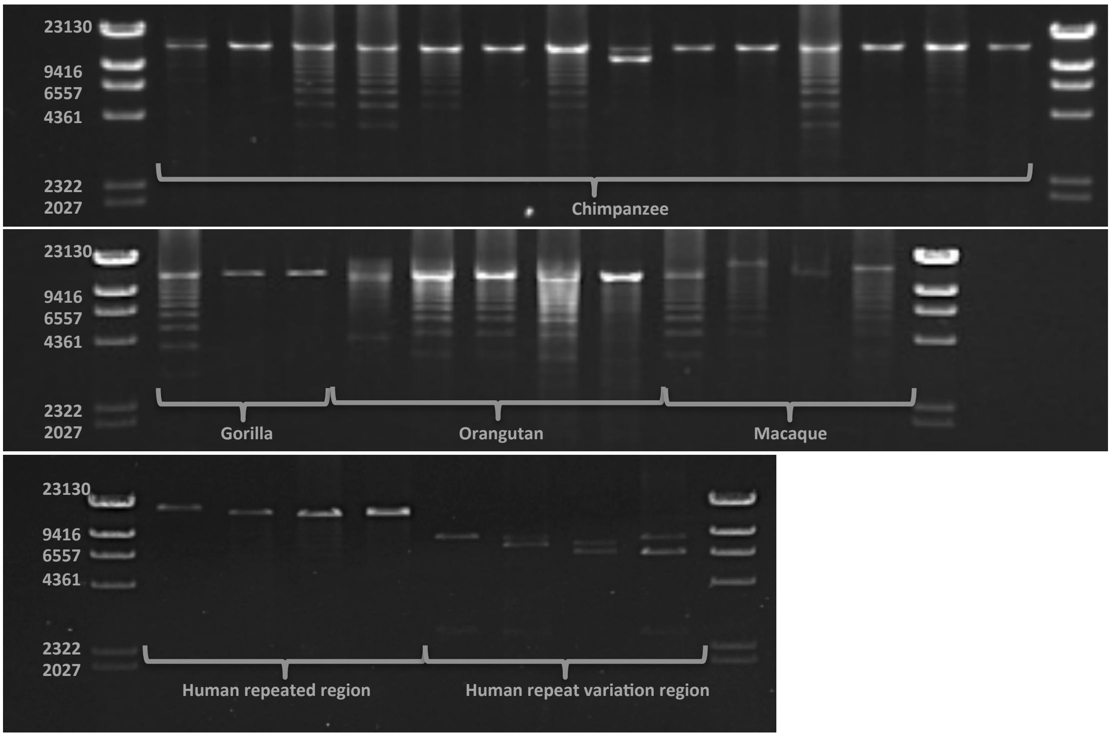

Supplement: Additional file 1: — Additional tables and figures. (PDF 3.32 kb) [file 12862_2016_851_MOESM1_ESM.pdf]
